# Temperature-dependent hydrogen deuterium exchange shows impact of analog binding on adenosine deaminase flexibility but not embedded thermal networks

**DOI:** 10.1016/j.jbc.2022.102350

**Published:** 2022-08-04

**Authors:** Shuaihua Gao, Wenju Zhang, Samuel L. Barrow, Anthony T. Iavarone, Judith P. Klinman

**Affiliations:** 1 Department of Chemistry, University of California, Berkeley, Berkeley, California, USA; 2 California Institute for Quantitative Biosciences, University of California, Berkeley, Berkeley, California, USA; 3 David R. Cheriton School of Computer Science, University of Waterloo, Waterloo, Ontario, Canada; 4 Department of Molecular and Cell Biology, University of California, Berkeley, Berkeley, California, USA

**Keywords:** adenosine deaminase, conformational landscape, protein flexibility, temperature-dependent hydrogen deuterium exchange (TDHDX-MS), thermal activation, DAA, 1-deaza-adenosine, HDPR, 6-hydroxyl-1,6-dihydropurine ribonucleoside, HDX, hydrogen deuterium exchange, LC, liquid chromatography, mADA murine, adenosine deaminase, MS, mass spectrometry, TDHDX-MS, temperature-dependent hydrogen deuterium exchange mass spectrometry

## Abstract

The analysis of hydrogen deuterium exchange by mass spectrometry as a function of temperature and mutation has emerged as a generic and efficient tool for the spatial resolution of protein networks that are proposed to function in the thermal activation of catalysis. In this work, we extend temperature-dependent hydrogen deuterium exchange from apo-enzyme structures to protein–ligand complexes. Using adenosine deaminase as a prototype, we compared the impacts of a substrate analog (1-deaza-adenosine) and a very tight-binding inhibitor/transition state analog (pentostatin) at single and multiple temperatures. At a single temperature, we observed different hydrogen deuterium exchange-mass spectrometry properties for the two ligands, as expected from their 10^6^-fold differences in strength of binding. By contrast, analogous patterns for temperature-dependent hydrogen deuterium exchange mass spectrometry emerge in the presence of both 1-deaza-adenosine and pentostatin, indicating similar impacts of either ligand on the enthalpic barriers for local protein unfolding. We extended temperature-dependent hydrogen deuterium exchange to a function-altering mutant of adenosine deaminase in the presence of pentostatin and revealed a protein thermal network that is highly similar to that previously reported for the apo-enzyme (Gao et al., 2020, JACS 142, 19936-19949). Finally, we discuss the differential impacts of pentostatin binding on overall protein flexibility *versus* site-specific thermal transfer pathways in the context of models for substrate-induced changes to a distributed protein conformational landscape that act in synergy with embedded protein thermal networks to achieve efficient catalysis.

Enzymes are nature’s best catalysts, achieving up to 10^30^-fold rate accelerations in comparison to the analogous uncatalyzed reactions (1, 2, 3, 4, 5). There has been increasing interest in how the vast array of thermally activated enzymes bring about large reductions in the enthalpic barrier for catalysis relative to their small molecule, uncatalyzed counterparts (6, 7, 8, 9, 10, 11). Enzymes are anisotropic structures, with active sites for chemical reactivity that are generally protected from direct collisions with solvent, implicating the protein scaffold as the basis for controlled heat transfer from the solvent bath to an enzyme active site.

Regional changes in temperature-dependent hydrogen deuterium exchange mass spectrometry (TDHDX-MS) upon protein perturbation have emerged as a generic and efficient experimental probe to uncover protein-based thermal networks that may function in catalysis. Using the EX-2 condition (*k*_HDX_ = K_op_ *k*_int_, where *k*_int_ represents the intrinsic rate constant for hydrogen deuterium exchange (HDX) within a transiently unfolded region of protein and K_op_ is the equilibrium constant for local protein unfolding), functionally related local protein motions can be deduced from the impact of time, temperature, and mutation on deuteron uptake to the peptide backbone. Studies of this kind have recently uncovered long-distance, site-specific pathways for thermal activation within a range of enzyme systems that include alcohol dehydrogenases (7), lipoxygenases (8), catechol O-methyltransferase (9), and the TIM barrel enzymes yeast enolase (10) and murine adenosine deaminase (11).

Each of the above studies was conducted in the absence of a bound substrate or substrate analog, raising the question of whether the studies of apo-enzyme structures are representative of the thermal pathways that contribute to the catalytic cycle. In this study, we employ the TIM barrel enzyme murine adenosine deaminase (mADA) as a prototype to examine TDHDX-MS for an enzyme in complex with either a substrate analog or a tight binding inhibitor/transition state analog. As a well-studied enzyme, mADA has a defined chemical mechanism that involves addition of a zinc-bound hydroxide ion to the C-6 position of the substrate purine ring to form a tetrahedral intermediate; subsequent expulsion of ammonia leads to restoration of an sp^2^ center in the product inosine (Fig. 1*C*) (12). Numerous X-ray structures (Fig. 1, *A* and *B*) are available for this enzyme system that include the apo-enzyme as well as an array of enzyme inhibitor complexes (13, 14, 15). The latter include 1-deaza-adenosine (DAA), a nearly ideal “ground-state” analog, having all the attributes for molecular recognition as a substrate with the notable exception of the absence of a nitrogen at N-1 that is positioned to hydrogen bond to the general acid, Glu217 (*K*_i_ = 1.8 × 10^−7^ M, ca. 100 times smaller than the K_M_ of adenosine (13)). An alternate, well studied compound is 2′-deoxycoformycin (pentostatin) that combines features of the tetrahedral intermediate formed at C-1 of substrate, while retaining the N-1 within the one carbon expanded ring structure. Pentostatin is a powerful inhibitor of mADA with a 10^6^-fold higher affinity than DAA, *K*_i_ ∼ 10^−13^ M (14) (see Fig. 1*D*).

**Figure 1:**
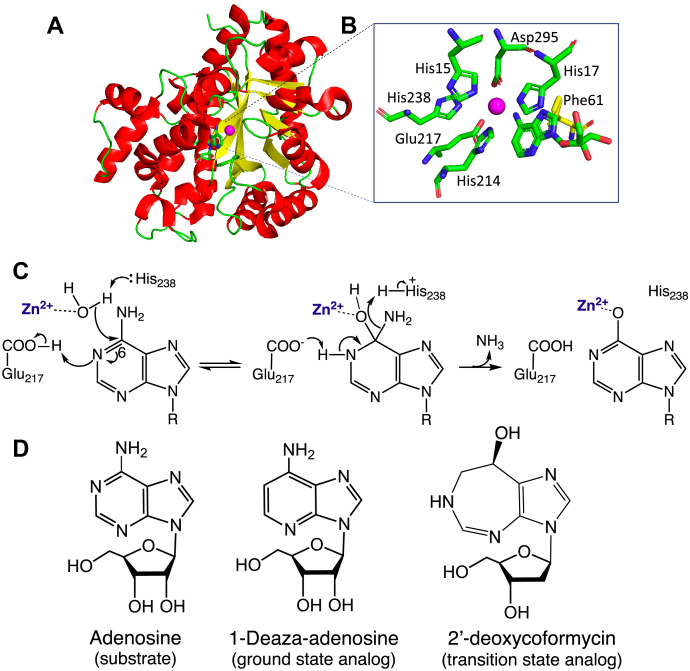
**Structure, catalytic mechanism, and ligands of mADA.***A*, the overall structure of mADA in complex with 1-deaza-adenosine (PDB:1ADD), with active site residues shown in (*B*) where zinc ion is colored *magenta* and the mutation position Phe61 for the study is shown in *yellow stick*. *C*, proposed catalytic mechanism of mADA in which the active site base His238 converts zinc-bound water to hydroxide; attack of hydroxide ion at C-6 of substrate, concomitant with proton transfer from Glu217 to N-1 of the purine ring, leads to the tetrahedral adduct (see details in reference 12); R group represents the ribose ring of substrate. *D*, chemical structures of the substrate, ground state analog (1-deaza-adenosine, DAA), and tight binding inhibitor/transition state (2′-deoxycoformycin, pentostatin) for mADA. DAA, 1-deaza-adenosine; mADA, murine adenosine deaminase.

In the presented study, the earlier TDHDX-MS investigation of WT and mutant forms of apo-adenosine deaminase (11) has been extended to enzyme in complex with either DAA or pentostatin. These ligands are seen to generate different patterns for HDX protection at a single temperature, as expected from their large differences in affinity for mADA. By contrast, a generic impact of bound ligand emerges from TDHDX, indicating most generally increases in the enthalpic barrier for local unfolding throughout the protein. Extension of studies of TDHDX to a functionally impactful mutant of enzyme in the presence of bound ligand further indicates a conservation of the thermal transfer protein network previously deduced from apo-mADA studies (11). The aggregated data support the presence of pathways for thermal activation in proteins that can be inferred from studies of either apo-protein or liganded complexes and are intrinsic to a protein’s structure and dynamics. We discuss catalysis in the context of two orthogonal dynamical coordinates: the first is a widely distributed conformational landscape, comprised of a very large number of protein substates that interconvert rapidly near room temperature and are sensitive to bound ligands; the second coordinate is ascribed to site-specific, protein-embedded pathways that provide directionality to heat transfer from the solvent bath in the initiation of active site chemistry.

## Results

### Single temperature investigation of protection against HDX by binding of the substrate analog (DAA) and a tight binding inhibitor (pentostatin) to mADA

**Figure 2:**
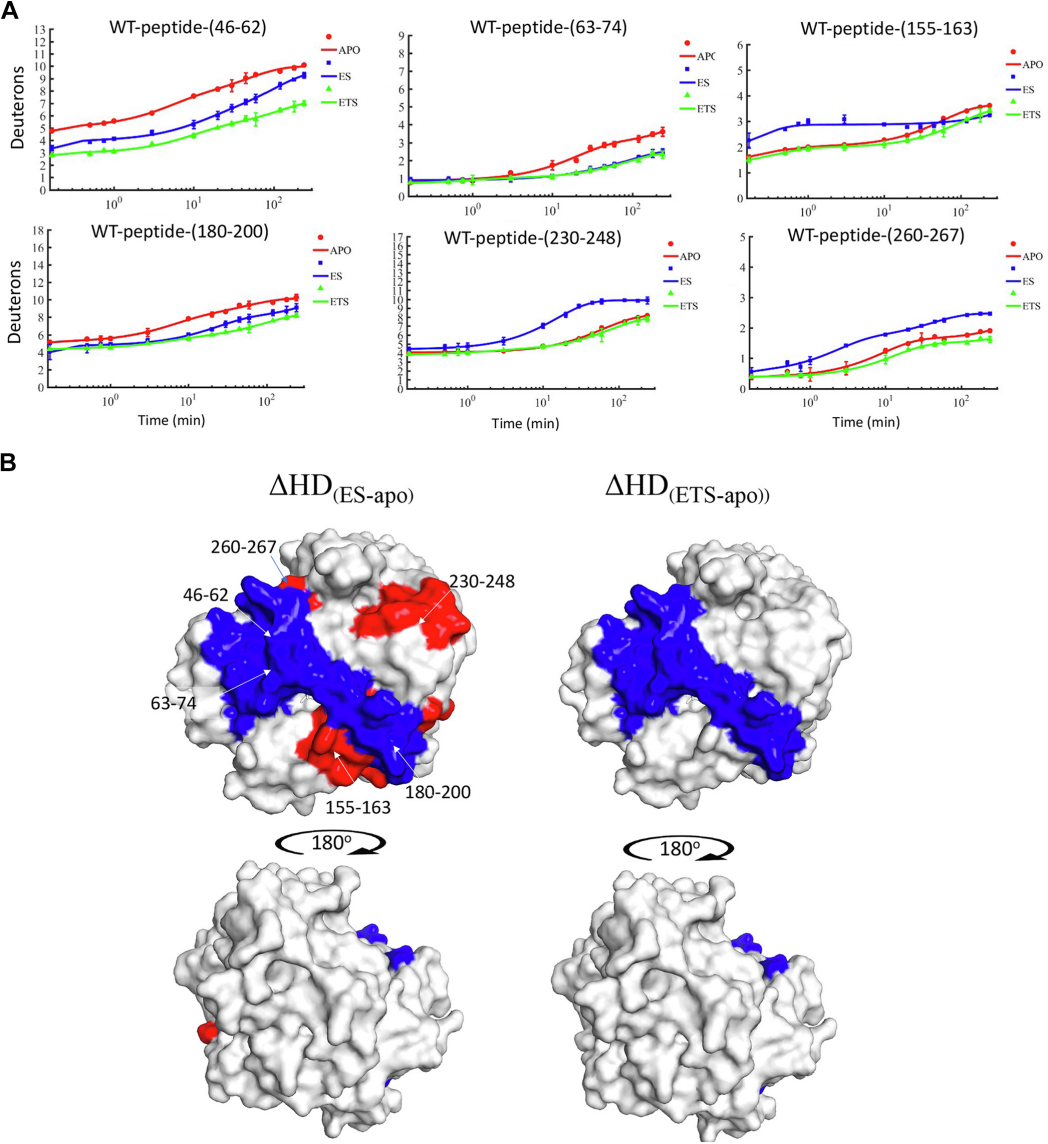
**Deuteron uptake comparisons between ligand free, ground state analog (ES), and tight binding inhibitor(ETS)bound statesfor WT mADA.***A*, HDX plots showing deuteron uptake differences between any two of the three states are presented. *Red*: ligand-free state; *blue*: ground state analog bound state; *green*: tight binding inhibitor bound state. *B*, dual-color mapping (*blue* and *red*, *blue* indicates less deuteron uptake and *red* indicates more deuteron uptake) was used to show HDX changes for ground state analog relative to ligand-free enzyme states (*left*) and for tight binding inhibitor bound relative to ligand-free enzyme states (*right*). HDX, hydrogen deuterium exchange; mADA, murine adenosine deaminase.

HDX mass spectrometry is increasingly applied to a wide variety of biological questions (16, 17, 18, 19, 20, 21, 22, 23, 24, 25). Traditionally, an HDX experiment is performed at a single temperature to examine changes triggered in the targeted protein by ligand binding or protein–protein complex formation. In this study, we first analyzed time traces for deuteron uptake in the presence of the ground state analog DAA for purified WT mADA at a single temperature (Fig. 2). Experiments were conducted at pH 7.3, 30 °C (14 time points: 0, 10, 30, 45, 60, 180, 600, 1200, 1800, 2700, 3600, 7200, 10800, and 14400 s), with analysis focused on the same 23 nonoverlapping peptides previously identified and analyzed for apo-mADA (11). Sequences of these 23 nonoverlapping peptides are provided in Fig. S1 and Table S1 and time traces of the 23 nonoverlapping peptides in the presence of either DAA or pentostatin are shown in Fig. S2. For each of the peptides, the number of incorporated deuterons is obtained by calculating the peptide mass change before and after the exchange process, following correction for back-exchange during peptide separation and analysis (see Table S2 for peptide-specific back-exchange values). Six peptides indicate an impact of DAA binding to WT mADA, with comparative time traces shown in Figure 2*A* and differences mapped onto a space filling model of mADA, Figure 2*B* (left). These impacted peptides include peptide 46-62, peptide 63-74, peptide 155-163, peptide 180-200, peptide 230-248, and peptide 260-267, three of which show clear cut protection by DAA (peptides 46–62, 63–74, and 180–200) and three of which indicate higher deuteron uptake relative to apo-enzyme: peptides 155-163, 230-248**,** and 260-267 (cf. Table 1). To ensure that enzyme was fully complexed with DAA throughout the course of the experiments, HDX was repeated at a 4-fold increase in analog concentration, generating similar results (Fig. S3).

**Table 1:** Peptides showing either increased (red tone) or decreased (blue tone) HDX between different states of WT mADA or F61A mADA **at 30** °**C** where ES **represents the complex of enzyme with ground state analog and ETS represents the complex of enzyme with tight binding analog**

^*a*^ Not detected.

The increased solvent accessibility in the DAA complex was unexpected and could be representative of structural rearrangements within the protein. X-ray crystallographic structures are available for comparison of the complex of mADA with DAA in relation to both pentostatin and another tight binding inhibitor (6-hydroxyl-1,6-dihydropurine ribonucleoside (HDPR), *K*i = 10^−13^ M (14)). One important feature of DAA is its inability to hydrogen bond to and anchor the active site acid, Glu217. This is corroborated from existing X-ray structures (see Table S3), where the distance between the C-2 of DAA and Glu217 is increased to 3.3 Å relative to pentostatin and HDPR, both of which indicate distances of 2.8 Å when nitrogen is preserved at the 2-position of the bound analog. These data suggest regions of increased solvent accessibility with DAA that are a consequence of structural perturbations that originate in the region of Glu217 and radiate out into adjacent regions of the protein. Specifically, peptide 230-248 contains the active site base His238 that is in hydrophobic contact with peptide 201-229 that harbors Glu217 (Fig. S4*A*). Residue Glu260 from peptide 260-267 forms a backbone hydrogen bond with His238 from peptide 230-238 that, as noted, is in hydrophobic contact with Glu217. A careful inspection also shows that residue Arg156 from peptide 155-163 undergoes H-bonding from its backbone to a zinc ligand (His214) proximal to Glu217 (Fig. S4*B*). The above analysis indicates how a seemingly minor alteration in the structure of an inhibitor can have multiple impacts on HDX.

With the goal of examining possible changes in the solvent accessibility of mADA as the enzyme moves from a substrate-like to a transition state-like complex, HDX was repeated with the very tight binding inhibitor of mADA, pentostatin at 30 °C (Fig. 2, *A* and *B* (right)). In this instance, only the three peptides that show protection from HDX with DAA indicate significantly altered time courses for deuteron incorporation in the presence of pentostatin relative to apo-enzyme: peptides 46-62, 63-74, and 180-200. We note that pentostatin is not a perfect transition state analog, possessing perturbations within the purine ring that has been expanded by one carbon unit and lacking a hydroxyl group at the C-2′ position of its ribose ring. However, the addition of this subnanomolar-binding inhibitor retains a key interaction lacking in DAA, that is, the ability to H-bond to Glu217. The pattern of protection produced by both pentostatin and DAA includes the site of substrate binding (peptide 46–74) and extends across the front face of the protein to encompass peptide 180-200.

**Figure 3:**
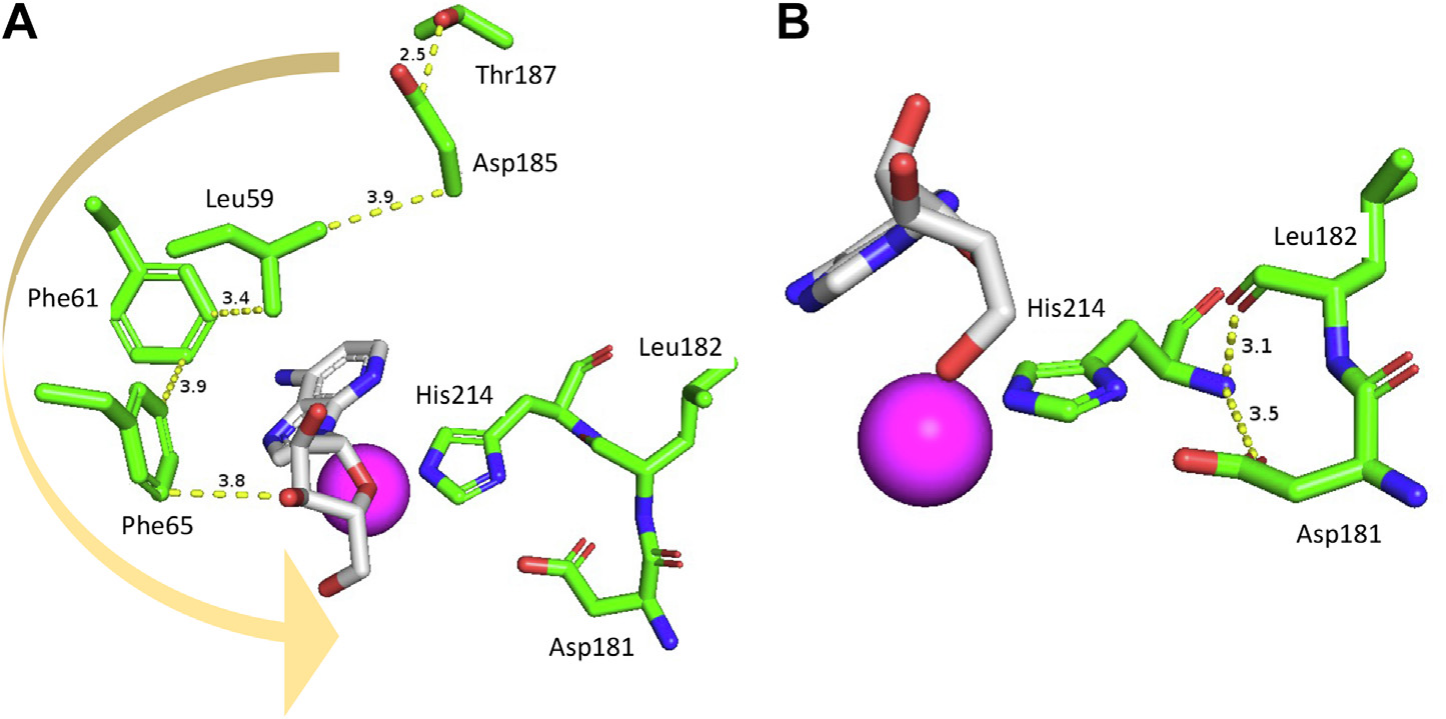
**Interactions in regions of peptide 180-200 (from the structure of mADA in complex with DAA).***A*, Asp185 (in H-bonding distance to Thr187) initiates a series of hydrophobic interactions that connect, in turn, Leu59, Phe61, and Phe65; the latter is in direct contact with the bound analog. *B*, Asp181 and Leu182 form hydrogen bonds with the zinc ligand His214, which is in contact with the bound analog. DAA, 1-deaza-adenosine; mADA, murine adenosine deaminase.

The involvement of peptide 180-200 in HDX protection for both DAA and pentostatin is of interest since this region does not enter into direct and discrete interactions with either bound ligand. We uncovered a network of interactions that connect Asp185 and Thr187 to the bound analog through a series of hydrophobic interactions, Figure 3*A*. As shown, the carbon of Asp185 initiates a hydrophobic network that extends to the “wall” of hydrophobic side chains, Leu59, Phe61, and Phe65, in direct contact with the back face of either bound analog. Additionally, the backbone carbonyl of Leu182 and side chain of Asp181 form hydrogen bonds to one of the catalytic Zn^2+^ ligands (His214) (Fig. 3*B*). It is clear that binding of both pentostatin (that is directly coordinated to the zinc ion) and DAA (that is positioned to undergo attack by zinc bound water) lead to a protection from HDX that radiates across the active site–containing face of the TIM barrel structure and terminates in opposing protein/solvent interface.

**Figure 4:**
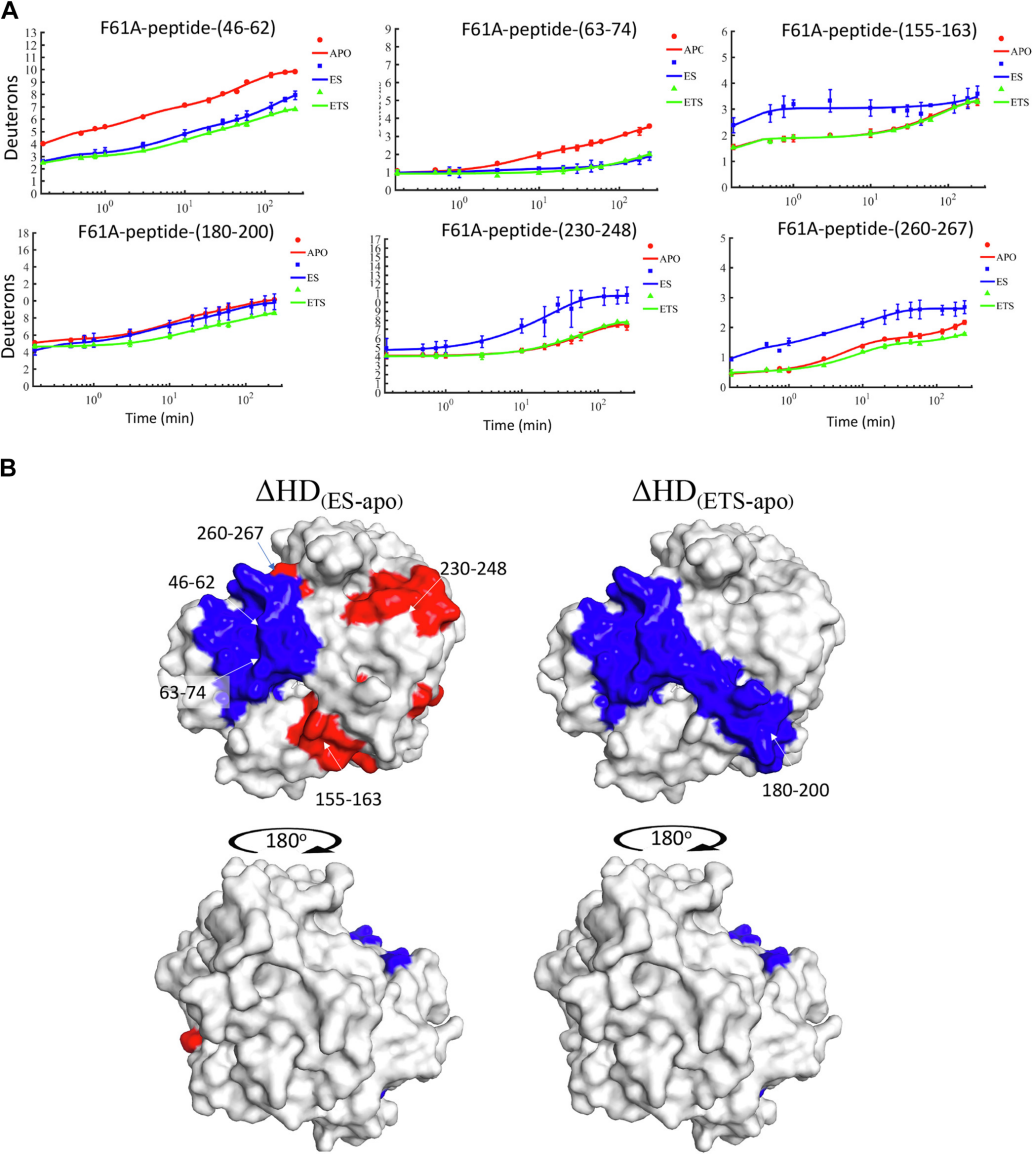
**Deuteron uptake comparisons between ligand free, ground state analog bound (ES), and tight binding inhibitor(ETS)bound statesto F61A mADA.***A*, HDX plots showing deuteron uptake differences between any two of the three states. *Red*: ligand-free state; *blue*: ground state analog bound state; g*reen*: tight binding inhibitor bound state. *B*, dual-color mapping (*blue* and *red*, *blue* indicates less deuteron uptake and *red* indicates more deuteron uptake) was used to show HDX changes for the DAA bound relative to free enzyme states (*left*) and tight binding inhibitor relative to free enzyme states (*right*). DAA, 1-deaza-adenosine; HDX, hydrogen deuterium exchange; mADA, murine adenosine deaminase.

Previous studies have shown that mutation at Phe61 (cf. Fig. 3*A*) leads to concomitant changes in the enthalpy of activation for active site chemistry and for regional local unfolding of the protein (11). In the context of differentiating regions of protein structure that undergo conformational accommodation upon substrate binding from site-specific heat transfer pathways inferred through the use of site-specific mutagenesis (see data below), we proceeded to examine the HDX properties of the purified F61A variant of mADA (Experimental procedures) in the presence of DAA and pentostatin at 30 °C (Fig. S5). In the case of DAA binding to F61A mADA, five peptides are detected that include peptide 46-62, peptide 63-74, peptide 155-163, peptide 230-248, and peptide 260-267 (Fig. 4*A*). Mapping these peptides onto the structure of mADA (Fig. 4*B*, left) shows considerable overlap to WT enzyme (Fig. 2*B*), with the exception of peptide 180-200. In contrast, the tight binding inhibitor pentostatin reveals protection for peptide 46-62, peptide 63-74, and peptide 180-200 in a pattern identical to WT protein, that is, largely independent of the site of mutation (Fig. 4*B*, right).

The failure to see protection in the region of peptide 180-200 from DAA complexation to F61A mADA is likely a combined outcome of the inability of DAA to H-bond to Glu217 (see above), and the observed 15-fold reduction in *k*_cat_ introduced by F61A that has been attributed to a resulting defect within the hydrophobic network positioned behind the bound substrate (see Fig. 3*A*). While F61A is also seen to reduce protection from pentostatin in this region (compare plots for peptide 180–200 in Figs. 2*A* vs. 4*A*), the tighter binding for this complex leads to residual protection against time-dependent deuteron uptake within peptide 180-200. The single temperature data indicate how competing trends of protein protection and increased solvent exposure emerge from a less than perfect substrate analog, as well as provide a consistent pattern of analog protection when using a tighter binding inhibitor.

### Extending single temperature studies of ligand protection against HDX to TDHDX

A study of HDX as a function of temperature (TDHDX), using a range of native enzymes and their functionally impactful mutants, has led to the identification of discrete anisotropic patches that enable thermal transfer from solvent surfaces to the active site (7, 8, 9, 10, 11). In the case of apo-WT mADA and its Phe61 variants, several regions of protein were resolved to be in the thermal activation pathways (11); these contain the substrate-binding site, the active site Zn^2+^, and assigned general acid and base side chains (Glu217 and His238, respectively). As a prelude to determining the TDHDX-derived thermal network for mADA in complex with substrate and transition-like ligands for comparison to the network observed using apo-mADA, we examined the differential impacts of temperature on the protection afforded by DAA and pentostatin on the WT enzyme. HDX was pursued at five temperatures (10, 20, 25, 30, and 40 °C) according to the protocols at 30 °C. Chromatographic retention times for each of the peptides remained the same as at 30 °C and the MS data confirmed EX-2 kinetics at all temperatures.

As reported previously (8, 10, 11), the determination of the energy of activation for HDX involves a time-dependent analysis of the primary HDX data according to a three-exponential equation, (Equation 1), as a function of temperature:

(1) $$y=N_{T}-A\cdot e^{-k_{1}\cdot t}-B\cdot e^{-k_{2}\cdot t}-C\cdot e^{-k_{3}\cdot t}-N_{NE}$$

where N_T_ stands for the total number of exchangeable amides within each peptide analyzed, and A, B, and C correspond to the numbers of amides exchanging with rate constants in the fast, intermediate, and slow regimes represented by *k*_1_, *k*_*2*_, and *k*_3_, respectively. N_NE_ represents the number of amides with no observable exchange within the experimental time period. Under the conditions of our experiments and as described in detail elsewhere (8, 10, 11), these analyses lead to a weighted average rate constant, [(B*k*_2_ + C*k*_3_)/N_T_], for each peptide at each experimental temperature. Subsequent Arrhenius analysis of the obtained rate constants provides activation barriers for HDX in a spatially resolved manner. Among the 23 analyzed peptides for WT mADA in the presence of DAA and pentostatin, 17 have been shown to yield measurable temperature-dependent trends (11). The remaining six peptides that lack measurable temperature dependencies during the experimental time window (peptides 1–14, 76–85, 98–108, 145–152, 291–300, and 321–344) are located either at the termini of the polypeptide chain or in the highly buried interior. These peptides display a single rapid exchange phase of predominantly low amplitude (with the exception of peptide 1–14). Full data sets for the HDX plots, Arrhenius plots, and fitted parameters are presented in Figs. S6 and S8 (top half) and Tables S4–S6.

**Figure 5:**
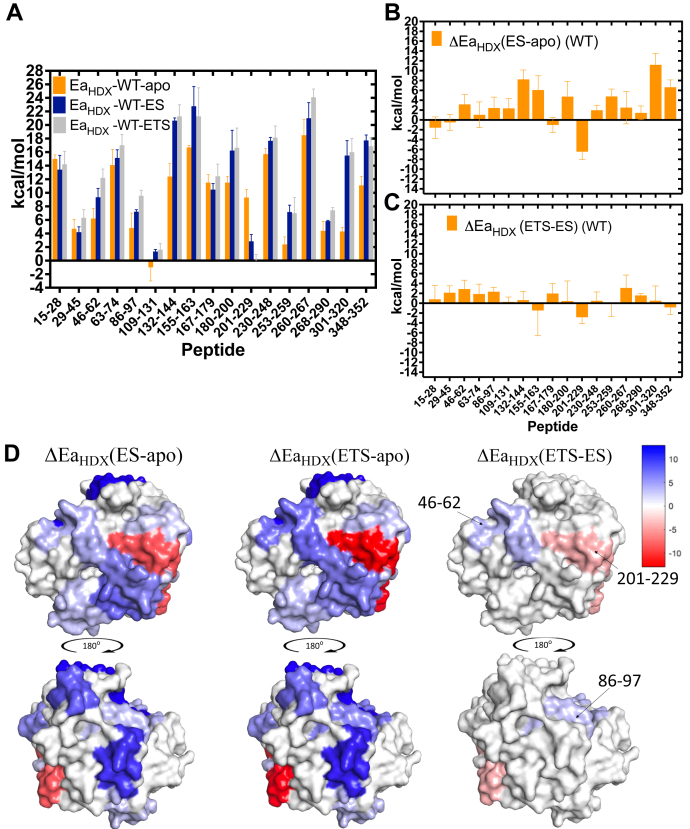
**Bar graph representations of the experimental activation energies Ea**_**HDX**_**for peptides from substrate free, DAA bound (ES), and pentostatin bound (ETS) WT mADA and mapping of ΔEa values on the structure of mADA.***A*, Ea_HDX_ values for each form of the protein. *B*, ΔEa (ES-apo). *C*, ΔEa (ETS-apo)_._ We note that in four out of fifty one Arrhenius plots, WT-apo-(**201–229**), WT-DAA-(**260–267**), WT-DAA-(**301–320**), and WT-pentostatin-(**301–320**), the 40 °C data point was anomalously low, possibly indicating a transitioning above 30 °C from intermediate HDX to the very fast HDX regime that reduces the weighted average rate constant; in these limited instances, fitting was restricted to 10, 20, 25, and 30 °C. *D*, structural mapping of (*left*) ΔEa (ES-apo)_,_ (*middle*) ΔEa (ETS-apo)_,_ and (*right*) ΔEa (ETS-ES). Heat maps are used to show the direction and magnitude of flexibility changes for regions that have become either more flexible (*red tones*) or more rigid (*blue tones*). The active site faces toward the reader in the *top three* structures. DAA, 1-deaza-adenosine; HDX, hydrogen deuterium exchange; mADA, murine adenosine deaminase.

Overall, 11 to 12 peptides display experimentally significant changes for ΔEa, when comparing apo-enzyme to the complexes of WT mADA with DAA or pentostatin, Figure 5, *A*–*C* and Table 2. The spatially resolved alteration in protein flexibility after binding of DAA or pentostatin is plotted onto space filling models of the structure of mADA (PDB:2ADA) in Figure 5*D*, (left and middle panels, respectively) using a heat map format that reflects the magnitude of the decrease (blue) or increase (red) in ΔEa_HDX_. With the exception of peptide 201-229, the binding of either ligand is seen *to decrease flexibility throughout both faces of the protein*, in contrast to the single temperature analysis in Figure 2. One key difference between the data in Figures 2 and 5 is that Figure 2 will reflect the free energy of binding, ΔG^o^ for each ligand, whereas the temperature dependence of HDX uncovers ΔH° ∼ Ea. We conclude that the latter is a closer approximation of changes in protein flexibility, whereas the former is a direct measure of the strength of ligand binding, determined by changes in both ΔS° and ΔH°.

**Table 2:** Peptides from WT mADA that show experimentally significant differences in Ea_HDX_ for E-DAA *versus* apo-mADA (labeled as ΔEa (ES-apo)), for E-pentostatin *versus* apo-mADA (labeled as ΔEa (ETS-apo)), and for E-pentostatin *versus* E-DAA (labeled as ΔEa (ETS-ES)

Peptide number	ΔEa (ES-apo)	ΔEa (ETS-apo)	ΔEa (ETS-ES)
**46–62**	3.15(2.0)	6.01(2.0)	**2.86(1.8)**
**86–97**	2.42(2.2)	4.76(2.3)	**2.34(0.9)**
109–131	2.35(2.0)	2.61(2.2)	0.26(1.0)
132–144	8.24(1.9)	8.88(2.6)	0.64(1.8)
155–163	6.07(2.9)	4.59(4.2)	−1.48(5.1)
180–200	4.73(3.1)	5.15(3.0)	0.42(4.1)
**201–229**	−6.46(1.6)	−9.3(1.5)	**−2.84(1.3)**
230–248	1.97(1.0)	2.45(1.9)	0.48(1.8)
253–259	4.77(1.5)	4.59(2.6)	−0.18(2.5)
301–320	11.2(2.3)	11.7(2.1)	0.5(3.0)
348–352	6.63(1.5)	5.77(1.7)	−0.86(1.4)

The three peptides that show statistically significant values for ΔEa (ETS-ES) are indicated in bold.

**Figure 6:**
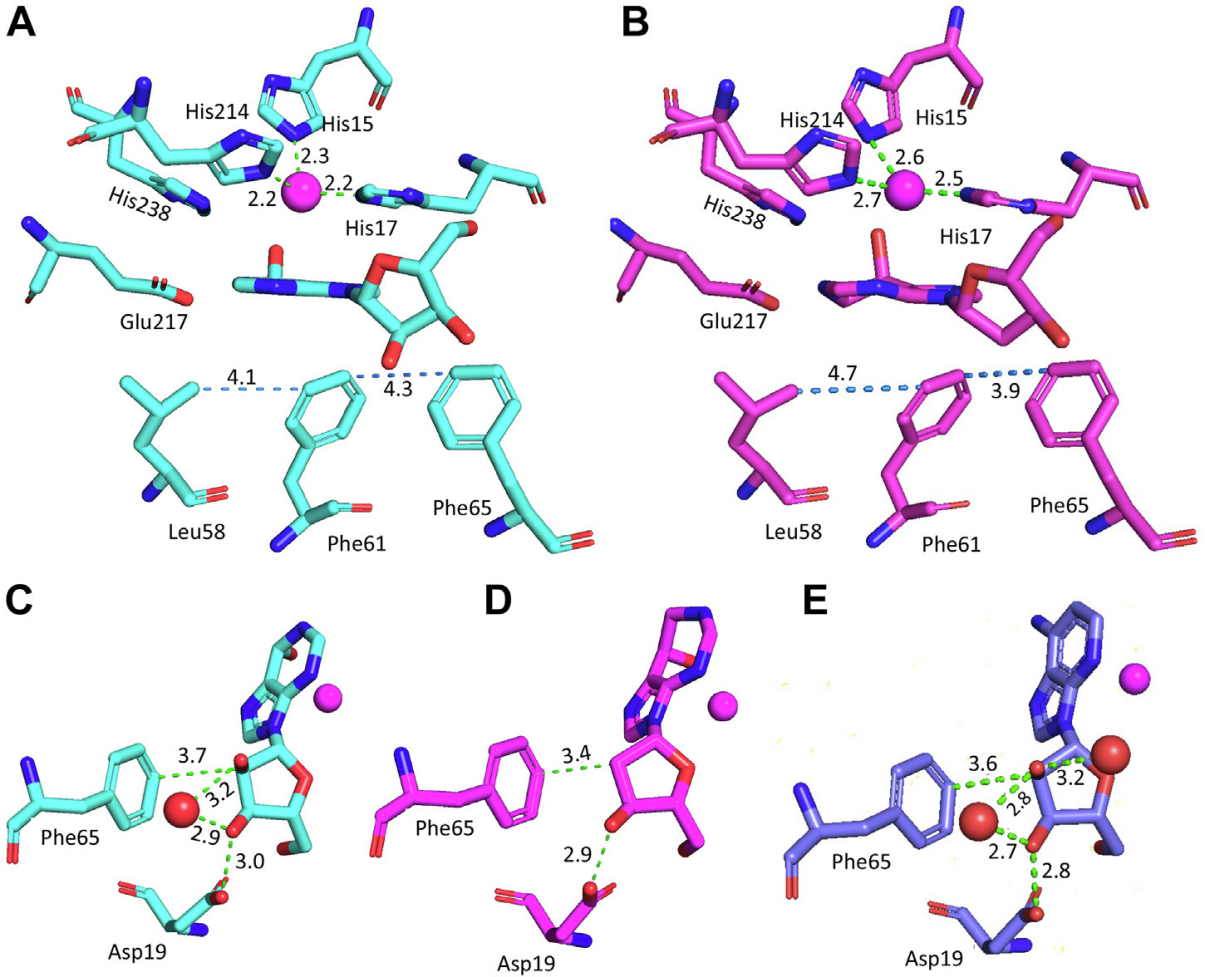
**Active site comparison of mADA in the presence of various ligands.** Comparison of active sites of HDPR (*A*) and pentostatin (*B*)-bound enzyme complexes. Distances (in Å) between the Zn^2+^ and its ligands are labeled in *green dashed* lines. Distances between the residues Phe58, Phe61, and Phe65 are labeled in *marine dashed* lines. Ribose moiety interactions in the HDPR complex (*C*), pentostatin complex (*D*), and DAA complex (*E*). DAA, 1-deaza-adenosine; HDPR, 6-hydroxyl-1,6-dihydropurine ribonucleoside. The zinc ion is labeled magenta and bound water molecules are indicated as *red spheres*.

Another important result to emerge from this comparative TDHDX analysis of DAA and pentostatin binding to mADA is that only three peptides show statistically significant ΔEa_HDX_ values for pentostatin in relation to DAA (Fig. 5, *C* and *D* (right) and Table 2) and these are in the range of only 2 to 3 (±1–2) kcal/mol. These small differences in ΔEa argue against a major change in protein flexibility in the presence of a tight binding *versus* substrate analog of mADA, despite the 10^6^-fold greater binding of pentostatin to mADA (12, 14). Among the three impacted peptides (46–62**,** 86–97, and 201–229, see Table 2), peptides 46-62 and 86-97 show decreased flexibility, whereas peptide 201-229 indicates an opposite trend (Fig. 5*D*, right panel). The available X-ray structures for apo-mADA (PDB:3MVI), the complex of mADA with DAA (PDB:1ADD), and the complex of mADA with pentostatin (PDB:1A4L), together with an additional structure of mADA with a second tight binding inhibitor ( HDPR (PDB:2ADA)), provide structural bases for each of the altered Ea values. Peptide 46-62 is located in a highly hydrophobic region of the mADA structure that, as shown in Figures 3*A* and S9*A*, is comprised of Leu58, Phe61, Leu62, and Phe65 that reside directly behind the plane of the purine ring of bound analogs. From a comparison of structures of mADA bound to pentostatin and HDPR, an analog that also binds to the active site zinc but retains the normal purine ring (Fig. 6, *A* and *B*), it can be seen that the expanded pyrimidine portion of the purine ring in pentostatin moves Phe61 closer to Phe65 by 0.4 to 0.6 Å and Leu58 moves away from Phe61 by 0.6 to 0.7 Å. These distance changes may be expected to impact local hydrophobic interactions and be the source of increased protein rigidification within the region represented by peptide 46-62 in the presence of pentostatin. The origin of the rigidification on the back face of the protein in the region of peptide 86-97 is less straight forward; however, we note that Phe86 interacts with Val16, adjacent to one of the Zn ligands His17, with pentostatin undergoing direct coordination to the zinc ion (Fig. S9*B*). Finally, peptide 201-229 is the single region to become more flexible. This behavior was seen previously from HDX analyses of two mutants of mADA at Phe61 that undergo a 15-fold reduction in *k*_cat_ (11). It appears that any alteration in the hydrophobic wall behind the bound ligand, either from site-specific mutagenesis at position 61 or the introduction of an analog that protrudes into this region, leads to the same result of an increase in protein flexibility within peptide 201-229. We conclude that this region that contains the active site acid, Glu217, must remain well structured for optimal catalysis, noting that the substrate analog DAA that is unable to accommodate an H-bond to Glu217 also introduces increased flexibility in this region (Fig. 5*D*, left).

### Determination of the thermal network in mADA in the presence of the tight binding inhibitor pentostatin

The pursuit of protein dynamical studies typically uses proteins in their native state, to avoid shifts in conformation as well as protection brought on by the presence of bound ligand(s). However, an unanswered question remains: whether thermal networks derived from apo-forms of an enzyme will be fully relevant to the catalytic E-S complex. In our previous work with mADA, temperature-dependent HDX experiments were conducted using apo-forms of WT enzyme and two variants: F61A that reduces both *k*_cat_ with an elevated Ea for catalysis and a control F61I that reduces *k*_cat_ to the same extent as F61A but leaves Ea unperturbed (11). The latter led to a refinement of the thermal network that eliminated perturbations unrelated to changes in the activation energy for catalysis (11). With the full set of TDHDX data available for both WT mADA and F61A (Figs. S6–S8 and Tables S4–S11) in the presence of a substrate analog (DAA) and transition state analog (pentostatin), we proceeded to construct a thermal network map of an enzyme-ligand complex. Noting that neither DAA nor pentostatin is a perfect analog of substrate or transition state, we chose to focus on pentostatin for several reasons. These include the following: (i) the structural perturbation introduced from DAA as detected from single temperature protection analyses and ascribed to the absence of N-1 in the purine ring of DAA that normally undergoes H-bonding to the active site acid, Glu217, (ii) the very similar patterns of changes in protein flexibility seen from the comparative TDHDX of DAA and pentostatin with WT enzyme, and (iii) the extraordinarily tight binding of pentostatin.

The relevant data for the differences in the enthalpy of activation between F61A and WT in the presence of pentostatin are presented in Table 3, along with the published data for apo-enzyme (11) for comparative purposes. The full set of analyzed peptides can be found in Table S12 and includes peptides eliminated from the present discussion due to the presence of experimental errors in excess of the observed Ea values in measurements of both free and ligand bound enzyme. The presented data include three peptides that were detected in the control mutation F61I with apo-enzyme and, hence, ruled out as contributing to a protein-based thermal conduit (underlined in Table 3).

**Table 3:** Peptides that show experimentally significant differences in ΔEa between F61A and WT for the apo state of mADA (27) or its pentostatin-bound state are in bold

Peptide number	ΔEa(F61A-WT) apo state	ΔEa(F61A-WT) ETS state
15–28	−**7.6(1.4)**	0.3(3.1)^a^
46–62	−**4.8(2.1)**	−**9.05(2.9)**
63–74	−**6.1(2.4)**^b^	−2.48(5.7)^a^
86–97	−1.4(2.7)^a^	−**4.5(1.9)**
155–163	−**3.3(0.9)**	−4.54(6.8)^a^
167–179	−2(1.3)	1.67(1.8)^a^
180–200	−**3.1(1.5)**	−1.77(3.4)^a^
201–229	−6.2(1.6)	−2.55(1.7)
230–248	−**3.3(1.0)**	−**5.34(3.3)**
260–267	−3.1(3.4)	−**4.83(2.8)**
268–290	−**5.1(2.1)**	−**5.46(2.6)**
301–320	2.9(1.4)	−1(2.3)^a^^,^^b^

**Figure 7:**
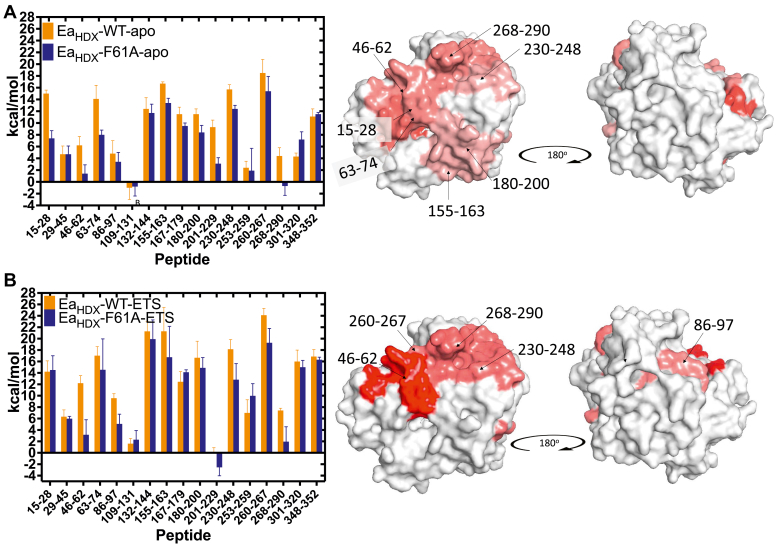
**Comparison of mADA thermal networks under different experimental conditions.** Derived thermal networks for (*A*) ΔEa_HDX_(F61A-WT) with apo-enzyme (27) and (*B*) ΔEa_HDX_(F61A-WT) for enzyme complexed to pentostatin (this work). The measured Ea values for WT and F61A are on the *left* and the mapping of the regions affected onto the WT mADA structure (PDB 2ADA) on the *right*. In the displayed thermal networks, heat maps are used to show the direction and magnitude of flexibility changes for regions that have become either more flexible (*red tones*) or more rigid (*blue tones*). The active site faces toward the reader in the left-hand structures. mADA, murine adenosine deaminase.

As discussed in the main text, the ΔEa(F61A-WT) apo-enzyme peptides **167-179**, **201-229**, and **301-320** (underlined) were eliminated from the final refined thermal network and are not mapped onto either structure in Figure 7. Peptides with indeterminate values of ΔEa in either one of the comparative states are labeled as such.

a Indeterminate.

b For these two peptides, Arrhenius plots indicated an anomalously low 40 °C data point and fitting was restricted to 10, 20, 25, 30 °C.

Inspection of Table 3 indicates a total of seven peptides with measurable changes in Ea for apo-WT mADA *versus* apo-F61A, and five peptides with changes for the pentostatin-WT mADA *versus* pentostatin-F61A (in bold). Among the seven peptides attributed to the thermal network in the apo-protein, four peptides show indeterminate values when enzyme is analyzed in the presence of pentostatin. Two of these peptides are near the purine-binding pocket with peptide 63-74 containing one of the Phe residues (Phe65) perturbed by the presence of the 7-member ring in pentostatin (Fig. 6, *A* and *B*) and peptide 15-28 adjacent to peptide 63-74 in the tertiary structure. The remaining two peptides that are indeterminate with pentostatin are peptide 155-163 and 180-200; these reside in the vicinity of the ribose ring of the bound pentostatin that lacks a hydroxyl group at the 2′ position. Comparison of the mADA-pentostatin X-ray structure to that for mADA bound to DAA or HDPR, an alternate inhibitor/transition state analog that retains the hydroxyl group at the C-2′ position, shows that the aberrant C-2′ position in pentostatin has caused Phe65 to move closer to the dehydroposition of its ribose ring by ∼0.2 to 0.3 Å, accompanied by the displacement of a bound water molecule (Fig. 6, *C*, *D* and *E*). It appears that the indeterminate positions observed for the pentostatin–mADA complex (peptides 15–28, 63–74, 155–163, and 180–200) correspond to regions of mADA perturbed as a result of the structural features of the pentostatin itself. We note that the prior evidence for an impact of F61A on ΔEa in apo-protein at positions of peptide 155-163 and peptide 180-200 had been weak (11).

The five peptides indicating experimentally substantiated changes to ΔEa due to F61A in the presence of pentostatin are mapped onto the tertiary structure of mADA in Figure 7*B*. As shown, this is represented by regions of protein residing behind the purine ring of bound analog (peptide 46–62) and containing the active site base His238 near the bound Zn (peptide 230–248), together with a connector region between the left- and right-hand portions of the thermal network (peptide 268–290). In Figure 7*A*, the previously published refined thermal network for apo-mADA is reproduced as a frame of reference (11). In both instances, the residues that change their activation energies upon replacement of Phe61 by Ala reside within the face of protein that comprises the active site, with very few alterations to the opposing, noncatalytic face of mADA. Of note, the regions found to undergo similar rigidification in the presence of either DAA or pentostatin bound to WT (Fig. 5*D*, left and center) are largely absent from the inferred thermal network in the presence of pentostatin (Fig. 7*B*).

Significantly, the collected TDHDX data of native mADA and of a mADA tight binding inhibitor complex indicate a conservation of mechanistically relevant components within their defined thermal networks. In both instances, opposing protein/solvent interfaces reach inward from the left toward the reactive purine ring of the bound substrate and from the right toward the adjacent active site metal and catalytic base. These findings provide experimental support for the premise of embedded thermal networks within mADA that are largely unchanged upon ligand binding and that can be expected to function in conjunction with distributed protein conformational landscapes that undergo adaptation upon binding of a substrate or relevant analog (See Ref (26) and discussion below).

## Discussion

### The multiple levels of information available from HDX experiments

Three tiers of HDX experiments have been described in this study, beginning with single temperature studies of WT and F61A that allow a distinction to be made about the level of protection from ligands that differ 10^6^-fold in their affinities for mADA. Specifically, at 30 °C, the weaker binding ground state analog DAA that lacks the ability to H-bond to the active site acid, Glu217, creates perturbations that lead to a combination of both protection in the region of the substrate-binding pocket and increased exchange in regions connected to the Glu217-binding site (Figs. 2*B*, left, and 4*B*, left). By contrast, an imperfect but significantly tighter binding inhibitor (pentostatin) is shown to produce clear and consistent trends of protection from exchange in both WT and F61A mADA (Figs. 2*B*, right and 4*B*, right).

The extension of single temperature investigations of HDX to multiple temperatures greatly increases the information content of HDX, with 11 to 12 peptides showing experimentally significant changes in ΔEa, calculated from a comparison of Ea values for WT apo-enzyme to Ea values for WT enzyme in complex with either DAA or pentostatin, Figure 5, *A*–*C* and Table 2. This described application of TDHDX uncovers similar changes in enthalpic barriers for local protein breathing modes of WT-mADA upon binding of both inhibitors (Fig. 5*D*). The displayed patterns are very distinct from the single temperature data that reflect contributions from both ΔS° and ΔH° to net ligand binding. We conclude that a favorable ΔS° is the primary determinant of the picomolar affinity of pentostatin 30 °C, consistent with its bis-substrate analog structure and behavior (27, 28).

Turning to one of the primary goals, TDHDX-MS was then used to examine whether the thermal transfer pathway reported for the apo-form of mADA (11) would be retained (or altered) in the presence of a tight-binding substrate/transition state analog. Focusing on the differences in TDHDX behavior of WT enzyme and F61A in complex with pentostatin has answered this question, showing very high retention of the primary features of the heat transfer conduit originally inferred for apo-mADA, Figure 7. While impactful information is available regarding thermally activated pathways from studies of apo-enzyme, in the case of mADA, this was derived from comparative TDHDX-MS studies of several Phe61 variants to eliminate contributions from these variants on *k*_cat_ in addition to the activation energy. The use of tight-binding inhibitor–enzyme complexes may speed up and simplify the identification of thermal networks from TDHDX analyses, eliminating the need for additional controls involving alternate site-specific mutants.

**Figure 8:**
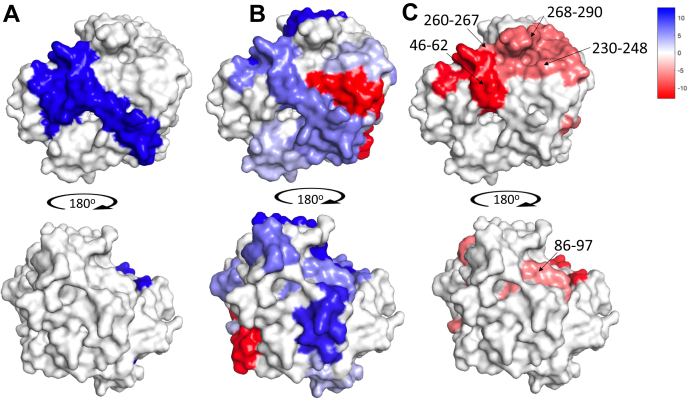
**Comparison of HDX results under different experimental conditions.** Heat maps are used to show the direction and magnitude of flexibility changes for regions that have become either more flexible (*red tones*) or more rigid/protected (*blue tones*). *A*, protection of mADA from HDX upon binding of the tight binding inhibitor pentostatin at a single temperature; *B*, the impact of pentostatin binding on protein flexibility obtained from multiple temperature HDX experiments; and *C*, thermal network for catalysis deduced for mADA in the presence of pentostatin through a comparison of the functionally impaired mutation F61A to native enzyme. HDX, hydrogen deuterium exchange; mADA, murine adenosine deaminase.

In Figure 8, *A*–*C*, we summarize the aggregate HDX results obtained from single and multiple temperature studies of mADA in complex with pentostatin. A number of key features emerge on inspection. First, plots of the changes in the enthalpies of activation for HDX in the presence of pentostatin (Fig. 8*B*) indicate a ligand-induced decrease in protein flexibility throughout a large portion of the protein that includes both the catalytic face (shown in the top middle structure) and the opposing face of the protein (bottom middle structure). Focusing on the catalytic face of mADA, we observe a region that overlaps with the single temperature data (Fig. 8*A*); however, temperature-dependent studies greatly extend the information content of HDX to indicate the widespread impact of ligand on thermally activated protein flexibility throughout the entire protein. There is only one region in Figure 8*B* that undergoes an increase in flexibility in the presence of pentostatin, and this is attributed to an induced perturbation from the expanded ring structure of this inhibitor. It can be seen that DAA binding diminishes protein flexibility to almost the same extent as pentostatin (Fig. 5*D*, right). The primary impact of either ligand binding is to decrease the overall flexibility of the protein structure with little discernible difference between DAA and the tight binding/transition state analog pentostatin.

Another important and mechanistically rich conclusion comes from a comparison of the impact of pentostatin on global protein flexibility (Fig. 8*B*) and the impact of the functionally impairing F61A on the temperature sensitivity of protein fluctuations (Fig. 8*C*). First, it can be seen that the latter is restricted to the catalytic face of mADA, in marked contrast to the impact of ligand binding throughout the majority of the protein. Second, a new region emerges in Figure 8*C* that overlaps with the active site zinc ion, its ligands, and the active site base, together with a “connector region” (peptide 268-290) that links the protein/solvent interface defined by peptide 230-248 to the opposing protein/solvent interface located behind the substrate-binding site, peptide 260-267 and peptide 46-62.

### A two-coordinate model for the dynamical control of mADA

The rich information content from TDHDX provides physical insights into the origins of enzyme catalysis that go beyond the historical definition of enhanced “transition state binding” (29, 30, 31, 32, 33, 34). Notably, the inherent anisotropic topography of globular enzyme structures introduces the possibility of evolved and site-specific protein networks for thermal transfer from the solvent bath to active site components (7, 8, 35, 36, 37, 38). This property has, in fact, begun to emerge experimentally through the application of TDHDX to a growing number of different enzyme structures and reaction classes (8, 9, 10, 11). The ability of an enzyme to regulate heat transfer has the dual advantage of preventing nonproductive and disruptive thermal energy transfer while targeting the region of the protein directly involved in bond cleavage and formation, distinguishing biological catalysts from the reactivity of small molecule counterparts. In this study, we describe the use of a tight inhibitor binding for mADA to examine the impact of ligand binding on changes in protein flexibility (Fig. 8*B*) that can be distinguished from a structurally encoded protein thermal network (Fig. 8*C*).

Conformational dynamics are indispensable to enzyme function throughout the catalytic cycle (39, 40, 41, 42, 43, 44, 45), with conformational selection and shifts in the population of protein sub-states predicted to occur along the reaction path (46, 47, 48, 49, 50, 51, 52, 53, 54, 55, 56). As shown herein, ligand binding to mADA shifts the enzyme’s conformational landscape toward a more rigidified ensemble that is anticipated to enhance the alignment of functional groups and to shorten their distances in preparation for catalysis. We have further argued that thermal networks are an innate and embedded property of enzymes that remain approximately fixed throughout the catalytic cycle (8, 9, 10, 11). Interrogating the thermal network of mADA in the presence of a tight-binding inhibitor provides strong support for the network previously identified for mADA in its apo-form (11). The ability of TDHDX to differentiate the impact of ligand binding on overall mADA flexibility from the identified thermal network leads to a natural separation of the two dynamical coordinates. We note that while both conformational sampling and thermal conduction are expected to occur simultaneously in enzyme reactions, small enthalpic differences among rapidly equilibrating and distributed conformational substates may be expected to distinguish this behavior from the higher enthalpic barriers that are required for ensuing chemical reactions. Ongoing efforts are focused on examining the degree to which the two defined coordinates may be further differentiated using time-dependent spectroscopic measurements (26, 57, 58, 59).

## Experimental procedures

### Reagents

If not stated otherwise, all chemical reagents were purchased from commercial sources with the highest level of purity. The ground state analog DAA is from Apexbio Technology LLC. The transition state analog pentostatin (2′-deoxy-coformycin) is from Abcam Inc.

### Protein expression and purification

The WT and F61A mADA were expressed and purified as stated before (11). The purity of the protein was assessed by SDS-PAGE and intact mass spectrometry (MS). Protein concentration was determined using the Thermo NanoDrop 2000 at 280 nm. The concentration values obtained from NanoDrop were calibrated using the extinction coefficient calculated online (https://web.expasy.org/protparam/). The thermal stability of WT mADA and mutant was examined by incubating the proteins at 40 °C, which was the highest temperature for HDX and kinetic experiments. Incubation results showed that, at up to 4 h at 40 °C, WT and mutant proteins maintained activities higher than 95% based on *k*_cat_ calculation, ruling out any possible structural changes that can cause activity loss for the enzyme at higher temperatures.

### Peptide library generation

Fragments of WT mADA from pepsin digestion were analyzed using a Thermo Dionex UltiMate3000 RSLCnano liquid chromatography system (LC) that was connected in-line with an LTQ Orbitrap XL mass spectrometer equipped with an electrospray ionization source (Thermo Fisher Scientific). See (11) for details. All MS-related experiments were performed in UC Berkeley QB3/Chemistry Mass Spectrometry Facility.

### HDX experiment

To achieve higher than 99.9% ligand binding, 1-fold and 25-fold of transition state analog pentostatin (picomolar binding) and ground state analog DAA (micromolar binding) were incubated with enzyme to achieve more than 99.9% of bound complex in the course of the HDX experiments, respectively. A control experiment was also run at a higher temperature (40 °C), in which the ratio of DAA to enzyme was increased an additional 4-fold (100-fold excess of DAA relative to enzyme). No significant changes in HDX behavior were observed, in support of retention of the condition of DAA saturation at all temperatures. Purified WT (or F61A) mADA protein (200 μM) was thawed on ice. A tube with a volume of 2.5 μl protein and a tube with a volume of 2.5 μl 5 mM ground state analog DAA (or 2.5 μl 200 μM pentostatin) were put into the water bath for 10 s (Fisher Scientific, Isotemp 3016D) to be preheated to the designated temperature. Then a volume of 45 μl preequilibrated D_2_O buffer (50 mM potassium phosphate buffer, pD 7.3) was mixed with the preheated 2.5 μl protein solution and 2.5 μl DAA or pentostatin. The samples were incubated at five different temperatures (10, 20, 25, 30, and 40 °C) for different times spanning from seconds to hours (0, 10, 30, 45, 60, 180, 600, 1200, 1800, 2700, 3600, 7200, 10800, 14400 s). Upon the completion of the exchange reaction, the sample was first quenched in a −20 °C ice bath with salt and the subsequent addition of 20 μl acid (0.32 M citric acid, pH 2.4). A volume of 20 μl of 0.4 mg/ml pepsin was added to the mixture for the digestion of the protein into small peptides for 2 min. To expedite the pepsin digestion, 20 μl of guanidine (in 0.32 M citric acid, pH 2.4) was added to the samples prior to pepsin addition. After digestion, 60 μl of the exchanged sample was transferred to Agilent 250-μL polypropylene insert MS tubes and flash frozen in liquid nitrogen. Two biological replicates were performed for both DAA- or pentostatin-bound HDX experiments. All the procedures except for deuteration were performed on ice. For the justification of the reproducibility of using less than three replicates, please see (11).

### LC-MS for HDX measurement

Deuterated, pepsin-digested samples of transition state (ground state) analog complexes of mADA from HDX experiments were analyzed using an Agilent 1200 LC that was connected in-line with the LTQ Orbitrap XL mass spectrometer (Thermo). The LC was equipped with a reversed-phase analytical column (Viva C8, length: 30 mm, inner diameter: 1.0 mm, particle size: 5 μm, Restek) and guard pre-column (C8, Restek). Solvent A was 99.9% water/0.1% formic acid and solvent B was 99.9% acetonitrile/0.1% formic acid (v/v). Each sample was thawed immediately prior to injection (5 μl) onto the column. The elution program consisted of a linear gradient from 5% to 10% B over 1 min, a linear gradient to 40% B over 5 min, a linear gradient to 100% B over 4 min, isocratic conditions at 100% B for 3 min, a linear gradient to 5% B over 0.5 min, and isocratic conditions at 5% B for 5.5 min, at a flow rate of 300 μl/min. Mass spectra were acquired in the positive ion mode over the range *m*/*z* = 350 to 1800 using the Orbitrap mass analyzer, in profile format, with a mass resolution setting of 100,000 (at *m*/*z* = 400). Data acquisition was controlled using Xcalibur software (version 2.0.7, Thermo).

### Data analysis

The MS data confirm that the HDX process studied here follows apparent EX-2 kinetics in all cases, reflecting local and reversible protein unfolding where *k*_close_ ≫ *k*_int_. Chromatographic retention times for each of the peptides were constant throughout the MS study. For each of the peptides, the number of incorporated deuterons was obtained by calculating the peptide mass change before and after the exchange process, following correction for back-exchange during peptide separation and analysis. Mass spectral data acquired for HDX measurements were analyzed using the software, HDX WorkBench (http://hdxworkbench.com/). The same set of back exchange values from (11) for WT mADA protein is used to correct the deuteron uptake. The nonoverlapping 23 peptides from WT mADA were selected as peptide set and data from five temperatures were analyzed (Datasets S1 and S2). Peptide MS data were manually curated, focusing on peptide identification, noise, and retention time. The deuteron uptake was calculated for each of the 23 peptides. The values were normalized by the total numbers of amides (excluding proline residues) in each peptide and corrected for peptide-specific back-exchange (see Table S2). The data were plotted as deuterons *versus* time in log scale using MATLAB software (https://www.mathworks.com/products/matlab.html). The rates and extents of exchange were determined from three-exponential fits to the analyzed time-resolved HDX data. No bounds were set for the fittings, initial values of 2.5, 0.5, and 0.01 min^-1^ were set for *k*_1_, *k*_2_, and *k*_3_, respectively. Initial values for A, B, C, and N_NE_ were set as 0.25N_T_. For Ea_HDX_ calculation, the average of weighted average rate constants was used to generate Arrhenius-like plots. MATLAB code can be found through the link here: https://github.com/ShuaihuaGao/HDX-code. Datasets S1 and S2 contain the original HDX data and HDX summary, respectively.

## Data availability

All data are contained within the article and the supporting information. Material described is available upon request from the corresponding author.

## Supporting information

This article contains supporting information that includes Figs. S1 to S9, Tables S1 to S12 for the HDX-MS analyses, and Dataset S1 and Dataset S2 which contain the original HDX data and HDX summary (10, 11).

## Conflict of interest

The authors declare no conflict of interest.
