# Supplementary material for: Temperature-dependent hydrogen deuterium exchange shows impact of analog binding on adenosine deaminase flexibility but not embedded thermal networks
Source: J Biol Chem. 2022 Aug 4;298(9):102350. doi: 10.1016/j.jbc.2022.102350 (PMC9483566; doi:10.1016/j.jbc.2022.102350)
Supplement: Supplemental information [file mmc3.pdf]

Supporting Information for

Temperature-dependent hydrogen deuterium exchange shows impact of analog binding on adenosine deaminase flexibility but not embedded thermal networks

Shuaihua Gao[†‡], Wenju Zhang[§], Samuel L. Barrow[†], Anthony T. Iavarone[†‡], and Judith P. Klinman[†‡⊥*]

[†]Department of Chemistry, [‡]California Institute for Quantitative Biosciences, and [⊥]Department of Molecular and Cell Biology, University of California, Berkeley, Berkeley, California, 94720, United States.
§David R. Cheriton School of Computer Science, University of Waterloo, Waterloo, ON N2L 3G1, Canada
*To whom correspondence should be addressed, E-mail: klinman@berkeley.edu

This PDF file includes:

Figures S1 to S9

Tables S1 to S12

Legends for Datasets S1 to S2

SI References

Other supplementary materials for this manuscript include the following:

Datasets S1 to S2

Figures and Tables for the HDX-MS Analyses.

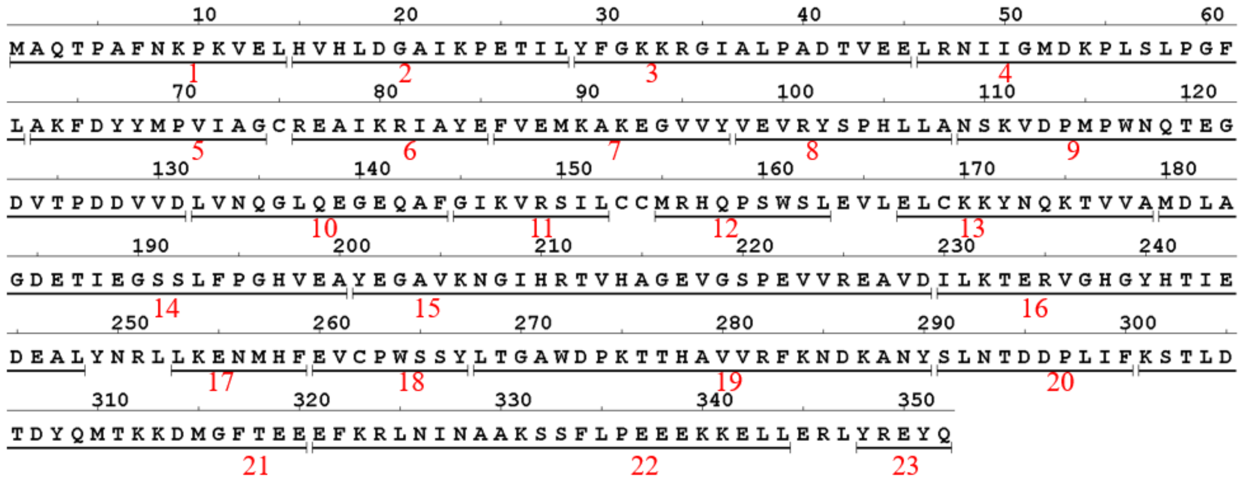

Figure S1. Protein sequence coverage map for wild type mADA with non-overlapping peptides 1-23 numbered.

Table S1. Non-overlapping peptide set for mADA.

start number	end number	Length of amino acid	Number of exchangeable amides (N_T)	Sequence
1	14	14	10	MAQTPAFNKPKVEL
15	28	14	11	HVHLDGAIKPETIL
29	45	17	14	YFGKKRGIALPADTVEE
46	62	17	13	LRNIIGMDKPLSLPGFL
63	74	12	9	AKFDYYMPVIAG
76	85	10	8	REAIKRIAYE
86	97	12	10	FVEMKAKEGVVY
98	108	11	8	VEVRYSPHLLA
109	131	23	18	NSKVDPMPWNQTEGDVTPDDVVD
132	144	13	11	LVNQGLQEGEQAF
145	152	8	6	GIKVRSIL
155	163	9	6	MRHQPSWSL
167	179	13	11	ELCKKYNQKTVVA
180	200	21	18	MDLAGDETIEGSSLFPGHVEA
201	229	29	26	YEGAVKNGIHRTVHAGEVGSPEVVREAVD
230	248	19	17	ILKTERVGHGYHTIEDEAL
253	259	7	5	LKENMHF
260	267	8	5	EVCPWSSY
268	290	23	20	LTGAWDPKTTHAVVRFKNDKANY
291	300	10	7	SLNTDDPLIF
301	320	20	18	KSTLDTDYQMTKKDMGFTEE
321	344	24	21	EFKRLNINAAKSSFLPEEEKKELL
348	352	5	3	YREYQ
Average length		14.739	11.957	

Table S2. Back exchange values (%) for each peptide of wild type mADA. Values for each peptide were averaged from three independent experiments.

Peptide	[a]Back exchange
1-14	20.255
15-28	41.235
29-45	51.385
46-62	38.38
63-74	61.425
76-85	24.245
86-97	56.18
98-108	34.27
109-131	45.38
132-144	38.915
145-152	18.125
155-163	46.215
167-179	33.615
180-200	43.64
201-229	63.365
230-248	67.895
253-259	52.505
260-267	31.66
268-290	64.82
291-300	48.01
301-320	29.35
321-344	26.195
348-352	2.88

[a]The average back exchange value is consistent with what we have observed in our lab (10, 11) .

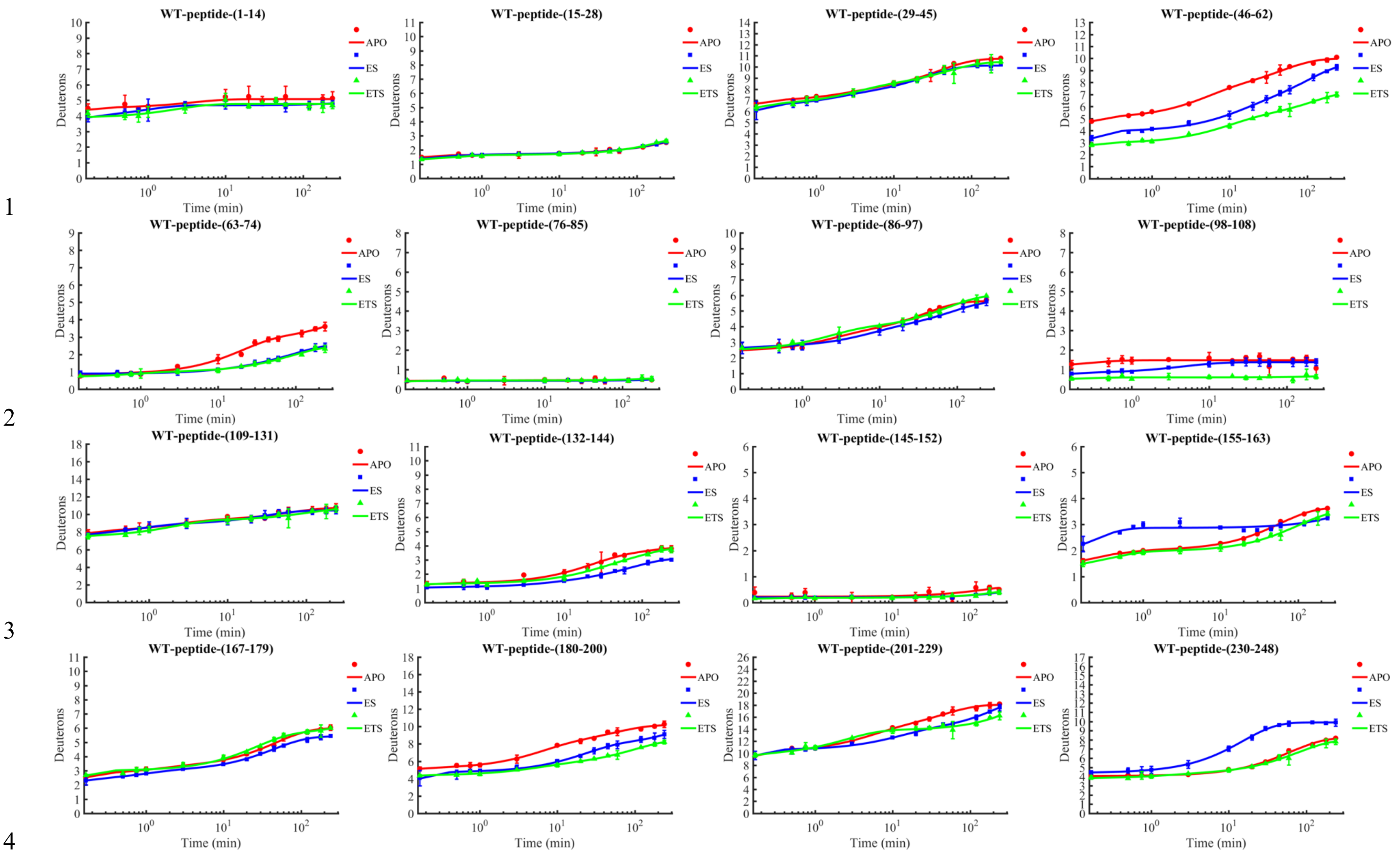

WT-peptide-(1-14)
WT-peptide-(15-28)
WT-peptide-(29-45)
WT-peptide-(46-62)
1
WT-peptide-(63-74)
WT-peptide-(76-85)
WT-peptide-(86-97)
WT-peptide-(98-108)
2
WT-peptide-(109-131)
WT-peptide-(132-144)
WT-peptide-(145-152)
WT-peptide-(155-163)
3
WT-peptide-(167-179)
WT-peptide-(180-200)
WT-peptide-(201-229)
WT-peptide-(230-248)
4
Deuterons
Time (min)
APO
ES
ETS

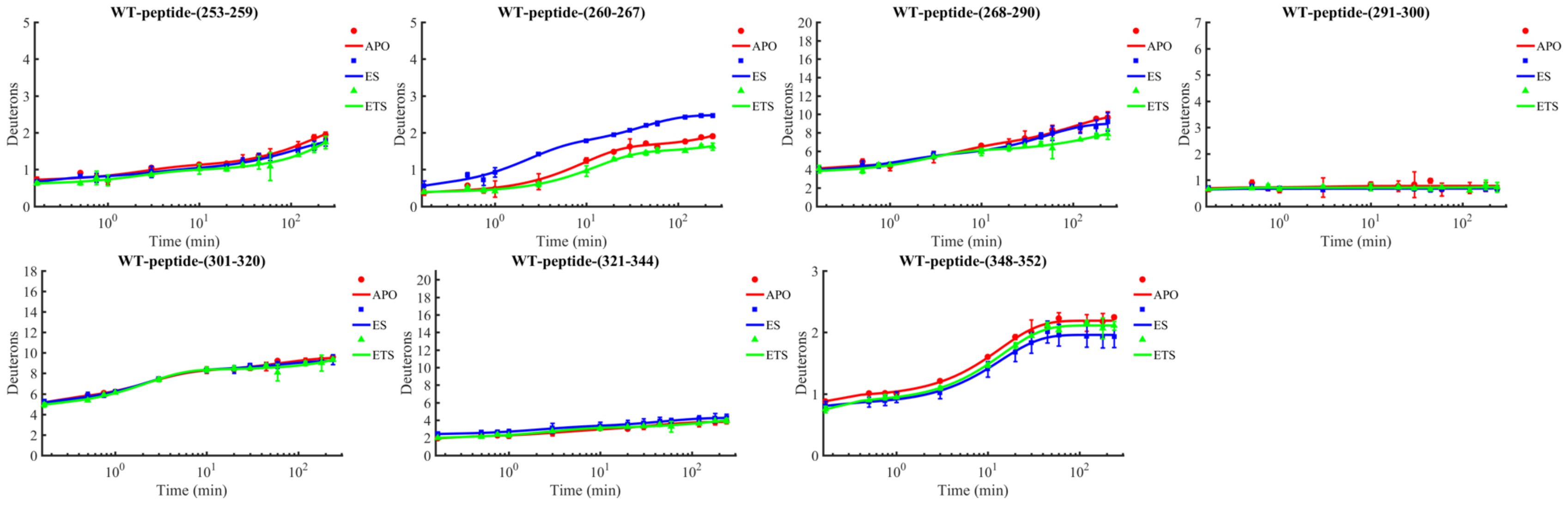

Figure S2. HDX traces comparison between substrate free (left), DAA bound (middle), and pentostatin (right) bound states for WT mADA at a single temperature 30 °C. HDX data was from three independent biological replicates.

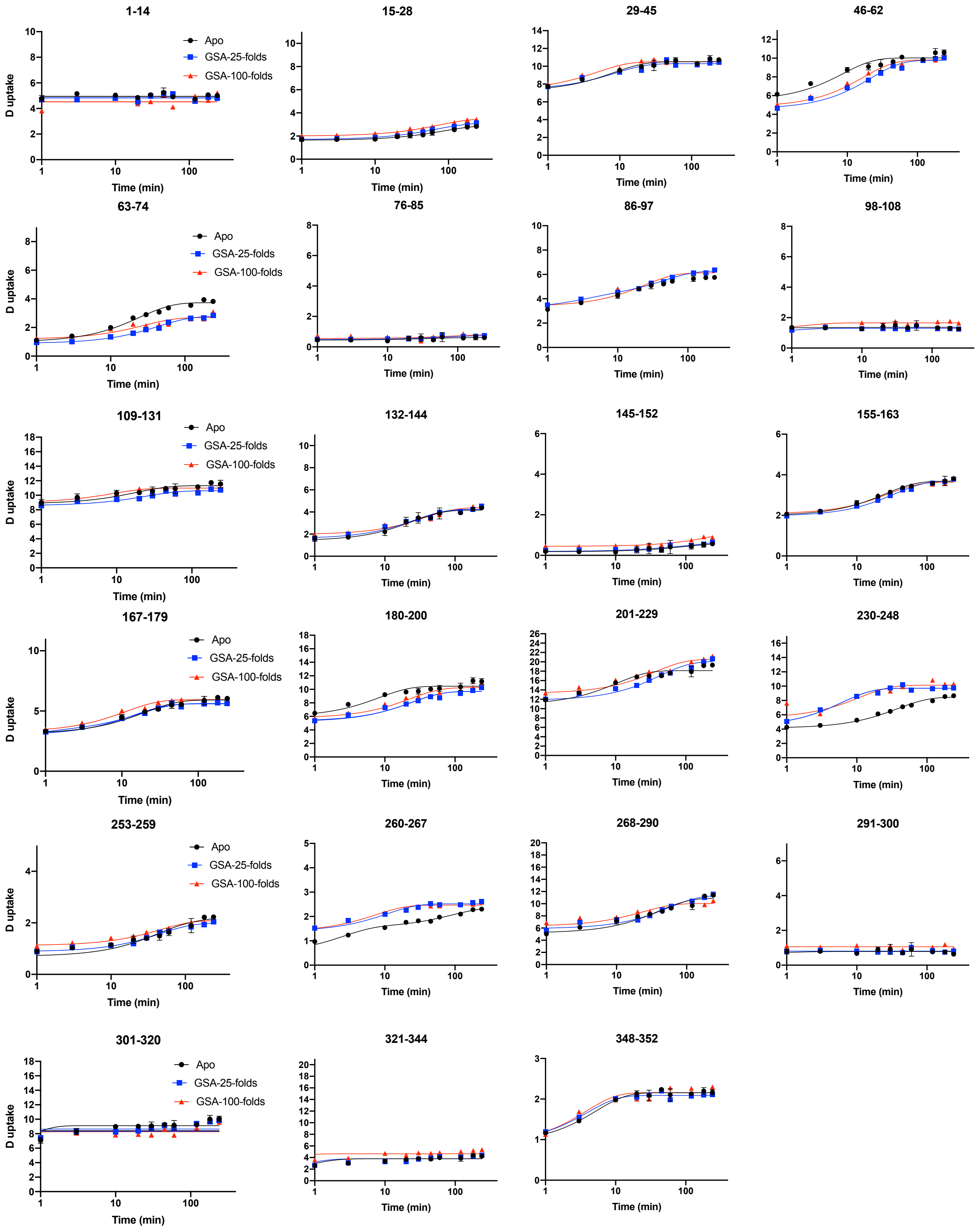
1-14
15-28
29-45
46-62
63-74
76-85
86-97
98-108
109-131
132-144
145-152
155-163
167-179
180-200
201-229
230-248
253-259
260-267
268-290
291-300
301-320
321-344
348-352
Apo
GSA-25-folds
GSA-100-folds
D uptake
Time (min)

Figure S3. Comparison of HDX traces of apo-, 25-fold DAA bound-, and 100-fold DAA bound-mADA at 40 ^{o}C. Slightly higher deuteron incorporation was observed for 100-fold DAA bound-mADA than for 25-fold DAA bound-mADA across most peptides, which is attributed to the fact that these two sets of experiments were conducted ~2-3 years apart with different preparations of proteins and different batches of analog. HDX data for apo form was from two biological replicates. HDX data for 25-fold DAA bound-, and 100-fold DAA bound-mADA was from one replicate.

Table S3. Distances (in Å) between active site residues and the active site zinc ion and its bound water/OH, and distances between active site residues and the bound analog in binary complexes. The distance relationships among Leu58, Phe61 and Phe65 are also summarized

	**His15 to Zn**	**His17 to Zn**	**His214 to Zn**	**Asp295 to Zn**	**His238 to Zn**	His 238 to Zn-H2O/OH	**Glu217 to Analog**	**Leu58 to analog**	**Phe61 to analog**	**Phe65 to analog**	**Leu58 to Phe61**	**Phe61 to Phe65**	**Gly184 to analog**	**Asp295 to analog**
Apo[a]	2.1	2.1	2.1	2.4	4.0	2.8					4.4	4.1		
DAA complex[b]	2.2	2.0	2.1	2.3	4.2	2.9<br>3.2 (H2O to C6)[c]	3.3	3.6	3.8	3.6/3.6	4.0	4.5	3.1	2.7
HDPR complex[d]	2.3	2.2	2.2	2.4	3.8	3.2	2.8	3.9	4.1	3.7/3.9	4.1	4.3	3.2	2.8
Pentostatin complex[e]	2.6	2.5	2.7	2.4	4.3	3.2	2.8	3.6	3.9	3.4/3.6	4.7	3.9	3.3	2.6

[a]PDB:3MVI (Chain A in PDB 3MVI, closed conformation)

[b]PDB:1ADD

[c]This distance is to C6 of bound DAA.

[d]PDB:2ADA

[e] PDB:1A4L

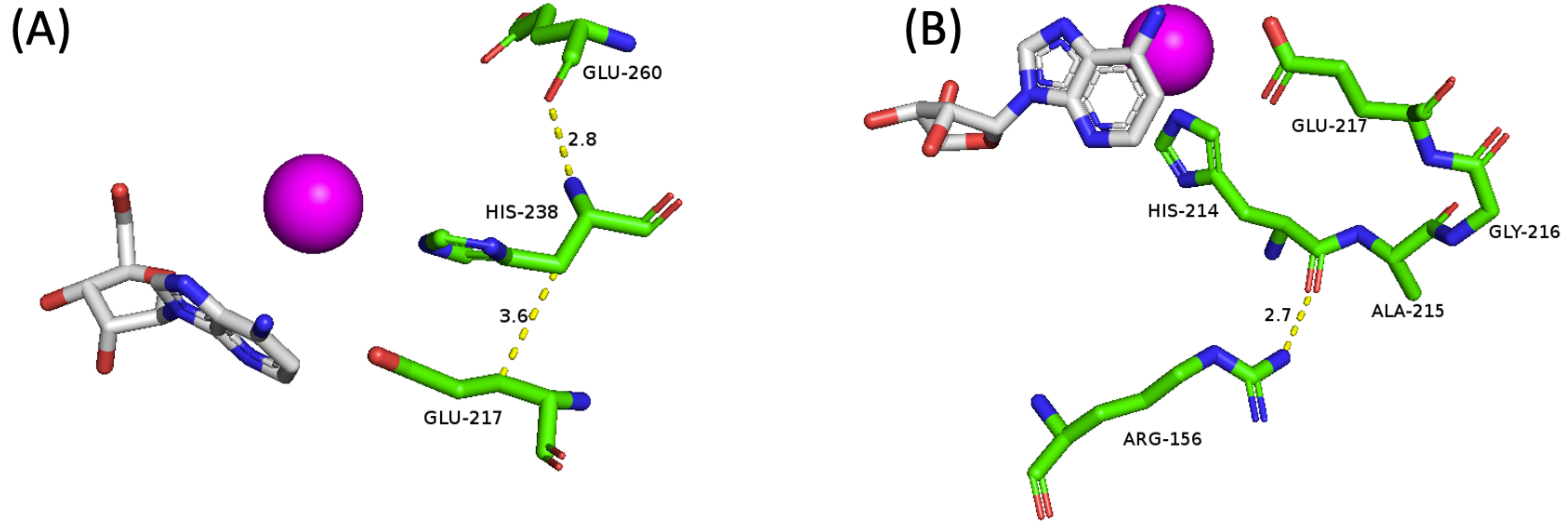

Figure S4. Interactions in regions of increased solvent accessibility upon binding of DAA. Residue Glu260 from peptide **260-267** is forming a backbone hydrogen bond with His238 from peptide **230-238** which is connecting with Glu217 via hydrophobic interaction (A), residue Arg156 from peptide **155-163** is backbone hydrogen bonding with a zinc ligand His215 that resides closely to Glu217 (B).

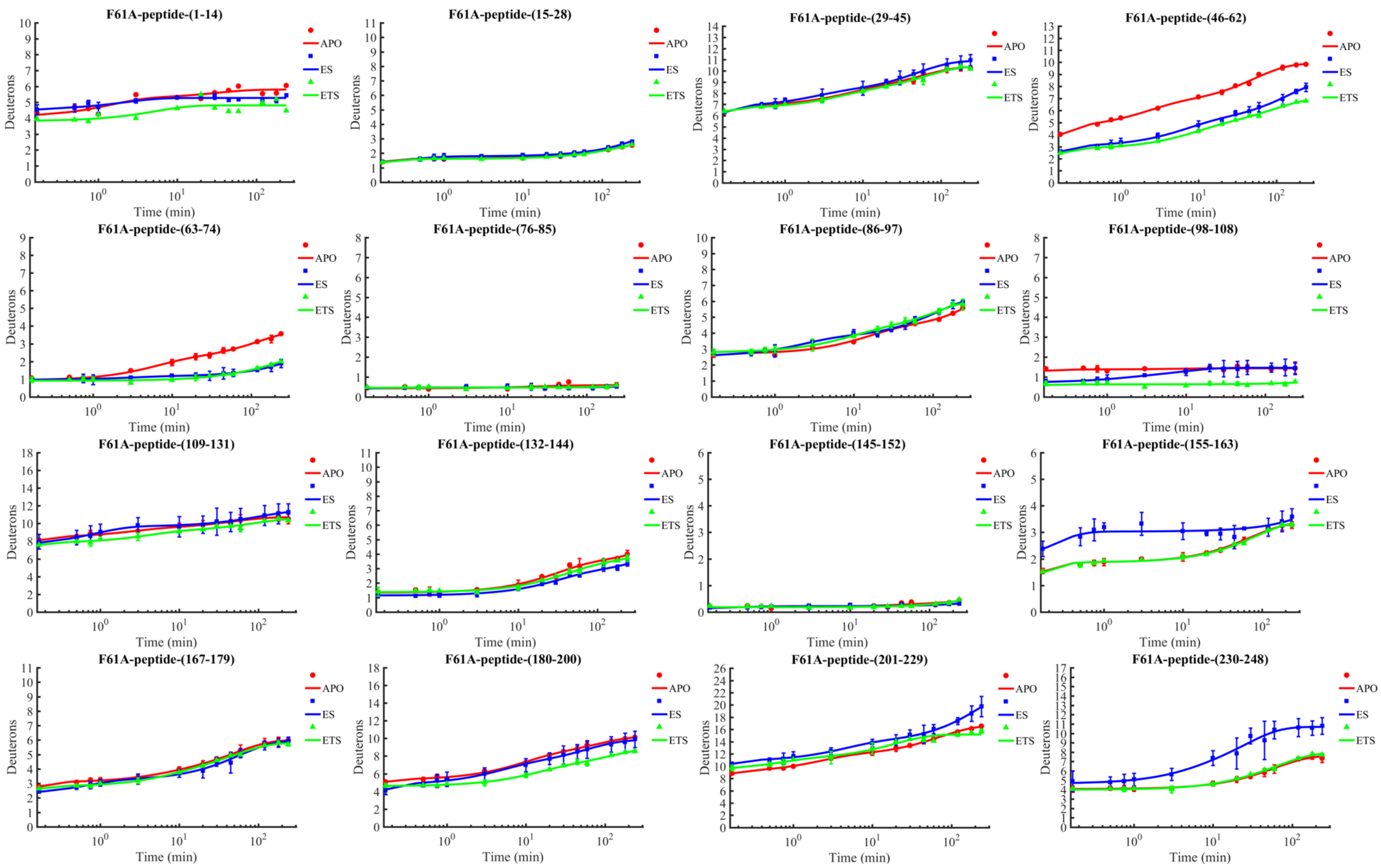
F61A-peptide-(1-14)
F61A-peptide-(15-28)
F61A-peptide-(29-45)
F61A-peptide-(46-62)
F61A-peptide-(63-74)
F61A-peptide-(76-85)
F61A-peptide-(86-97)
F61A-peptide-(98-108)
F61A-peptide-(109-131)
F61A-peptide-(132-144)
F61A-peptide-(145-152)
F61A-peptide-(155-163)
F61A-peptide-(167-179)
F61A-peptide-(180-200)
F61A-peptide-(201-229)
F61A-peptide-(230-248)
APO
ES
ETS
Deuterons
Time (min)

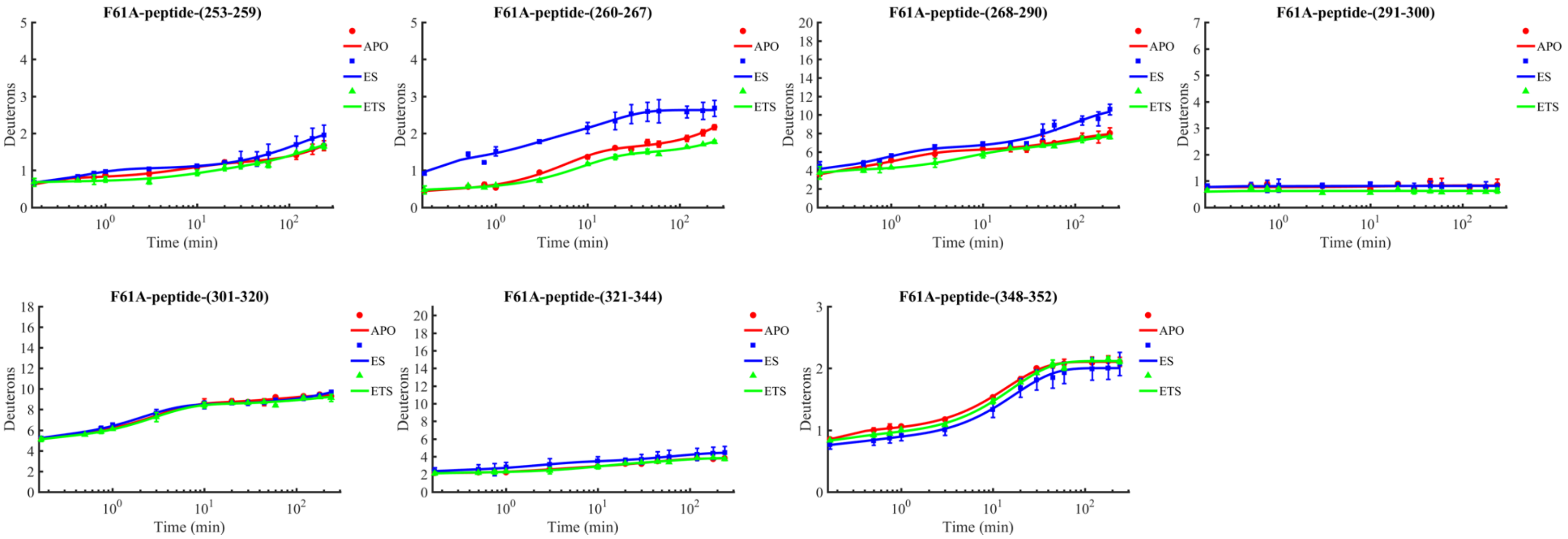

Figure S5. HDX traces comparison between substrate free (left), DAA bound (middle), and pentostatin (right) bound states for F61A mADA at a single temperature 30 °C. HDX data was from three independent biological replicates.

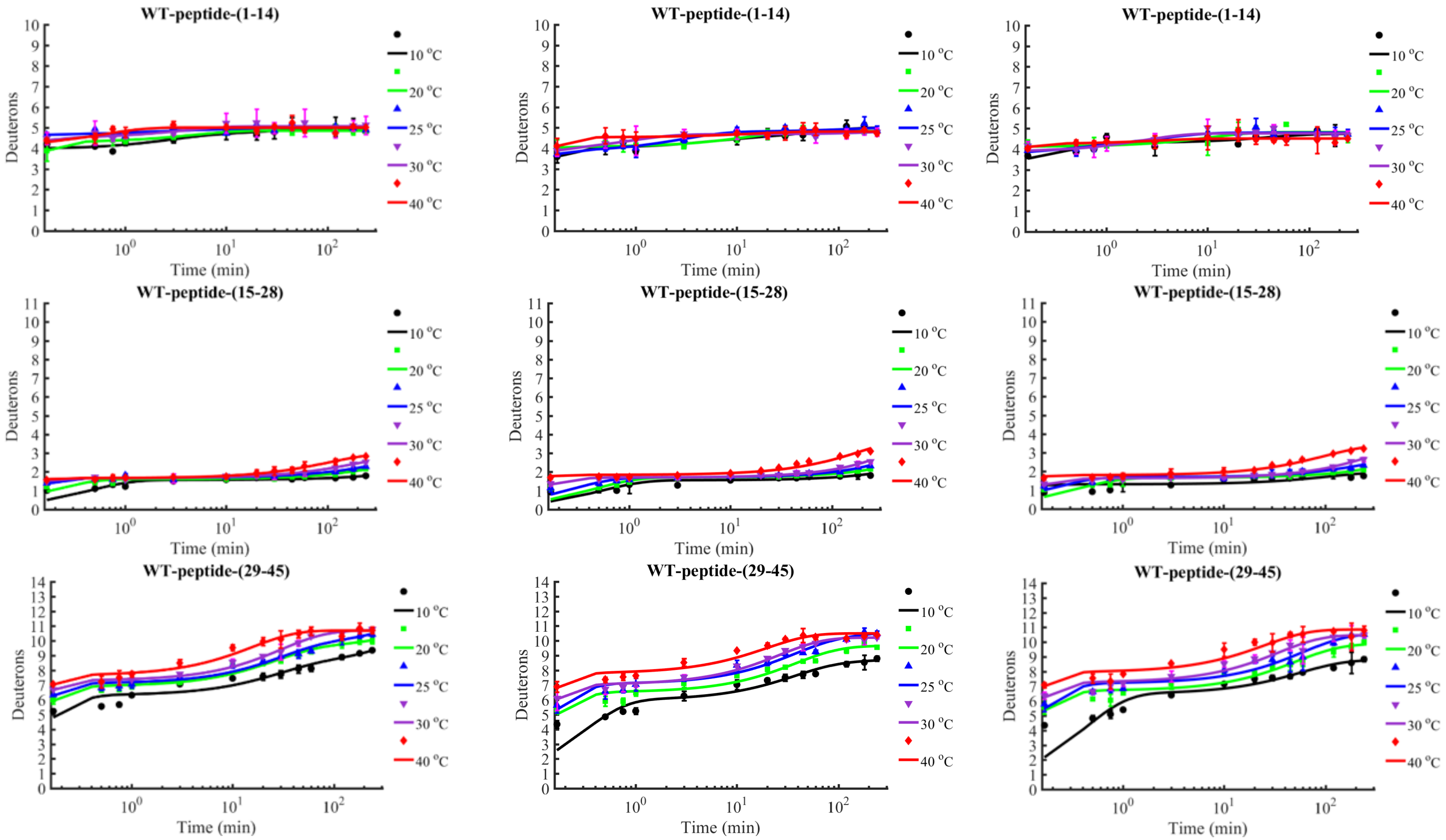
WT-peptide-(1-14)
WT-peptide-(1-14)
WT-peptide-(1-14)
WT-peptide-(15-28)
WT-peptide-(15-28)
WT-peptide-(15-28)
WT-peptide-(29-45)
WT-peptide-(29-45)
WT-peptide-(29-45)
Deuterons
Time (min)
10 °C
20 °C
25 °C
30 °C
40 °C

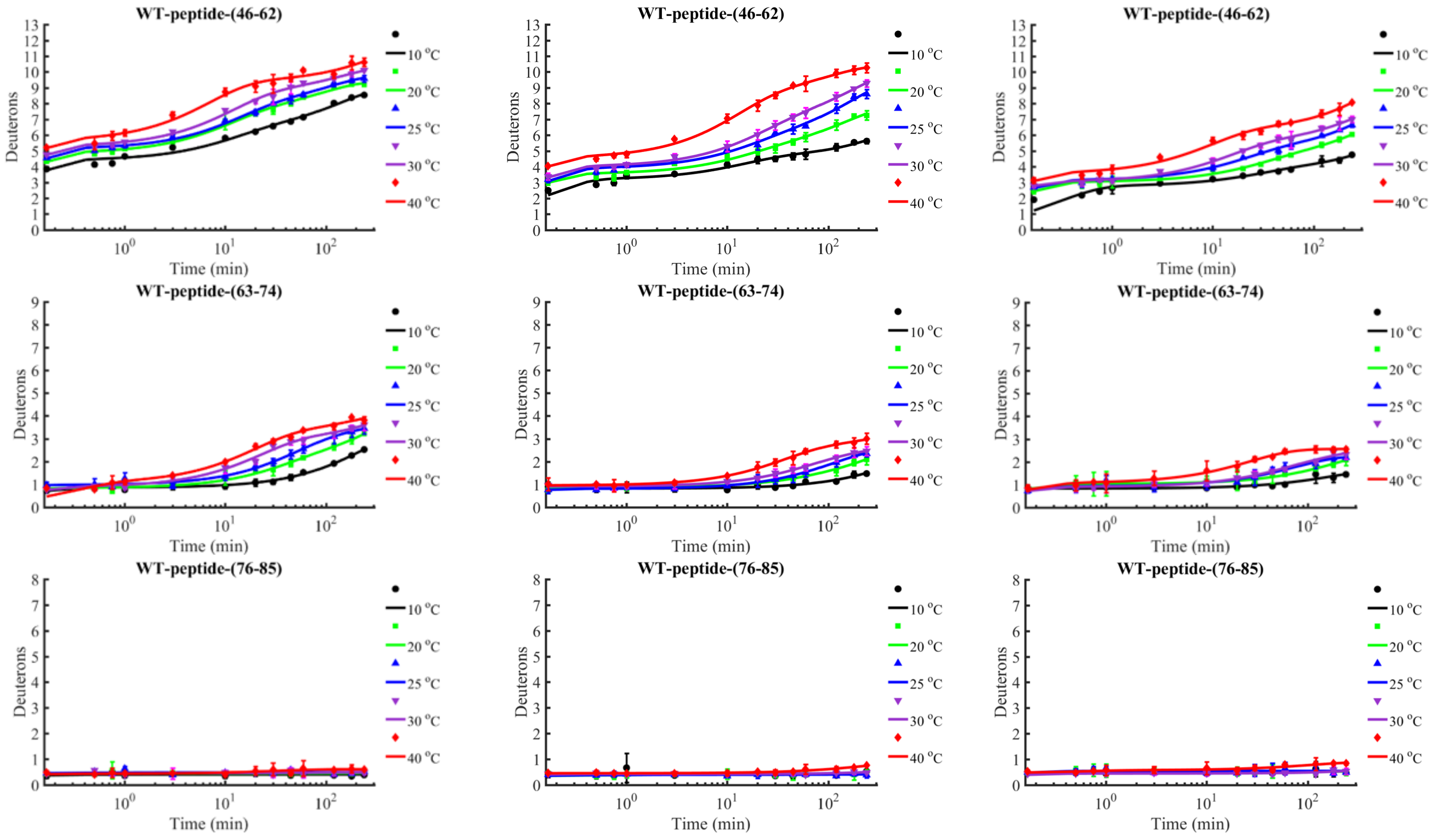
WT-peptide-(46-62)
Deuterons
Time (min)
10 °C
20 °C
25 °C
30 °C
40 °C
WT-peptide-(46-62)
Deuterons
Time (min)
WT-peptide-(46-62)
Deuterons
Time (min)
WT-peptide-(63-74)
Deuterons
Time (min)
WT-peptide-(63-74)
Deuterons
Time (min)
WT-peptide-(63-74)
Deuterons
Time (min)
WT-peptide-(76-85)
Deuterons
Time (min)
WT-peptide-(76-85)
Deuterons
Time (min)
WT-peptide-(76-85)
Deuterons
Time (min)

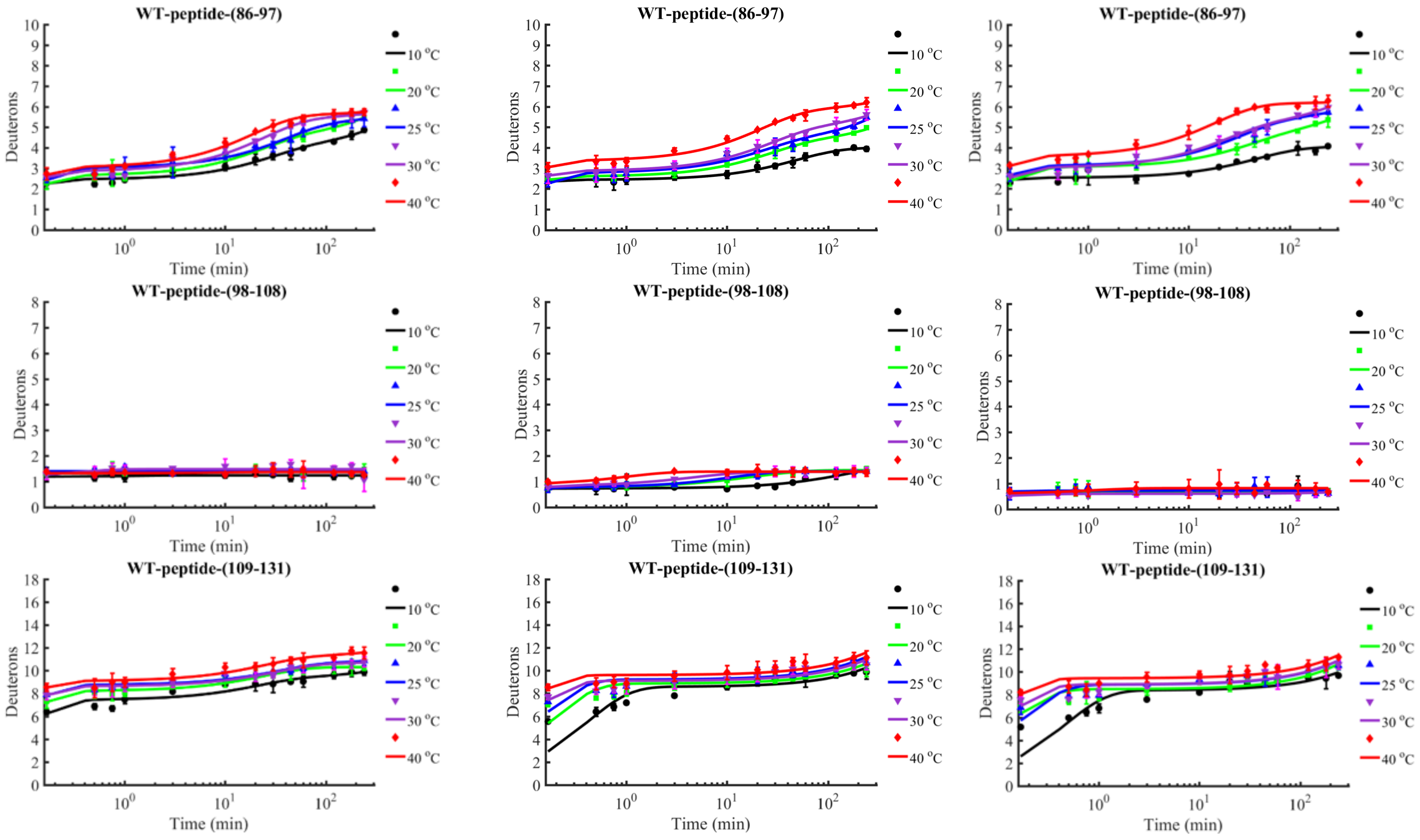
WT-peptide-(86-97)
WT-peptide-(86-97)
WT-peptide-(86-97)
WT-peptide-(98-108)
WT-peptide-(98-108)
WT-peptide-(98-108)
WT-peptide-(109-131)
WT-peptide-(109-131)
WT-peptide-(109-131)
Deuterons
Time (min)
10 °C
20 °C
25 °C
30 °C
40 °C

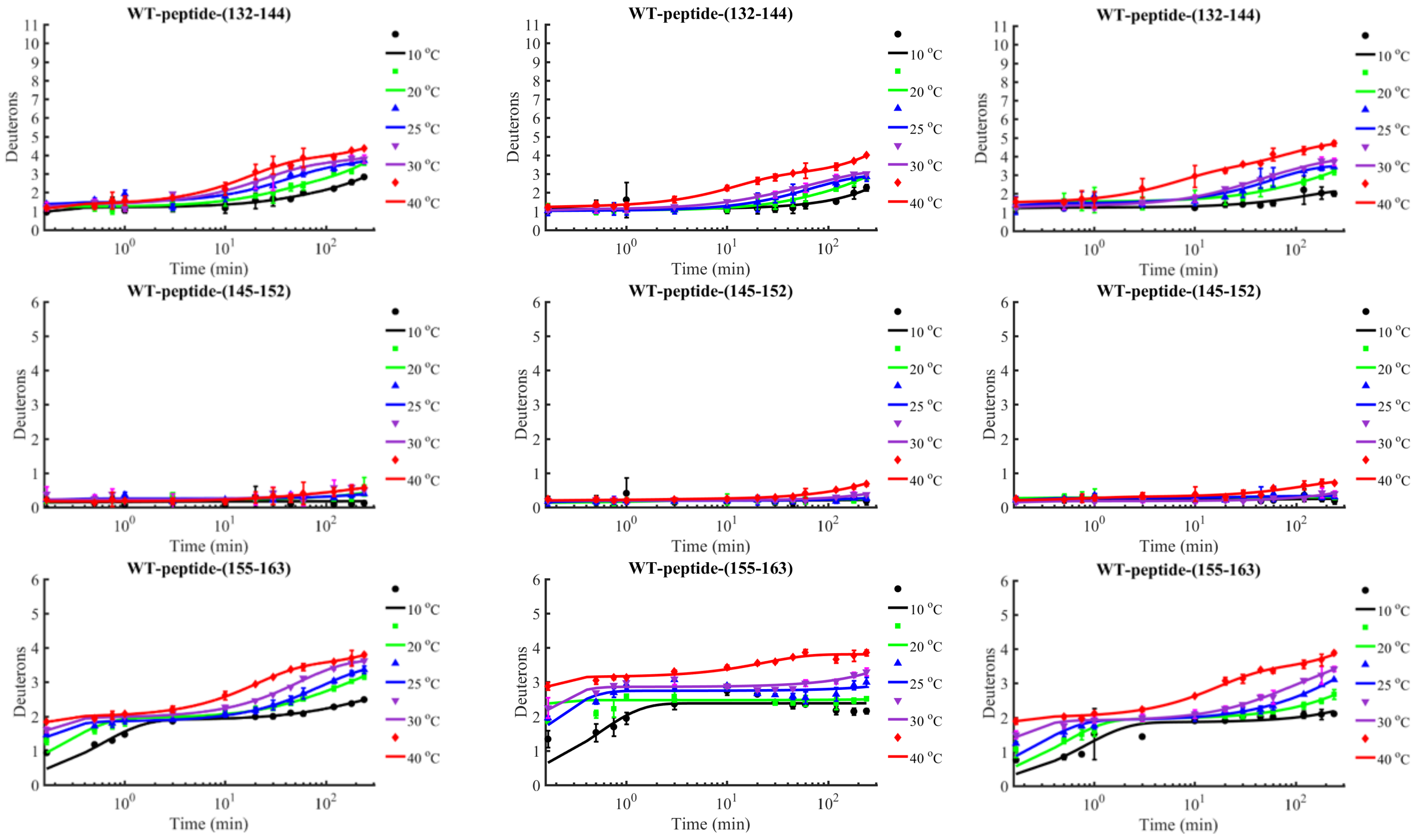
WT-peptide-(132-144)
WT-peptide-(145-152)
WT-peptide-(155-163)
Deuterons
Time (min)
10 °C
20 °C
25 °C
30 °C
40 °C

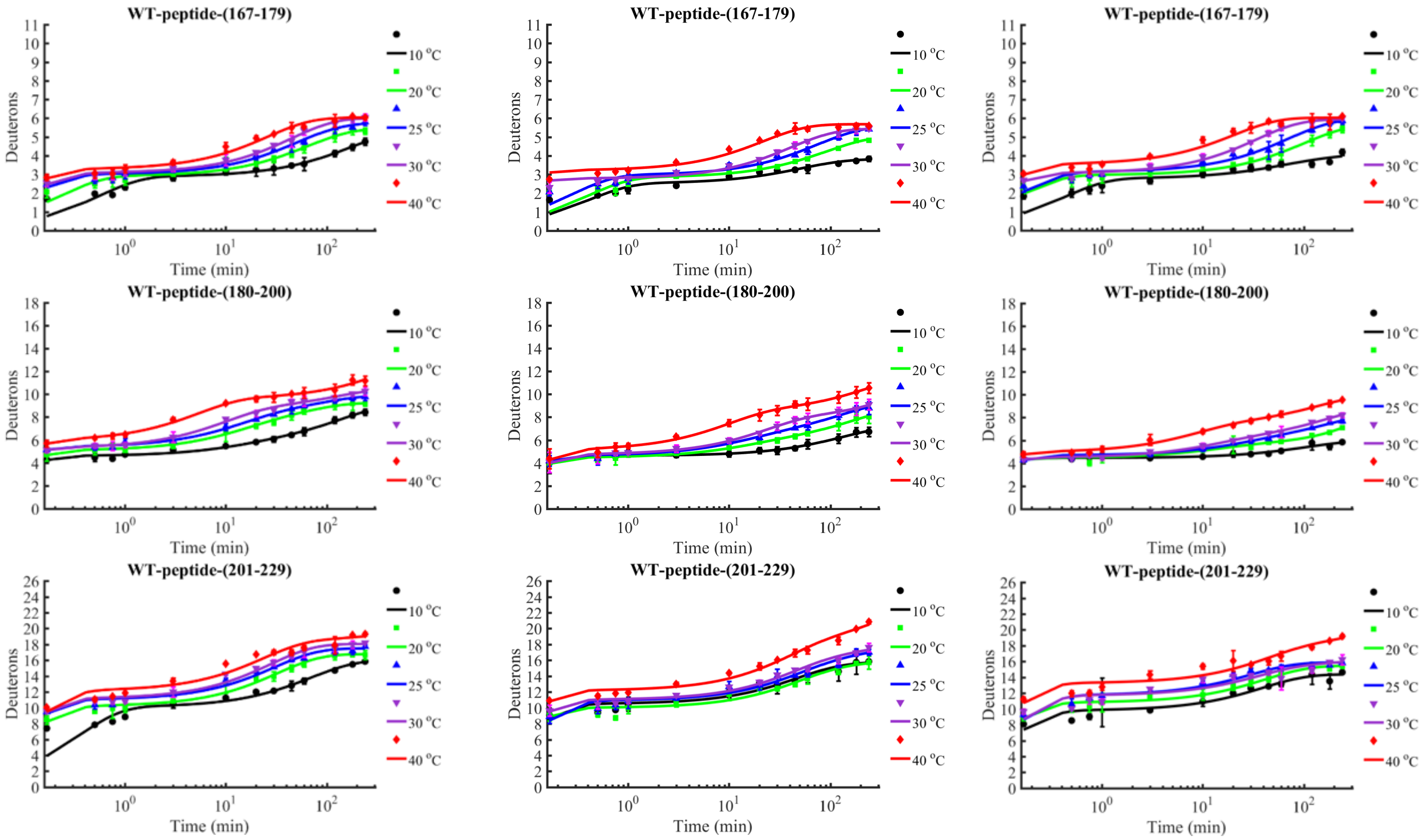
WT-peptide-(167-179)
WT-peptide-(167-179)
WT-peptide-(167-179)
WT-peptide-(180-200)
WT-peptide-(180-200)
WT-peptide-(180-200)
WT-peptide-(201-229)
WT-peptide-(201-229)
WT-peptide-(201-229)
Deuterons
Time (min)
10 °C
20 °C
25 °C
30 °C
40 °C

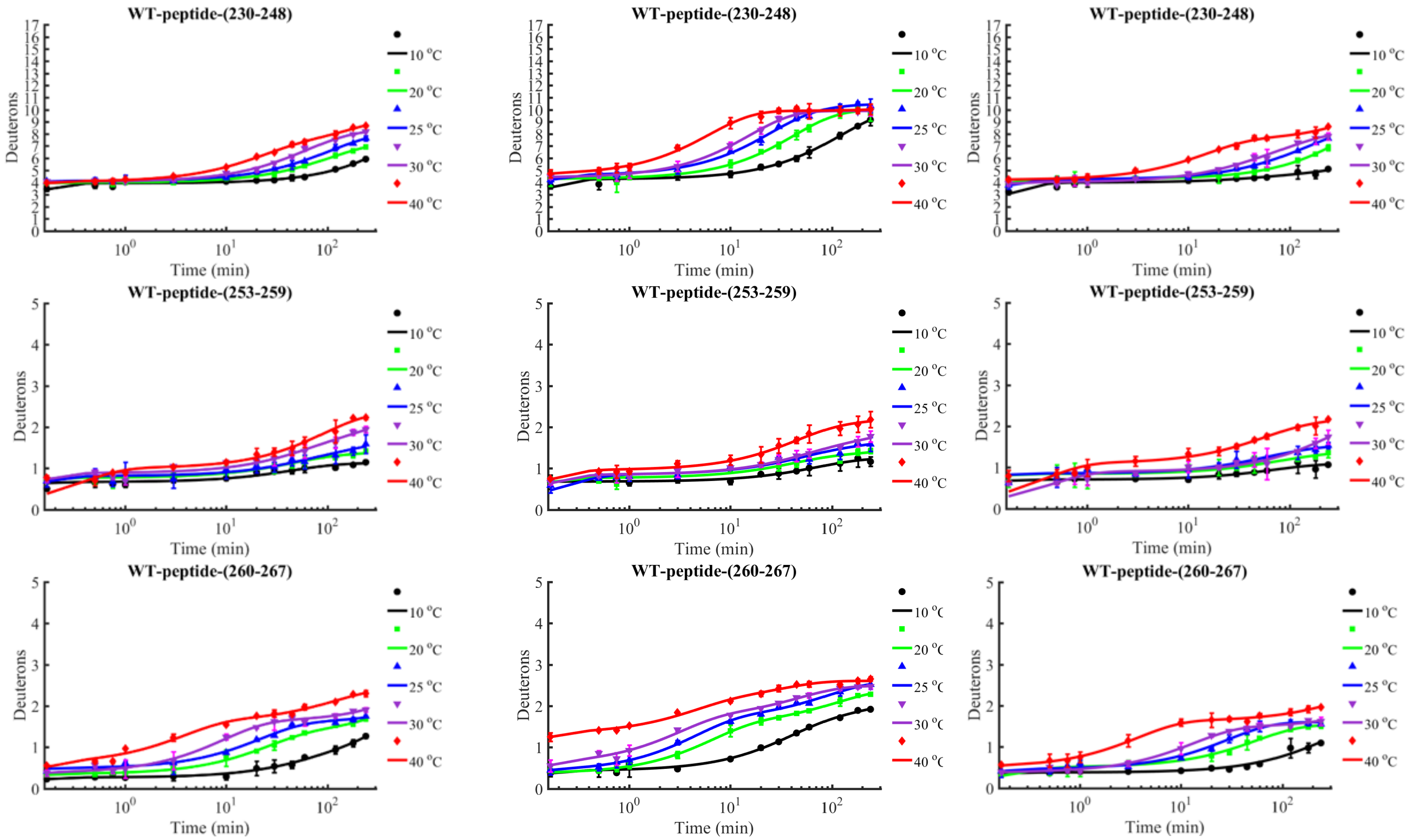
WT-peptide-(230-248)
WT-peptide-(253-259)
WT-peptide-(260-267)
Deuterons
Time (min)
10 °C
20 °C
25 °C
30 °C
40 °C

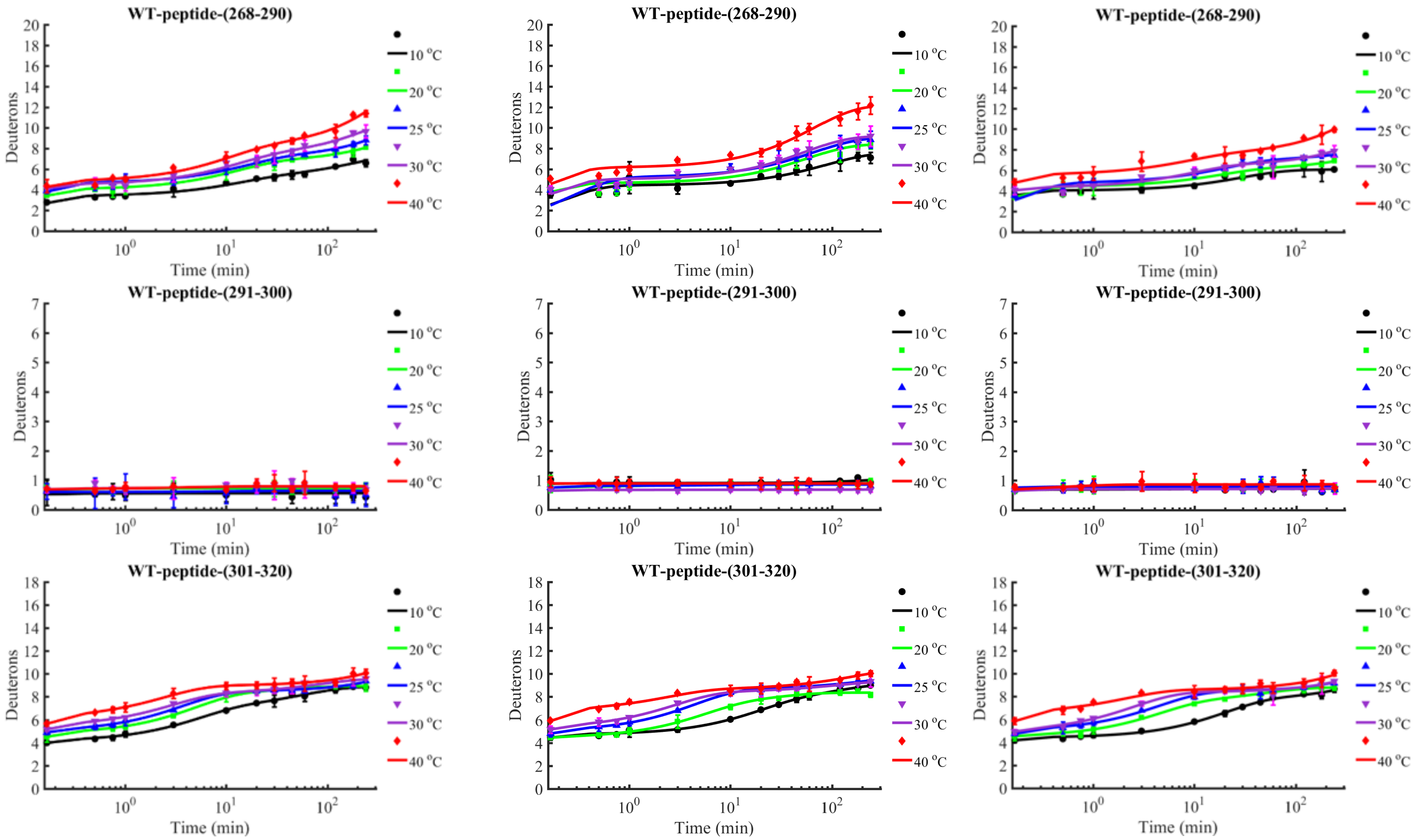
WT-peptide-(268-290)
WT-peptide-(268-290)
WT-peptide-(268-290)
WT-peptide-(291-300)
WT-peptide-(291-300)
WT-peptide-(291-300)
WT-peptide-(301-320)
WT-peptide-(301-320)
WT-peptide-(301-320)
Deuterons
Time (min)
10 °C
20 °C
25 °C
30 °C
40 °C

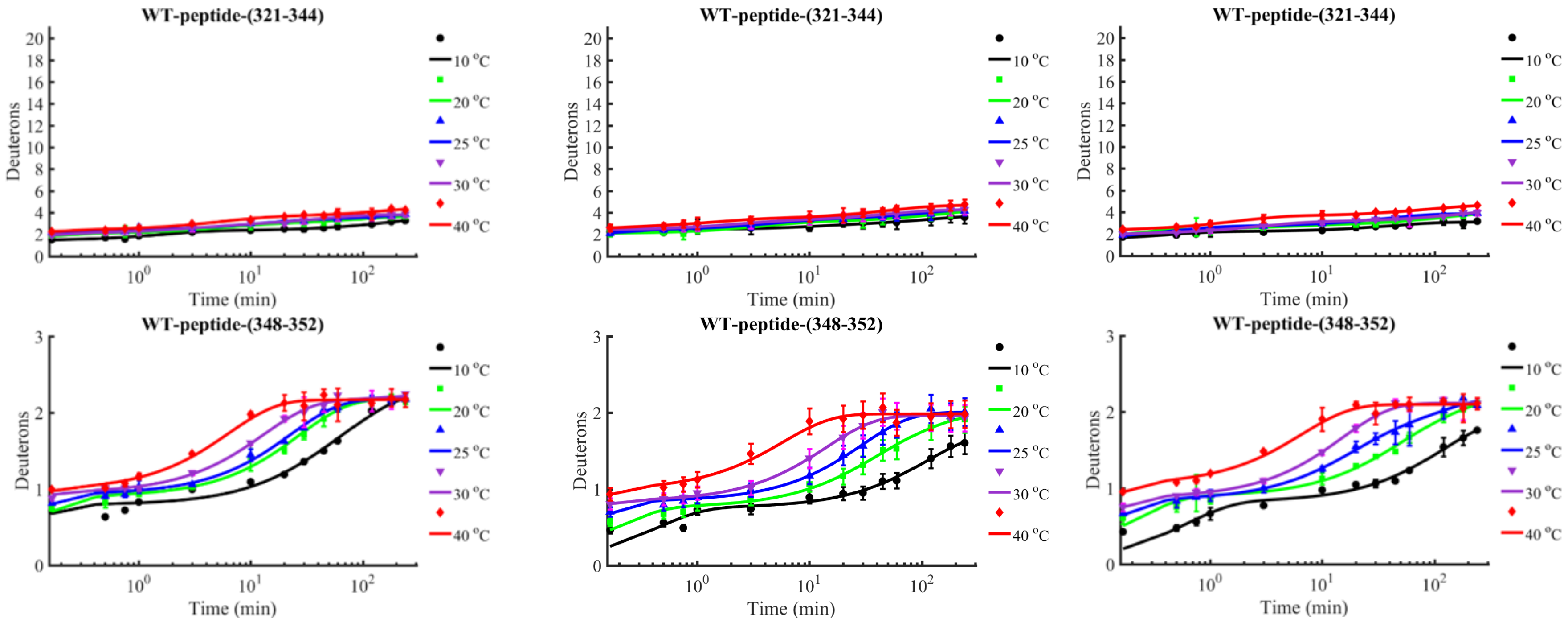

Figure S6. Experimental HDX traces as a function of temperature for substrate free (left), DAA bound (middle), and pentostatin (right) bound to WT mADA. HDX data was from two biological replicates.

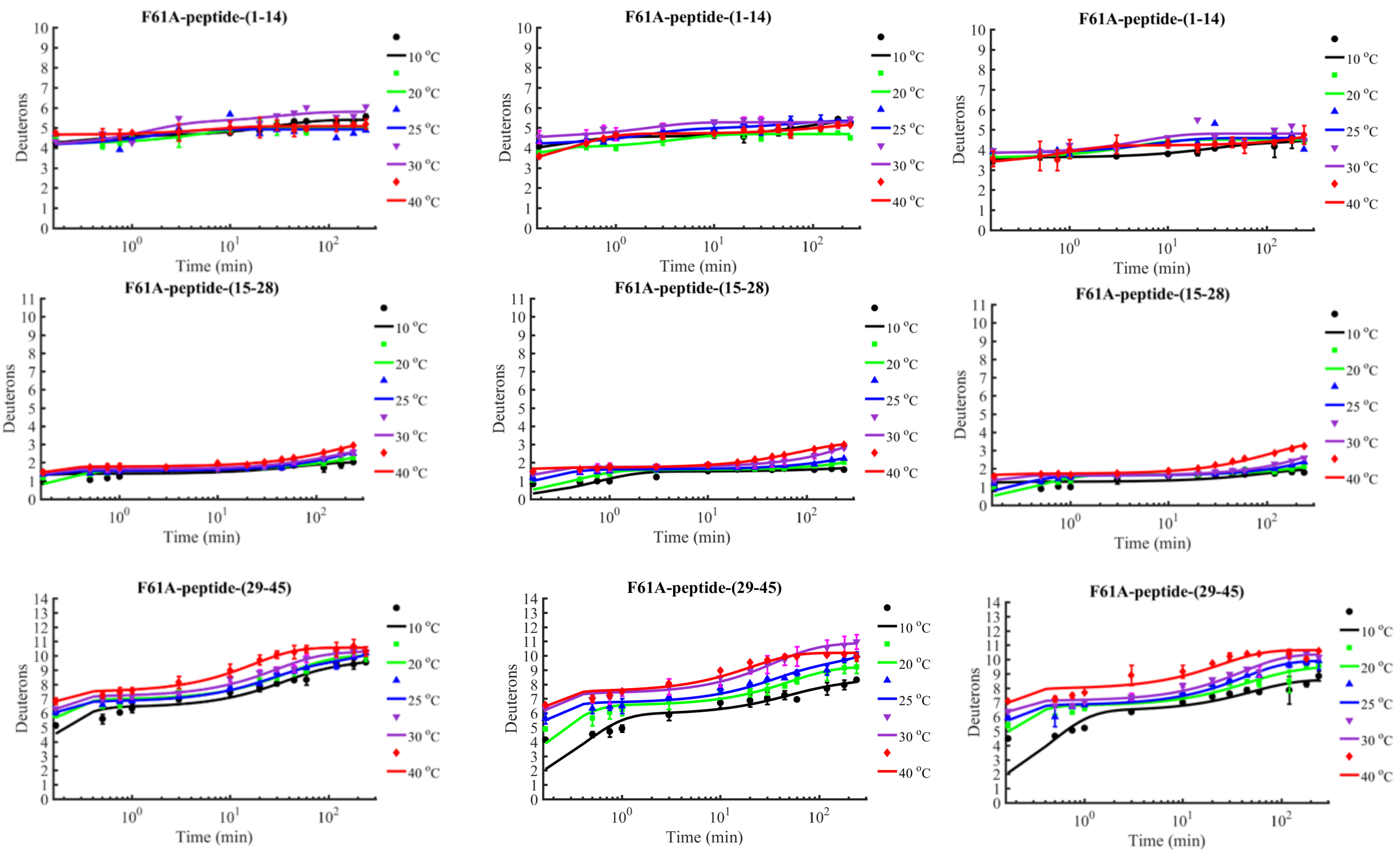
F61A-peptide-(1-14)
F61A-peptide-(15-28)
F61A-peptide-(29-45)
Deuterons
Time (min)
10 °C
20 °C
25 °C
30 °C
40 °C

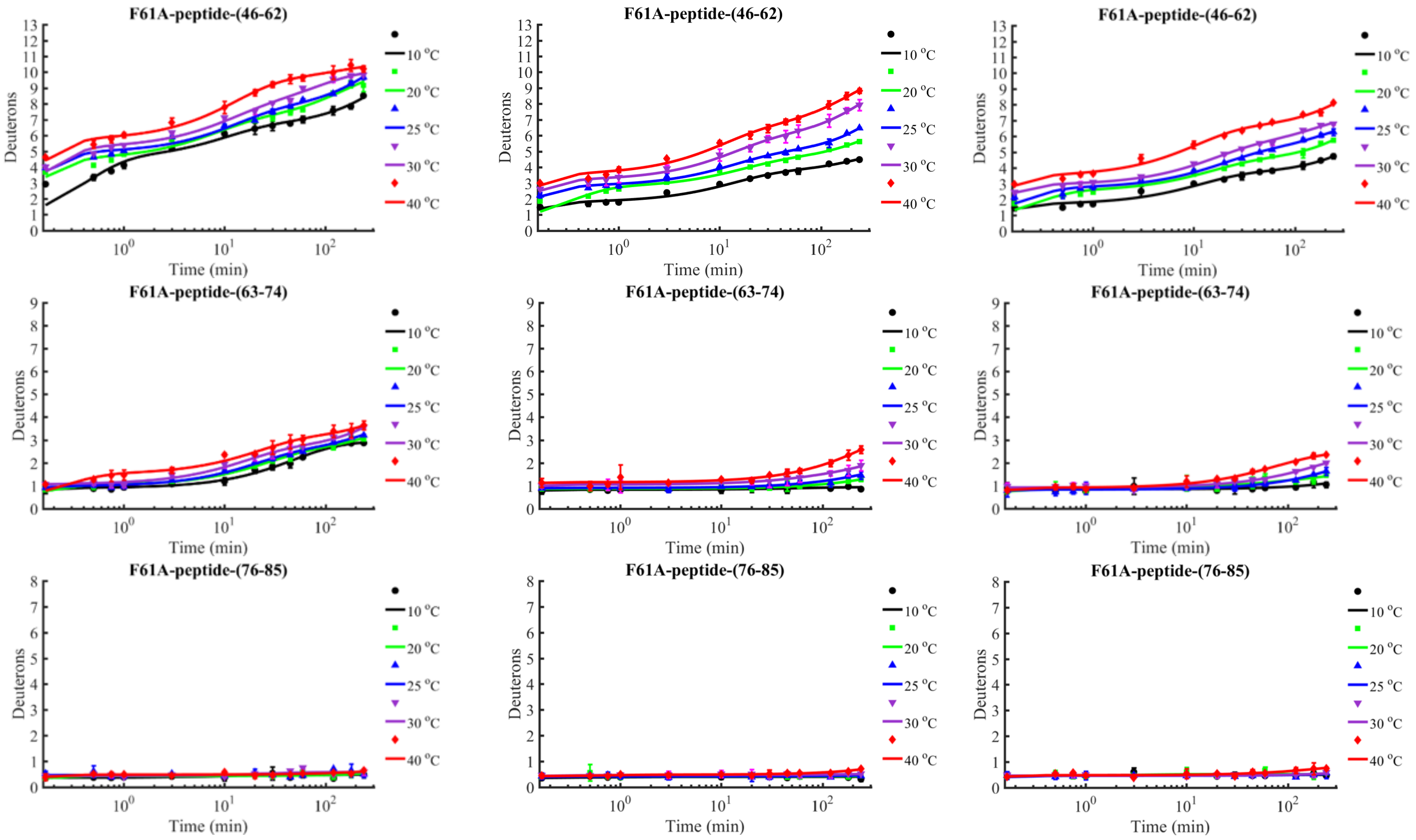
F61A-peptide-(46-62)
F61A-peptide-(46-62)
F61A-peptide-(46-62)
F61A-peptide-(63-74)
F61A-peptide-(63-74)
F61A-peptide-(63-74)
F61A-peptide-(76-85)
F61A-peptide-(76-85)
F61A-peptide-(76-85)
Deuterons
Time (min)
10 °C
20 °C
25 °C
30 °C
40 °C

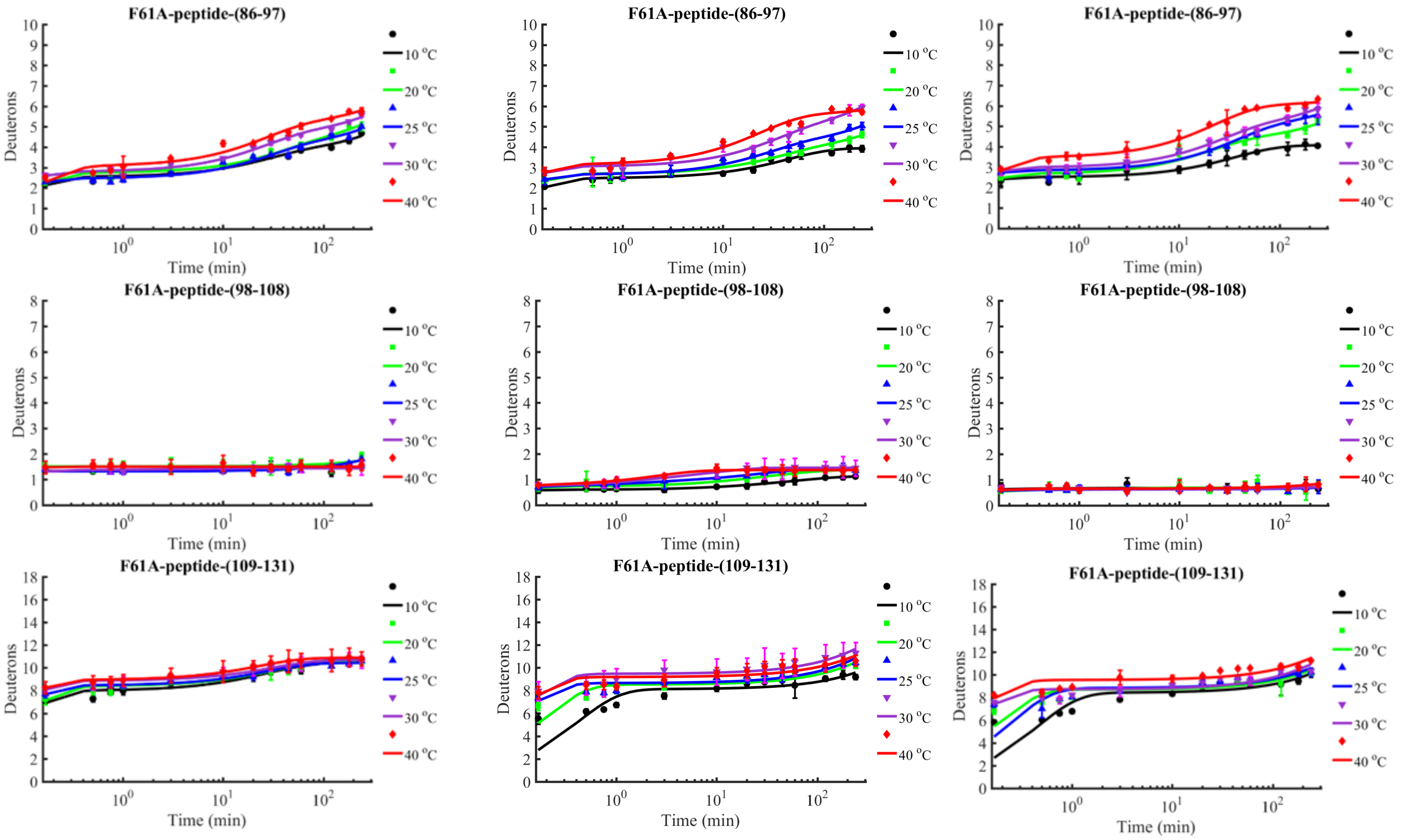
F61A-peptide-(86-97)
F61A-peptide-(86-97)
F61A-peptide-(86-97)
F61A-peptide-(98-108)
F61A-peptide-(98-108)
F61A-peptide-(98-108)
F61A-peptide-(109-131)
F61A-peptide-(109-131)
F61A-peptide-(109-131)
Deuterons
Time (min)
10 °C
20 °C
25 °C
30 °C
40 °C

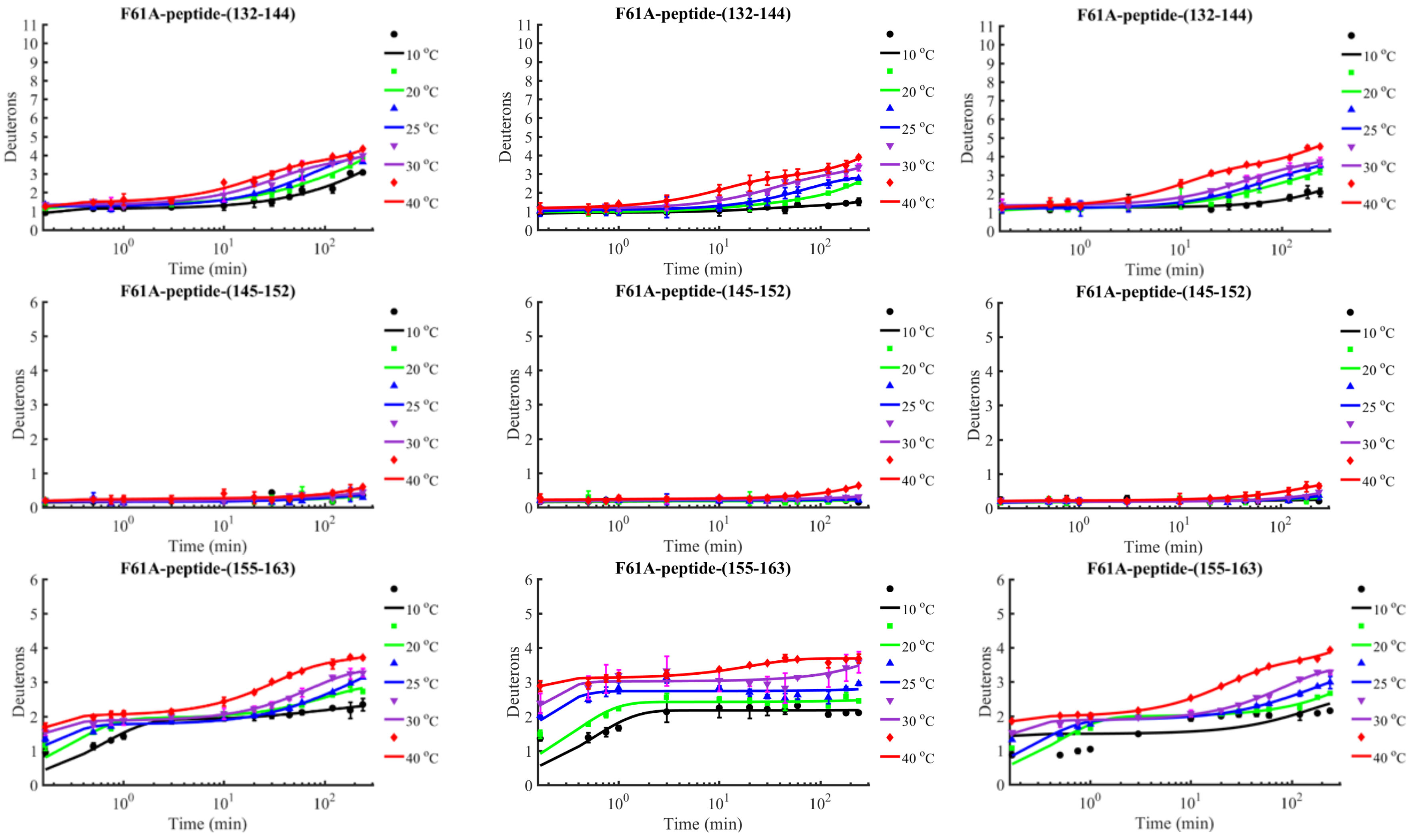
F61A-peptide-(132-144)
F61A-peptide-(132-144)
F61A-peptide-(132-144)
F61A-peptide-(145-152)
F61A-peptide-(145-152)
F61A-peptide-(145-152)
F61A-peptide-(155-163)
F61A-peptide-(155-163)
F61A-peptide-(155-163)
Deuterons
Time (min)
10 °C
20 °C
25 °C
30 °C
40 °C

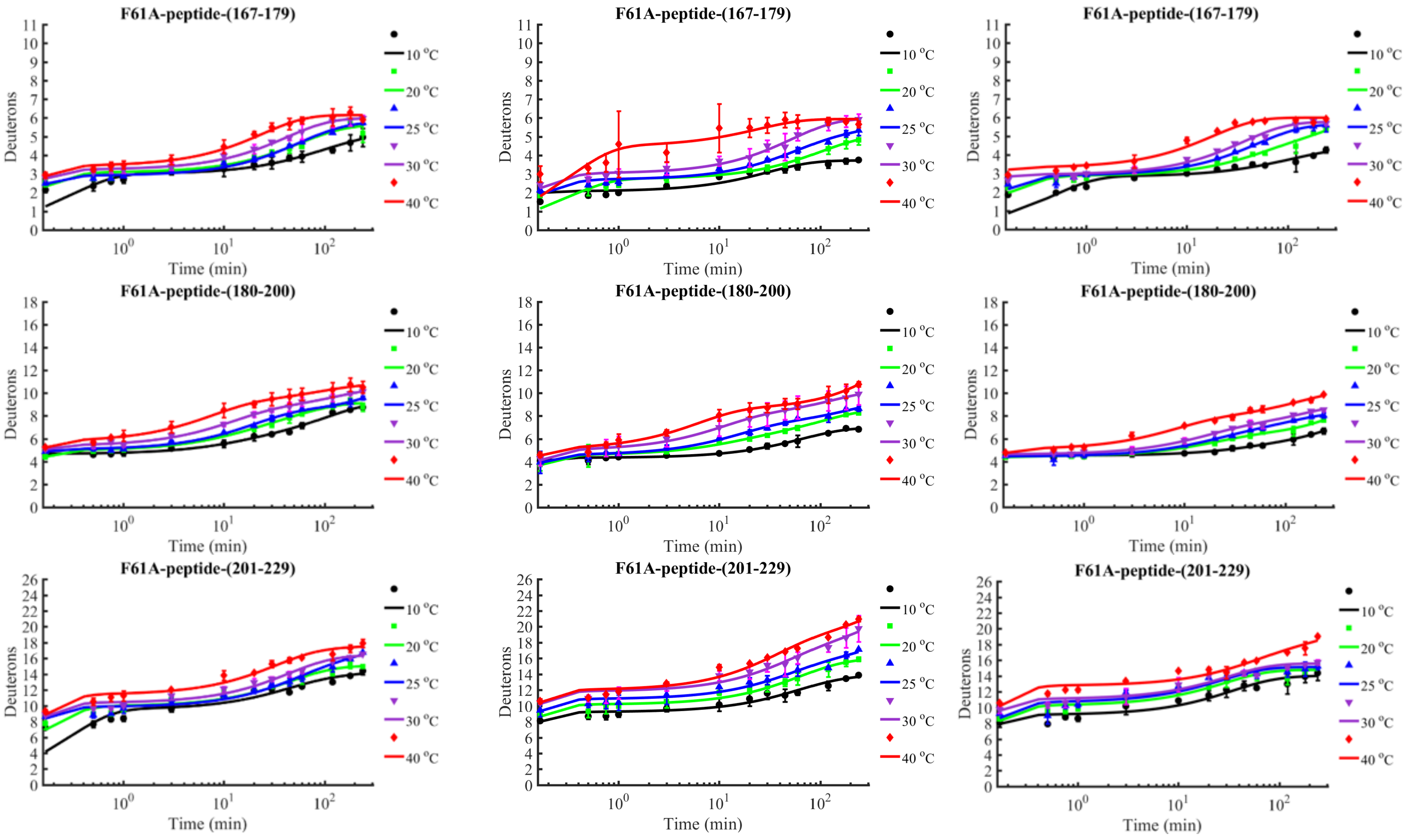
F61A-peptide-(167-179)
F61A-peptide-(180-200)
F61A-peptide-(201-229)
Deuterons
Time (min)
10 °C
20 °C
25 °C
30 °C
40 °C

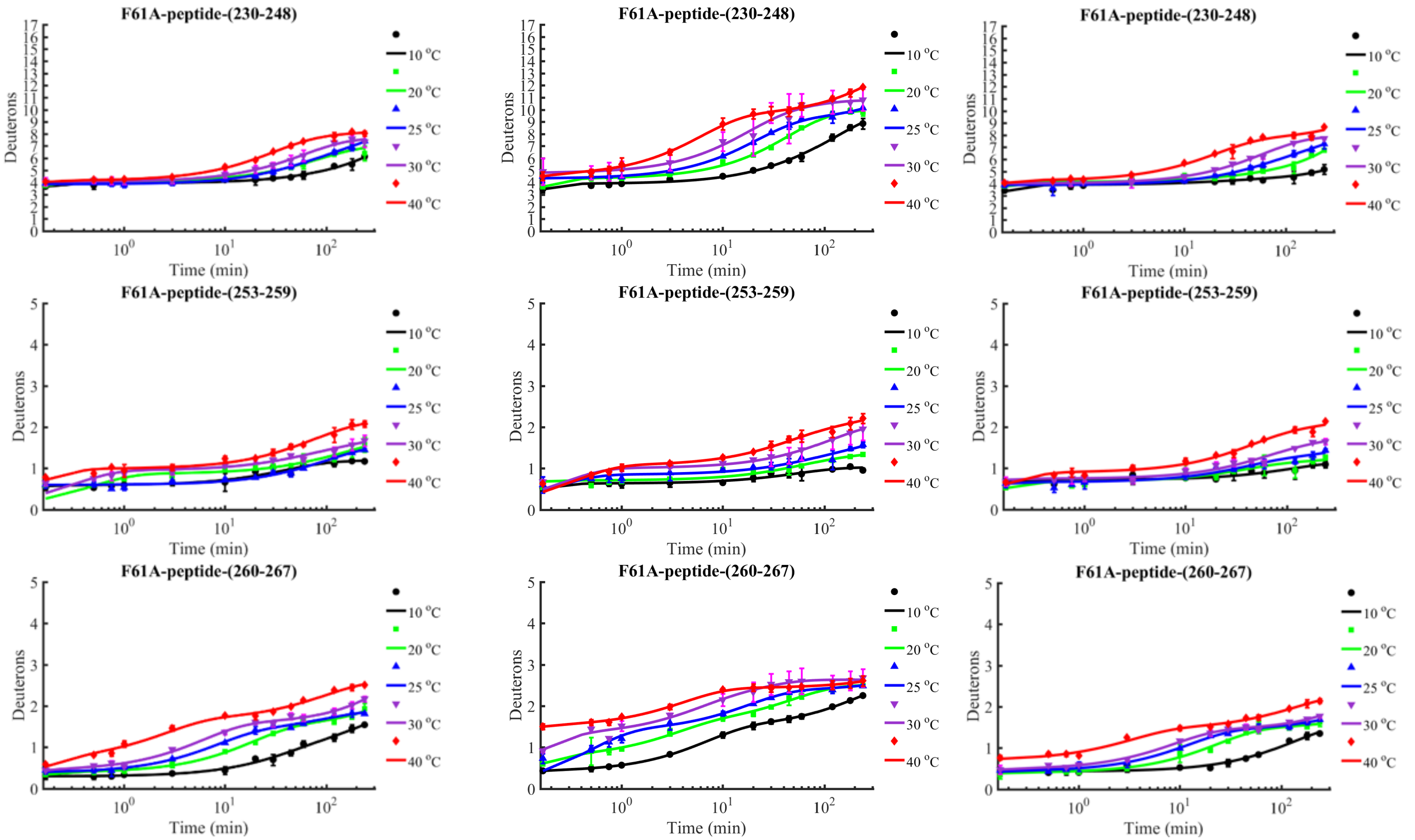
F61A-peptide-(230-248)
F61A-peptide-(230-248)
F61A-peptide-(230-248)
F61A-peptide-(253-259)
F61A-peptide-(253-259)
F61A-peptide-(253-259)
F61A-peptide-(260-267)
F61A-peptide-(260-267)
F61A-peptide-(260-267)
Deuterons
Time (min)
10 °C
20 °C
25 °C
30 °C
40 °C

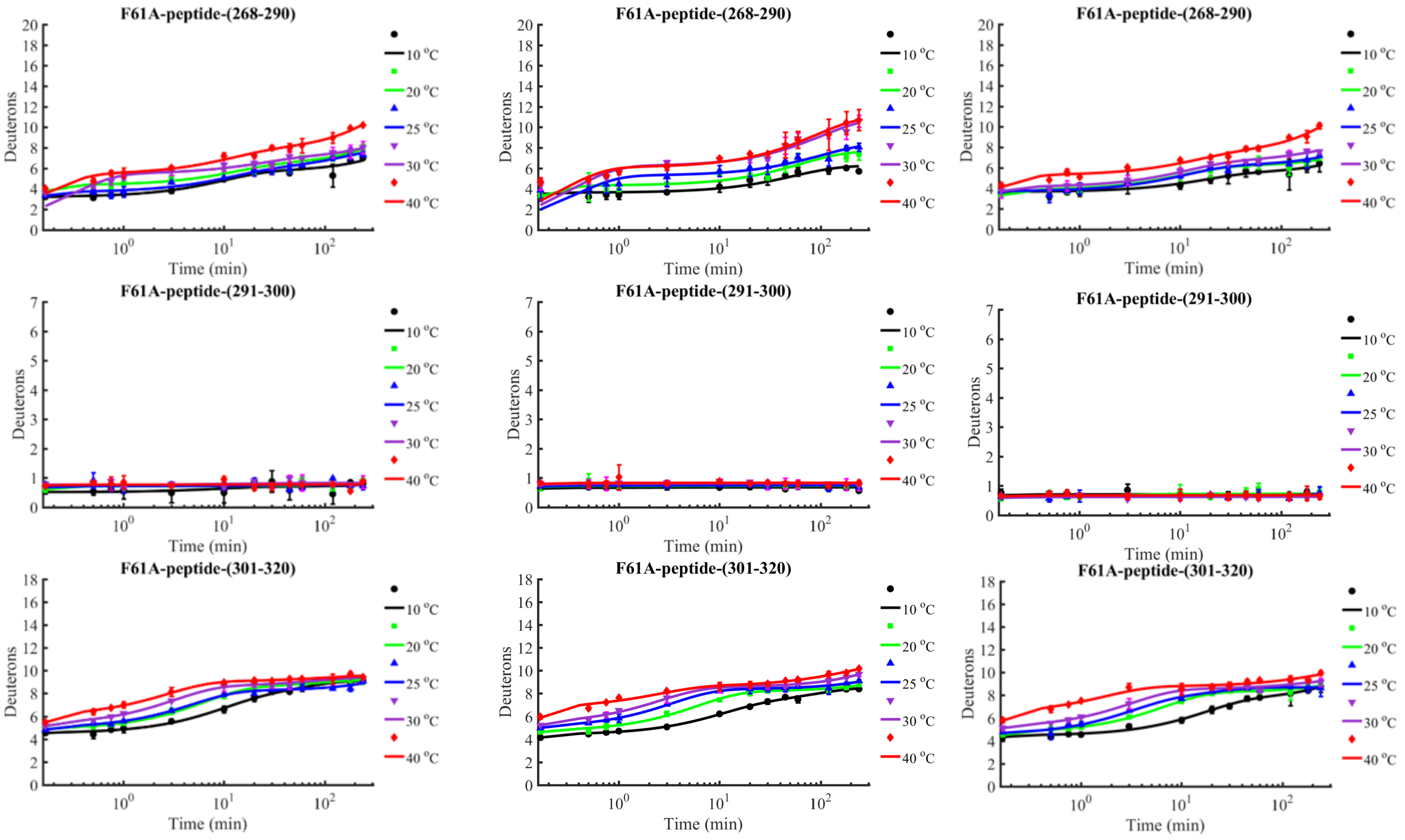
F61A-peptide-(268-290)
F61A-peptide-(291-300)
F61A-peptide-(301-320)
Deuterons
Time (min)
10 °C
20 °C
25 °C
30 °C
40 °C

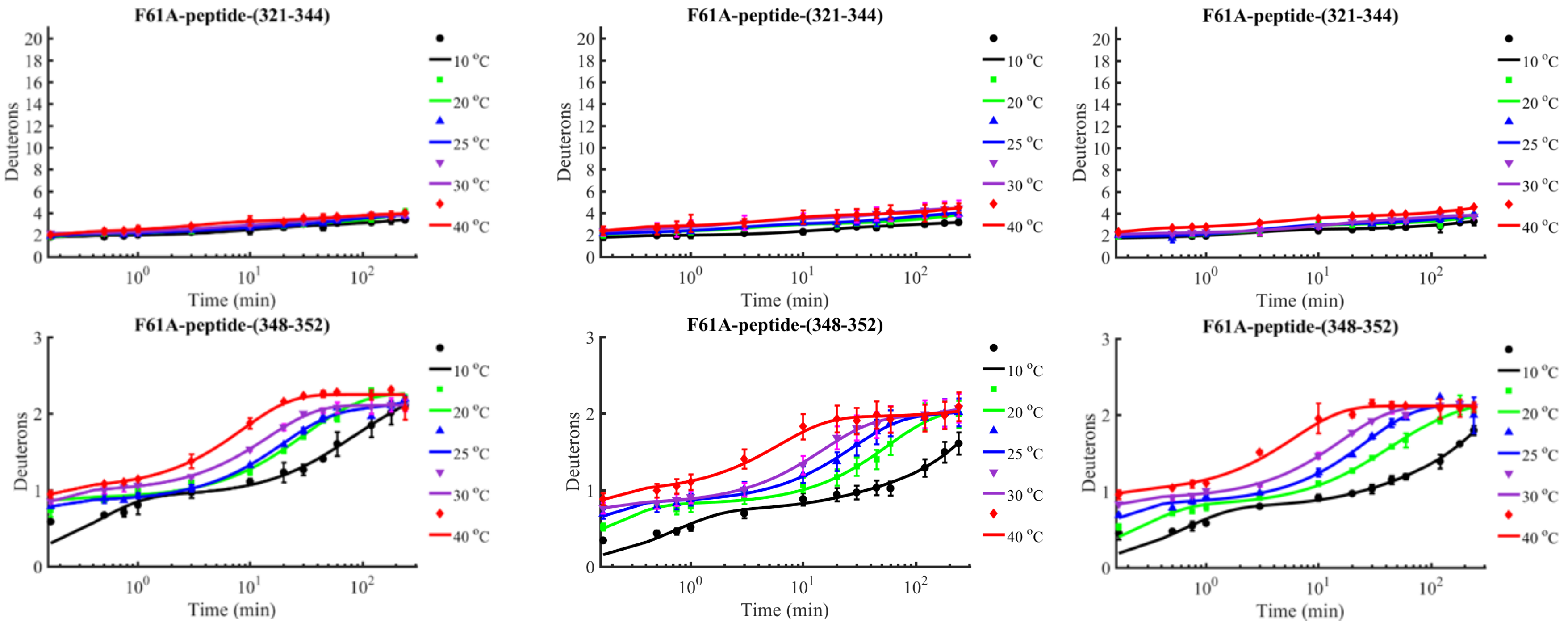

Figure S7. Experimental HDX traces as a function of temperature for substrate free (left), DAA bound (middle), and pentostatin (right) bound to F61A mADA. HDX data was from two biological replicates.

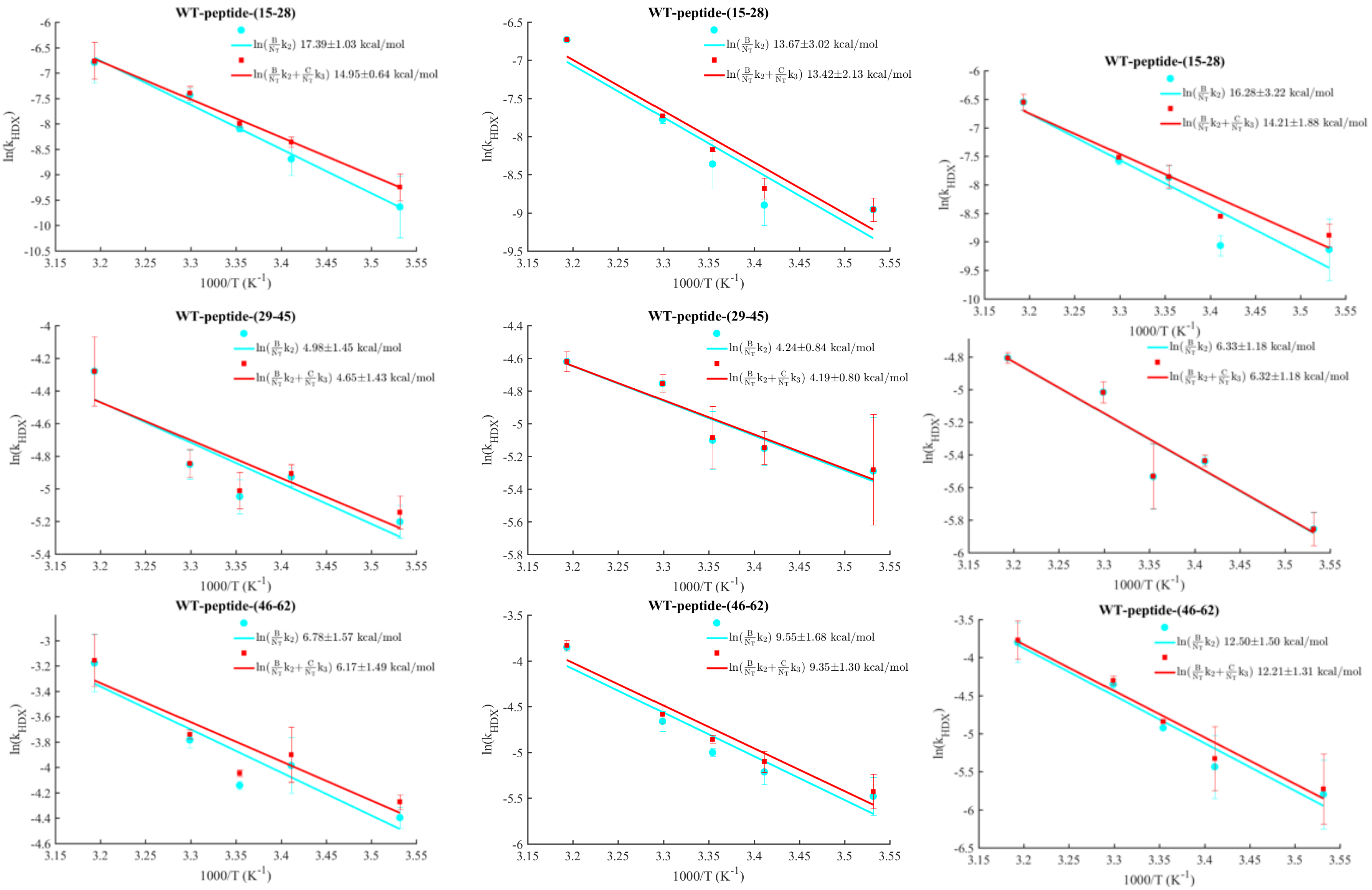
WT-peptide-(15-28)
ln(B/N_T k2) 17.39±1.03 kcal/mol
ln(B/N_T k2+C/N_T k3) 14.95±0.64 kcal/mol
WT-peptide-(15-28)
ln(B/N_T k2) 13.67±3.02 kcal/mol
ln(B/N_T k2+C/N_T k3) 13.42±2.13 kcal/mol
WT-peptide-(15-28)
ln(B/N_T k2) 16.28±3.22 kcal/mol
ln(B/N_T k2+C/N_T k3) 14.21±1.88 kcal/mol
WT-peptide-(29-45)
ln(B/N_T k2) 4.98±1.45 kcal/mol
ln(B/N_T k2+C/N_T k3) 4.65±1.43 kcal/mol
WT-peptide-(29-45)
ln(B/N_T k2) 4.24±0.84 kcal/mol
ln(B/N_T k2+C/N_T k3) 4.19±0.80 kcal/mol
ln(B/N_T k2) 6.33±1.18 kcal/mol
ln(B/N_T k2+C/N_T k3) 6.32±1.18 kcal/mol
WT-peptide-(46-62)
ln(B/N_T k2) 6.78±1.57 kcal/mol
ln(B/N_T k2+C/N_T k3) 6.17±1.49 kcal/mol
WT-peptide-(46-62)
ln(B/N_T k2) 9.55±1.68 kcal/mol
ln(B/N_T k2+C/N_T k3) 9.35±1.30 kcal/mol
WT-peptide-(46-62)
ln(B/N_T k2) 12.50±1.50 kcal/mol
ln(B/N_T k2+C/N_T k3) 12.21±1.31 kcal/mol
ln(k_HDX)
1000/T (K^-1)

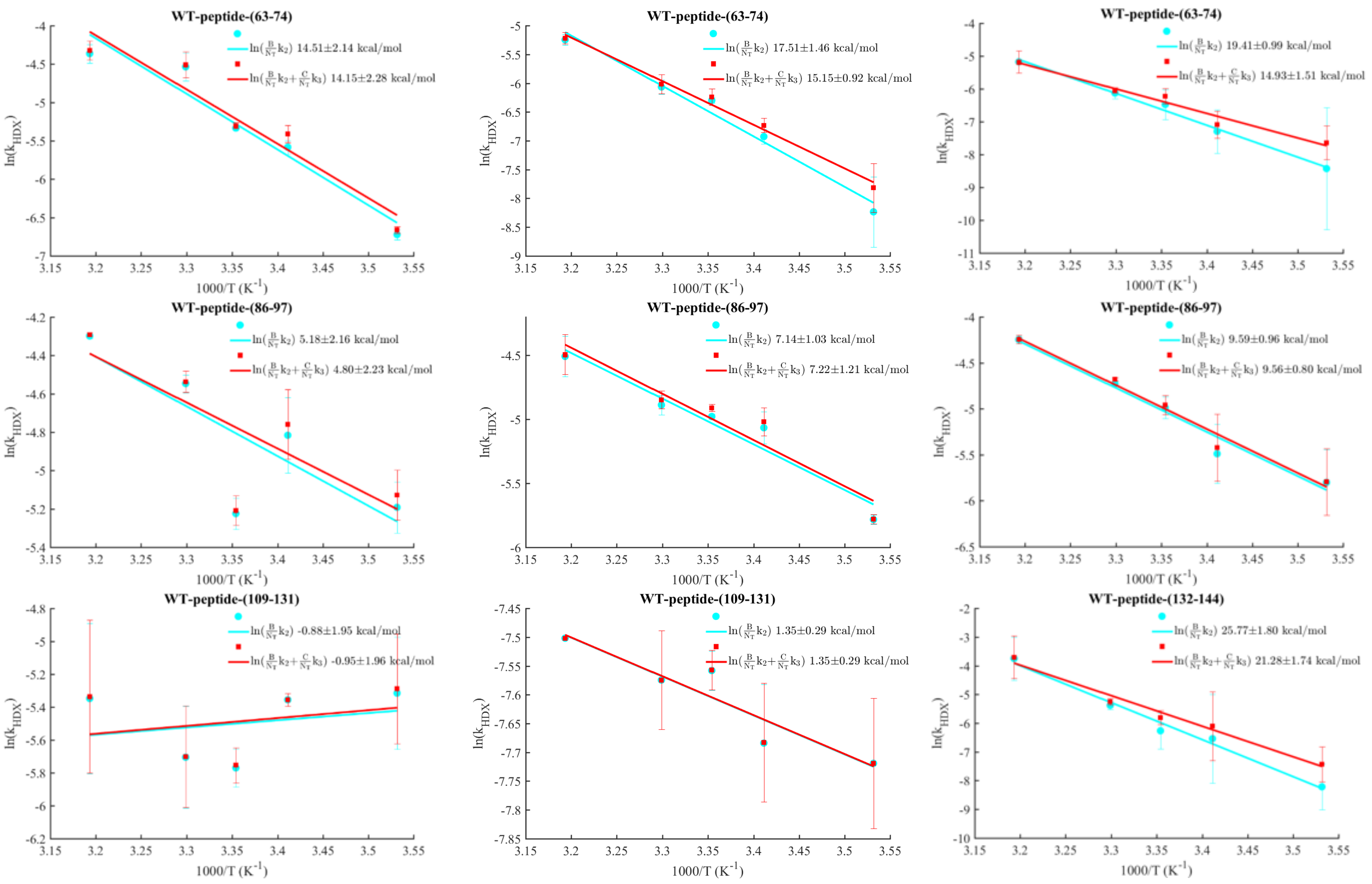

WT-peptide-(63-74)
ln(B/N_T k2) 14.51±2.14 kcal/mol
ln(B/N_T k2+C/N_T k3) 14.15±2.28 kcal/mol
WT-peptide-(63-74)
ln(B/N_T k2) 17.51±1.46 kcal/mol
ln(B/N_T k2+C/N_T k3) 15.15±0.92 kcal/mol
WT-peptide-(63-74)
ln(B/N_T k2) 19.41±0.99 kcal/mol
ln(B/N_T k2+C/N_T k3) 14.93±1.51 kcal/mol
WT-peptide-(86-97)
ln(B/N_T k2) 5.18±2.16 kcal/mol
ln(B/N_T k2+C/N_T k3) 4.80±2.23 kcal/mol
WT-peptide-(86-97)
ln(B/N_T k2) 7.14±1.03 kcal/mol
ln(B/N_T k2+C/N_T k3) 7.22±1.21 kcal/mol
WT-peptide-(86-97)
ln(B/N_T k2) 9.59±0.96 kcal/mol
ln(B/N_T k2+C/N_T k3) 9.56±0.80 kcal/mol
WT-peptide-(109-131)
ln(B/N_T k2) -0.88±1.95 kcal/mol
ln(B/N_T k2+C/N_T k3) -0.95±1.96 kcal/mol
WT-peptide-(109-131)
ln(B/N_T k2) 1.35±0.29 kcal/mol
ln(B/N_T k2+C/N_T k3) 1.35±0.29 kcal/mol
WT-peptide-(132-144)
ln(B/N_T k2) 25.77±1.80 kcal/mol
ln(B/N_T k2+C/N_T k3) 21.28±1.74 kcal/mol
ln(k_HDX)
1000/T (K^-1)

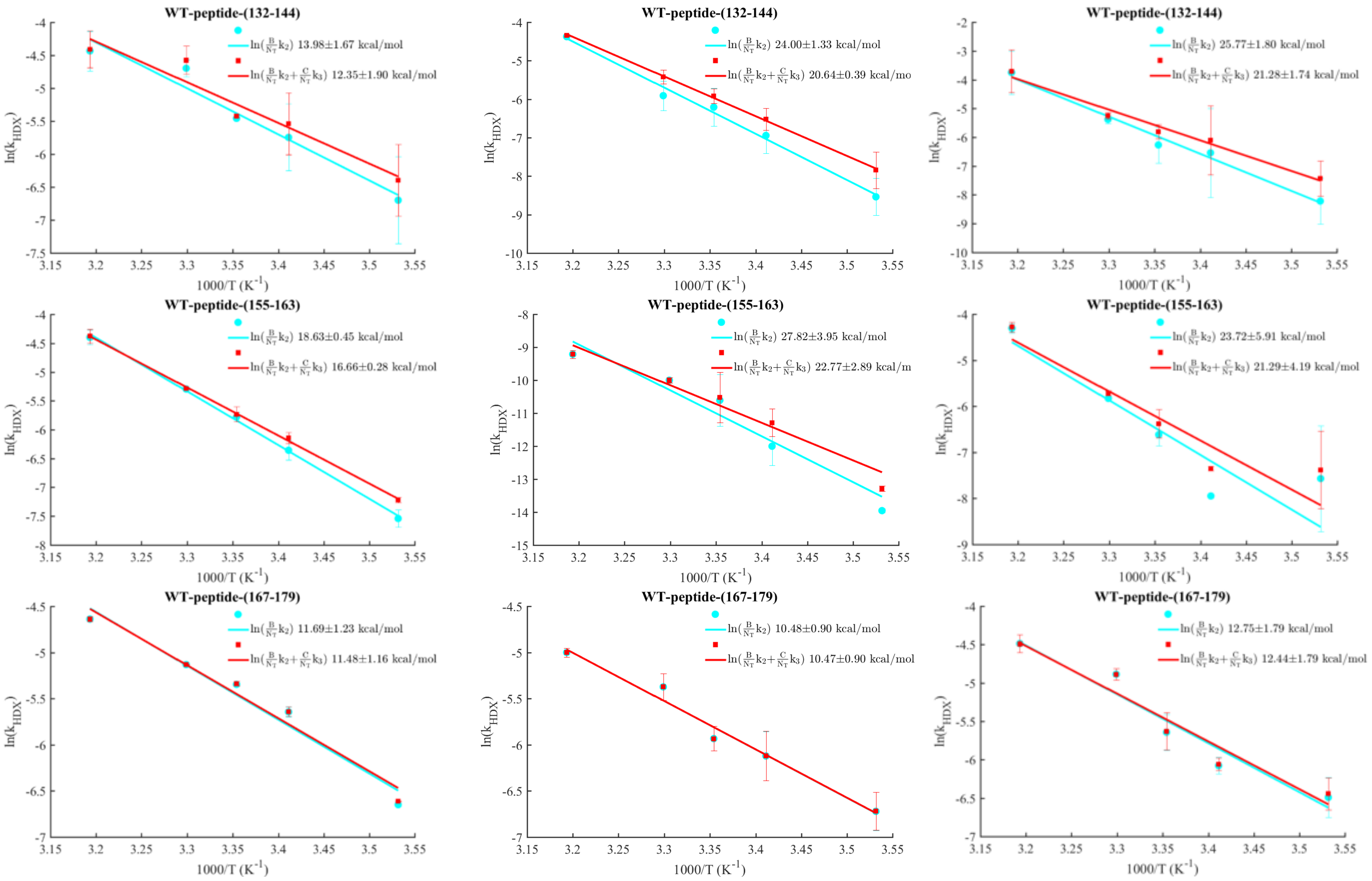
WT-peptide-(132-144)
ln(B/N_T k2) 13.98±1.67 kcal/mol
ln(B/N_T k2+C/N_T k3) 12.35±1.90 kcal/mol
WT-peptide-(132-144)
ln(B/N_T k2) 24.00±1.33 kcal/mol
ln(B/N_T k2+C/N_T k3) 20.64±0.39 kcal/mo
WT-peptide-(132-144)
ln(B/N_T k2) 25.77±1.80 kcal/mol
ln(B/N_T k2+C/N_T k3) 21.28±1.74 kcal/mol
WT-peptide-(155-163)
ln(B/N_T k2) 18.63±0.45 kcal/mol
ln(B/N_T k2+C/N_T k3) 16.66±0.28 kcal/mol
WT-peptide-(155-163)
ln(B/N_T k2) 27.82±3.95 kcal/mol
ln(B/N_T k2+C/N_T k3) 22.77±2.89 kcal/m
WT-peptide-(155-163)
ln(B/N_T k2) 23.72±5.91 kcal/mol
ln(B/N_T k2+C/N_T k3) 21.29±4.19 kcal/mol
WT-peptide-(167-179)
ln(B/N_T k2) 11.69±1.23 kcal/mol
ln(B/N_T k2+C/N_T k3) 11.48±1.16 kcal/mol
WT-peptide-(167-179)
ln(B/N_T k2) 10.48±0.90 kcal/mol
ln(B/N_T k2+C/N_T k3) 10.47±0.90 kcal/mol
WT-peptide-(167-179)
ln(B/N_T k2) 12.75±1.79 kcal/mol
ln(B/N_T k2+C/N_T k3) 12.44±1.79 kcal/mol
ln(k_HDX)
1000/T (K^-1)

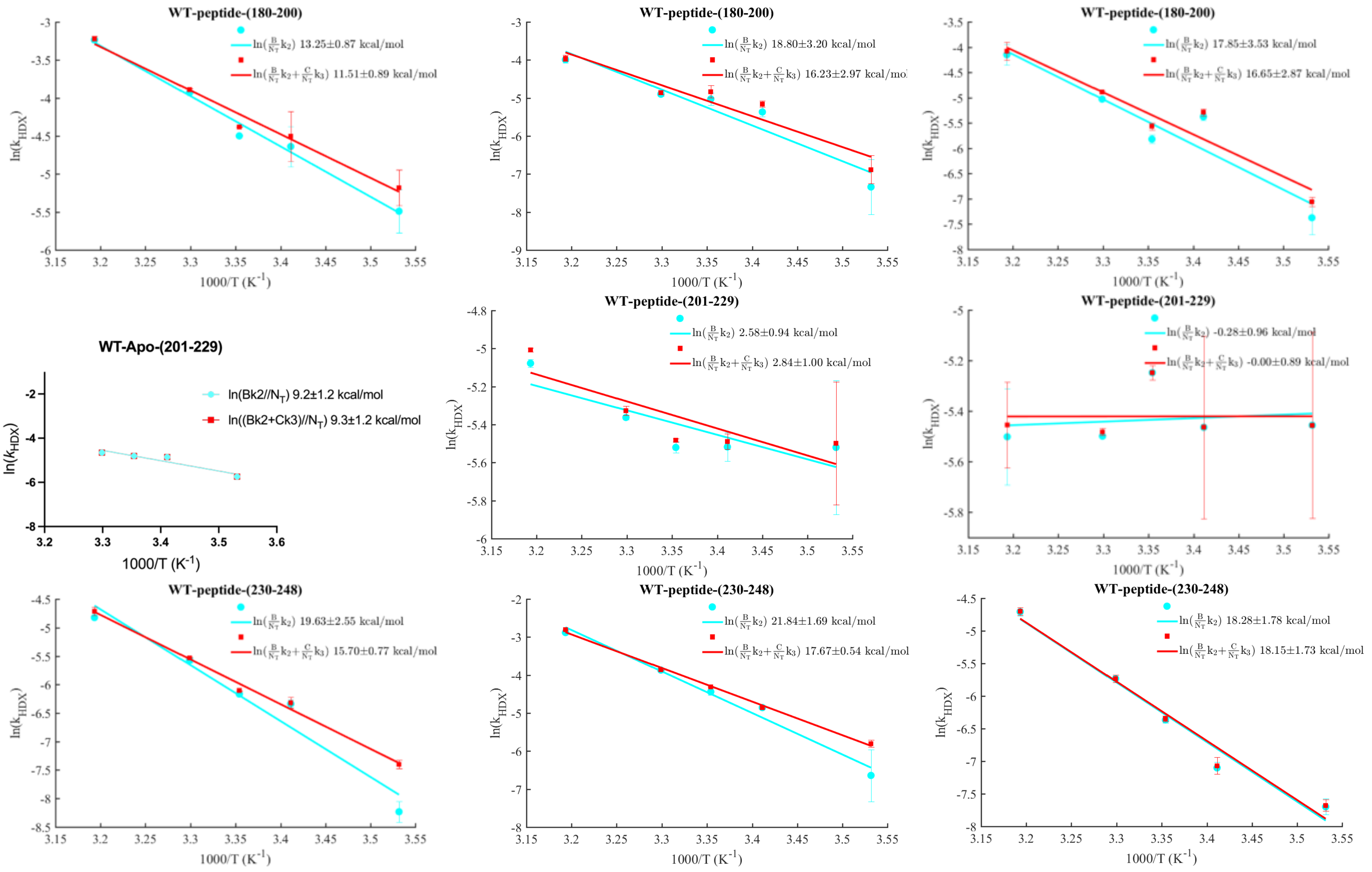
WT-peptide-(180-200)
ln(B/N_T k2) 13.25±0.87 kcal/mol
ln(B/N_T k2+C/N_T k3) 11.51±0.89 kcal/mol
WT-peptide-(180-200)
ln(B/N_T k2) 18.80±3.20 kcal/mol
ln(B/N_T k2+C/N_T k3) 16.23±2.97 kcal/mol
WT-peptide-(180-200)
ln(B/N_T k2) 17.85±3.53 kcal/mol
ln(B/N_T k2+C/N_T k3) 16.65±2.87 kcal/mol
WT-Apo-(201-229)
ln(Bk2//N_T) 9.2±1.2 kcal/mol
ln((Bk2+Ck3)//N_T) 9.3±1.2 kcal/mol
WT-peptide-(201-229)
ln(B/N_T k2) 2.58±0.94 kcal/mol
ln(B/N_T k2+C/N_T k3) 2.84±1.00 kcal/mol
WT-peptide-(201-229)
ln(B/N_T k2) -0.28±0.96 kcal/mol
ln(B/N_T k2+C/N_T k3) -0.00±0.89 kcal/mol
WT-peptide-(230-248)
ln(B/N_T k2) 19.63±2.55 kcal/mol
ln(B/N_T k2+C/N_T k3) 15.70±0.77 kcal/mol
WT-peptide-(230-248)
ln(B/N_T k2) 21.84±1.69 kcal/mol
ln(B/N_T k2+C/N_T k3) 17.67±0.54 kcal/mol
WT-peptide-(230-248)
ln(B/N_T k2) 18.28±1.78 kcal/mol
ln(B/N_T k2+C/N_T k3) 18.15±1.73 kcal/mol
ln(k_HDX)
1000/T (K^-1)

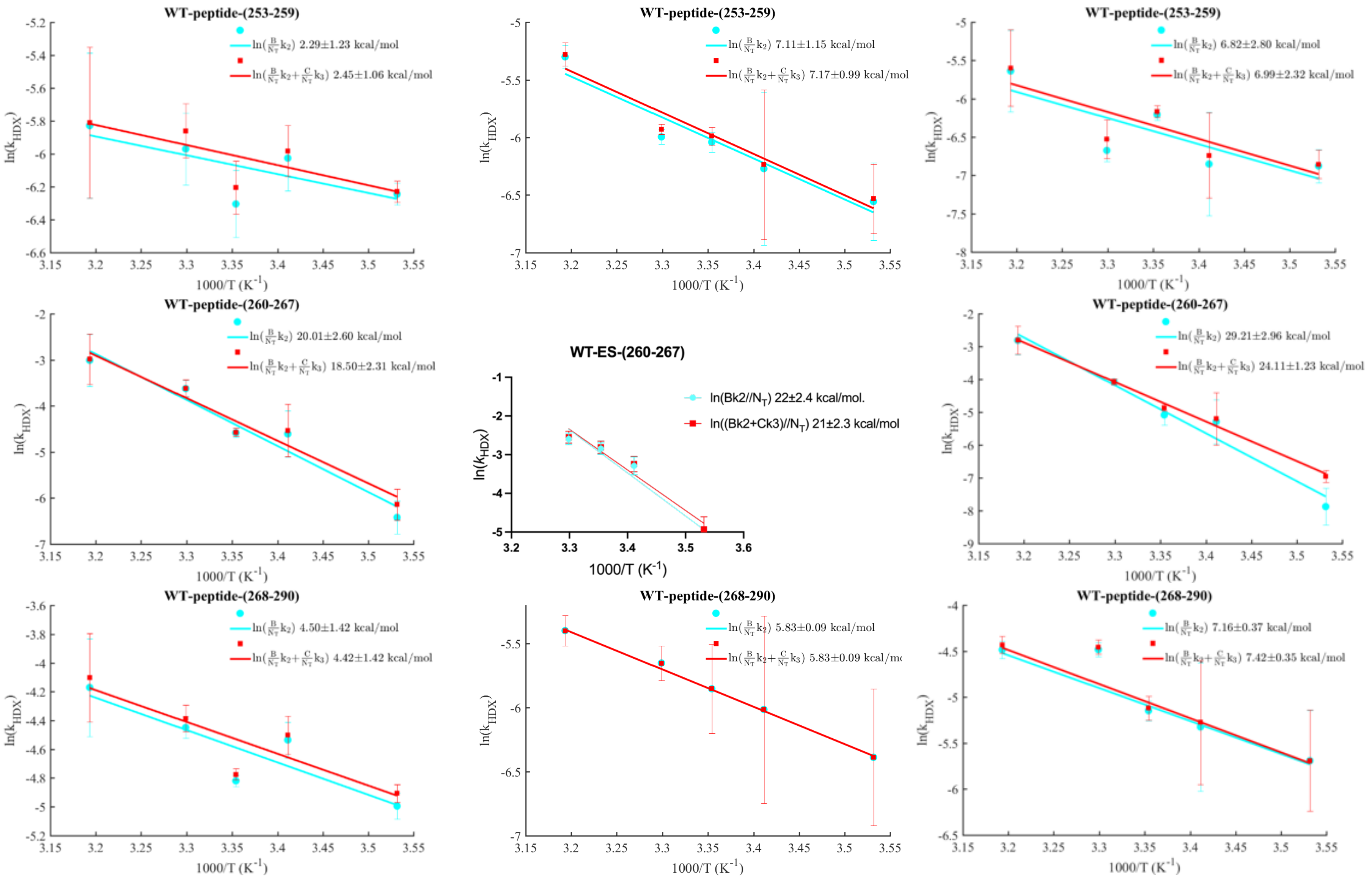

WT-peptide-(253-259)
ln(B/N_T k2) 2.29±1.23 kcal/mol
ln(B/N_T k2+C/N_T k3) 2.45±1.06 kcal/mol
WT-peptide-(253-259)
ln(B/N_T k2) 7.11±1.15 kcal/mol
ln(B/N_T k2+C/N_T k3) 7.17±0.99 kcal/mol
WT-peptide-(253-259)
ln(B/N_T k2) 6.82±2.80 kcal/mol
ln(B/N_T k2+C/N_T k3) 6.99±2.32 kcal/mol
WT-peptide-(260-267)
ln(B/N_T k2) 20.01±2.60 kcal/mol
ln(B/N_T k2+C/N_T k3) 18.50±2.31 kcal/mol
WT-ES-(260-267)
ln(Bk2//N_T) 22±2.4 kcal/mol.
ln((Bk2+Ck3)//N_T) 21±2.3 kcal/mol
WT-peptide-(260-267)
ln(B/N_T k2) 29.21±2.96 kcal/mol
ln(B/N_T k2+C/N_T k3) 24.11±1.23 kcal/mol
WT-peptide-(268-290)
ln(B/N_T k2) 4.50±1.42 kcal/mol
ln(B/N_T k2+C/N_T k3) 4.42±1.42 kcal/mol
WT-peptide-(268-290)
ln(B/N_T k2) 5.83±0.09 kcal/mol
ln(B/N_T k2+C/N_T k3) 5.83±0.09 kcal/m
WT-peptide-(268-290)
ln(B/N_T k2) 7.16±0.37 kcal/mol
ln(B/N_T k2+C/N_T k3) 7.42±0.35 kcal/mol
ln(k_HDX)
1000/T (K^-1)

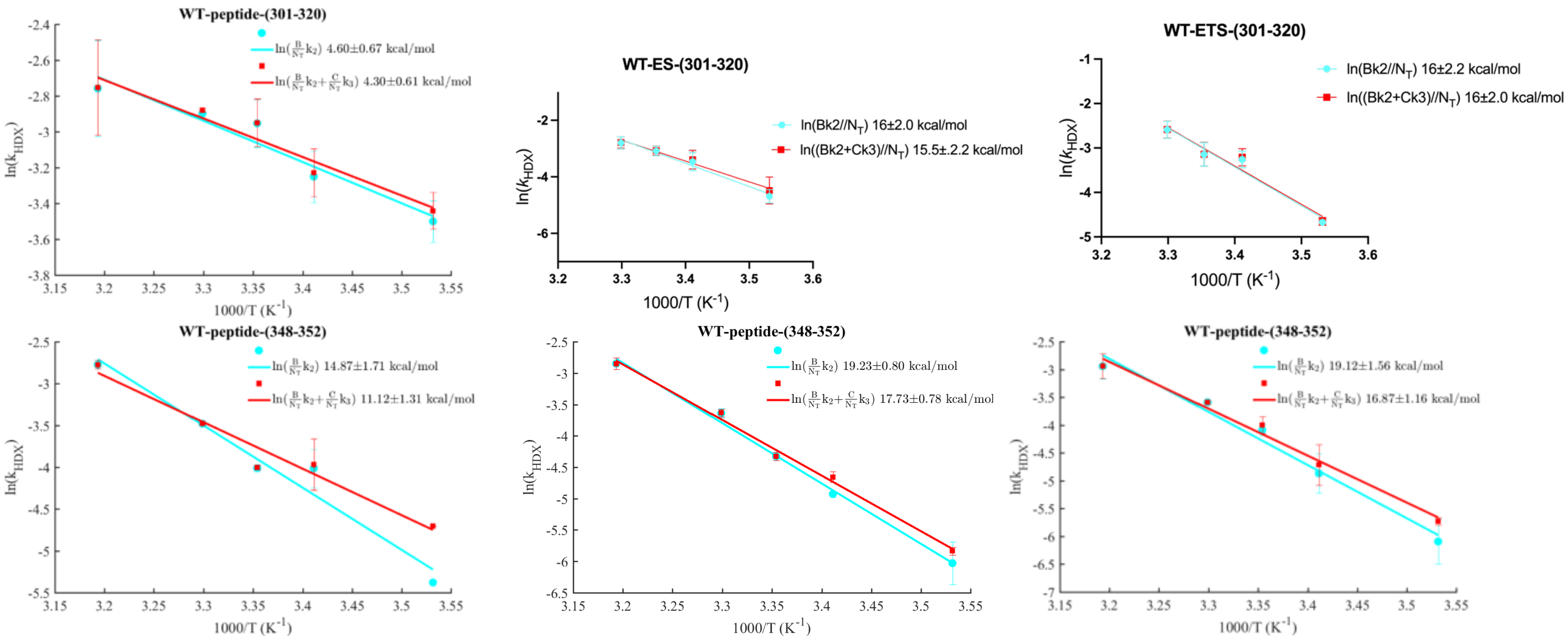
WT-peptide-(301-320)
ln(B/N_T k2) 4.60±0.67 kcal/mol
ln(B/N_T k2+C/N_T k3) 4.30±0.61 kcal/mol
ln(k_HDX)
1000/T (K^-1)
WT-ES-(301-320)
ln(Bk2//N_T) 16±2.0 kcal/mol
ln((Bk2+Ck3)//N_T) 15.5±.2.2 kcal/mol
ln(k_HDX)
1000/T (K^-1)
WT-ETS-(301-320)
ln(Bk2//N_T) 16±2.2 kcal/mol
ln((Bk2+Ck3)//N_T) 16±2.0 kcal/mol
ln(k_HDX)
1000/T (K^-1)
WT-peptide-(348-352)
ln(B/N_T k2) 14.87±1.71 kcal/mol
ln(B/N_T k2+C/N_T k3) 11.12±1.31 kcal/mol
ln(k_HDX)
1000/T (K^-1)
WT-peptide-(348-352)
ln(B/N_T k2) 19.23±0.80 kcal/mol
ln(B/N_T k2+C/N_T k3) 17.73±0.78 kcal/mol
ln(k_HDX)
1000/T (K^-1)
WT-peptide-(348-352)
ln(B/N_T k2) 19.12±1.56 kcal/mol
ln(B/N_T k2+C/N_T k3) 16.87±1.16 kcal/mol
ln(k_HDX)
1000/T (K^-1)

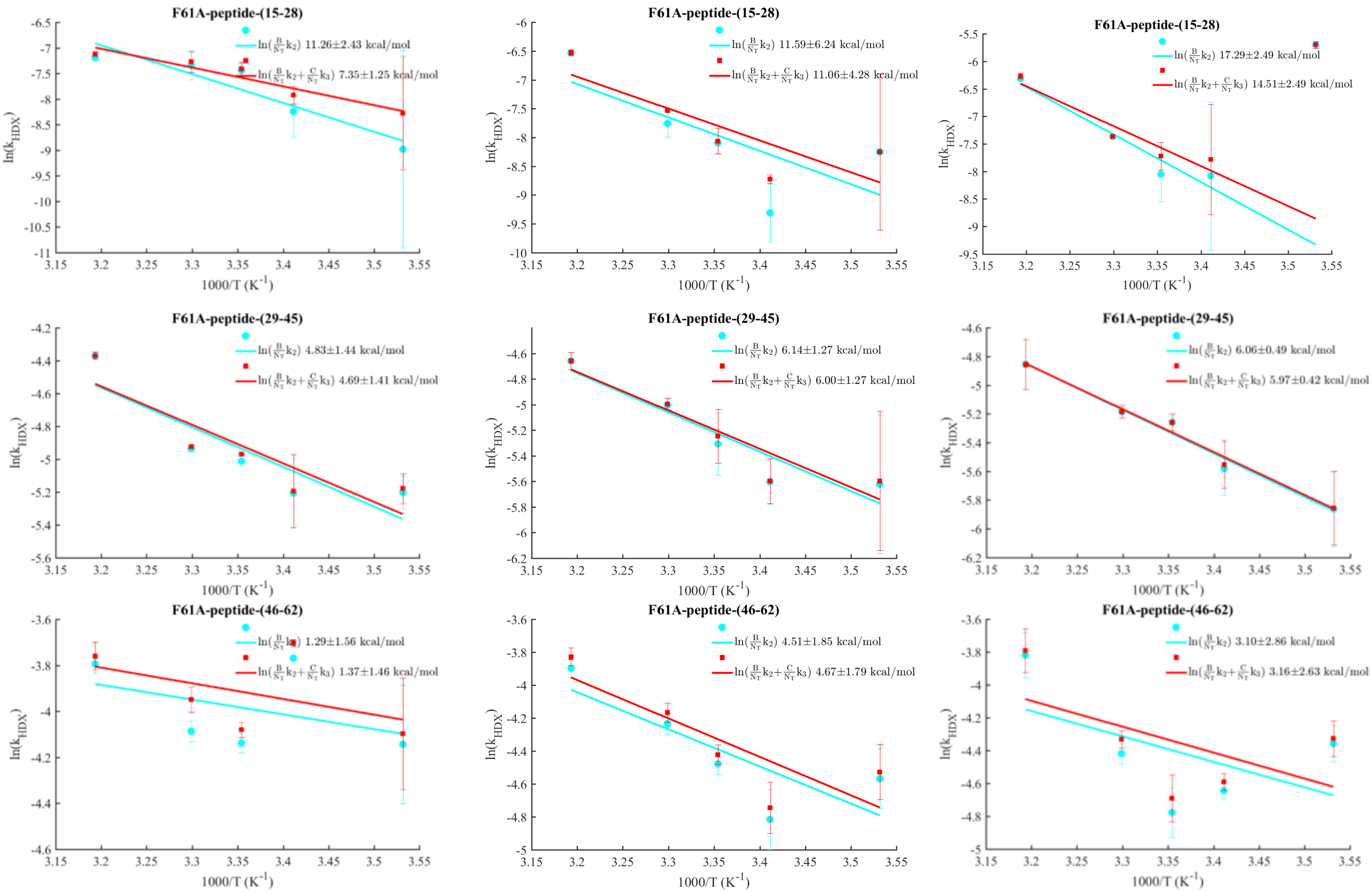
F61A-peptide-(15-28)
ln(B/N_T k2) 11.26±2.43 kcal/mol
ln(B/N_T k2+C/N_T k3) 7.35±1.25 kcal/mol
F61A-peptide-(15-28)
ln(B/N_T k2) 11.59±6.24 kcal/mol
ln(B/N_T k2+C/N_T k3) 11.06±4.28 kcal/mol
F61A-peptide-(15-28)
ln(B/N_T k2) 17.29±2.49 kcal/mol
ln(B/N_T k2+C/N_T k3) 14.51±2.49 kcal/mol
F61A-peptide-(29-45)
ln(B/N_T k2) 4.83±1.44 kcal/mol
ln(B/N_T k2+C/N_T k3) 4.69±1.41 kcal/mol
F61A-peptide-(29-45)
ln(B/N_T k2) 6.14±1.27 kcal/mol
ln(B/N_T k2+C/N_T k3) 6.00±1.27 kcal/mol
F61A-peptide-(29-45)
ln(B/N_T k2) 6.06±0.49 kcal/mol
ln(B/N_T k2+C/N_T k3) 5.97±0.42 kcal/mol
F61A-peptide-(46-62)
ln(B/N_T k2) 1.29±1.56 kcal/mol
ln(B/N_T k2+C/N_T k3) 1.37±1.46 kcal/mol
F61A-peptide-(46-62)
ln(B/N_T k2) 4.51±1.85 kcal/mol
ln(B/N_T k2+C/N_T k3) 4.67±1.79 kcal/mol
F61A-peptide-(46-62)
ln(B/N_T k2) 3.10±2.86 kcal/mol
ln(B/N_T k2+C/N_T k3) 3.16±2.63 kcal/mol
ln(k_HDX)
1000/T (K^-1)

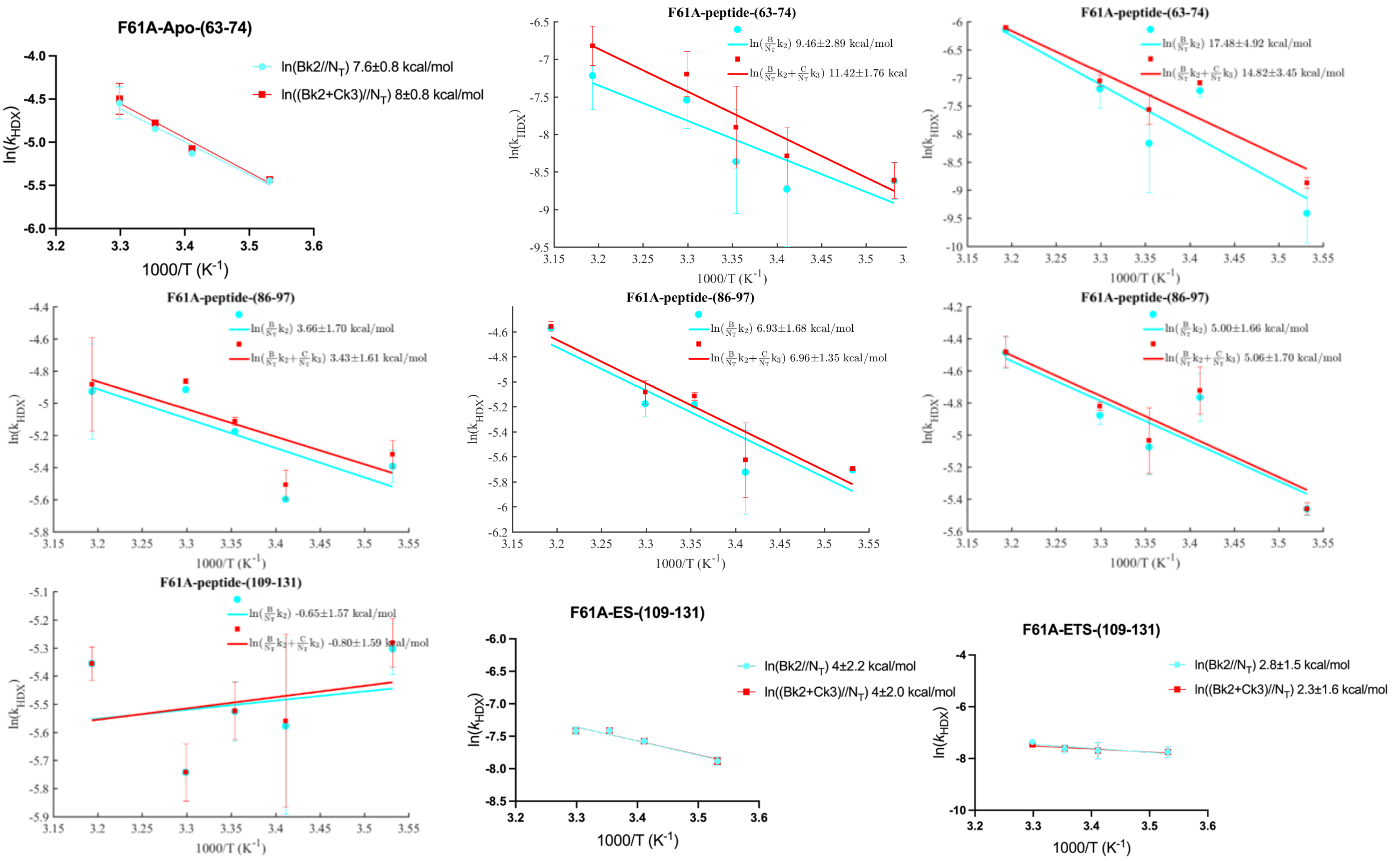
F61A-Apo-(63-74)
ln(Bk2//N_T) 7.6±0.8 kcal/mol
ln((Bk2+Ck3)//N_T) 8±0.8 kcal/mol
F61A-peptide-(63-74)
ln(B/N_T k2) 9.46±2.89 kcal/mol
ln(B/N_T k2+C/N_T k3) 11.42±1.76 kcal
F61A-peptide-(63-74)
ln(B/N_T k2) 17.48±4.92 kcal/mol
ln(B/N_T k2+C/N_T k3) 14.82±3.45 kcal/mol
F61A-peptide-(86-97)
ln(B/N_T k2) 3.66±1.70 kcal/mol
ln(B/N_T k2+C/N_T k3) 3.43±1.61 kcal/mol
F61A-peptide-(86-97)
ln(B/N_T k2) 6.93±1.68 kcal/mol
ln(B/N_T k2+C/N_T k3) 6.96±1.35 kcal/mol
F61A-peptide-(86-97)
ln(B/N_T k2) 5.00±1.66 kcal/mol
ln(B/N_T k2+C/N_T k3) 5.06±1.70 kcal/mol
F61A-peptide-(109-131)
ln(B/N_T k2) -0.65±1.57 kcal/mol
ln(B/N_T k2+C/N_T k3) -0.80±1.59 kcal/mol
F61A-ES-(109-131)
ln(Bk2//N_T) 4±2.2 kcal/mol
ln((Bk2+Ck3)//N_T) 4±2.0 kcal/mol
F61A-ETS-(109-131)
ln(Bk2//N_T) 2.8±1.5 kcal/mol
ln((Bk2+Ck3)//N_T) 2.3±1.6 kcal/mol
ln(k_HDX)
1000/T (K^-1)

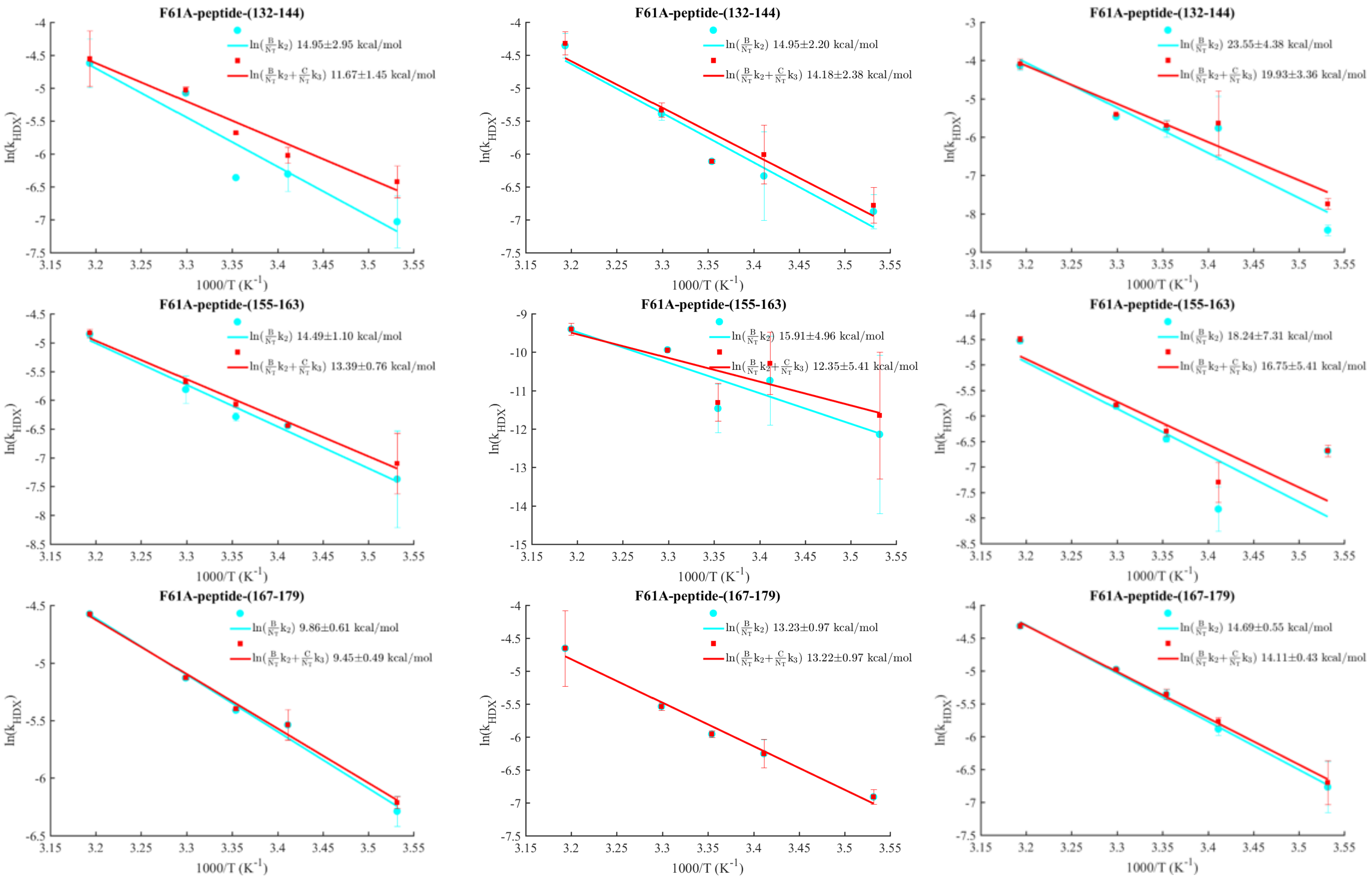
F61A-peptide-(132-144)
ln(B/NT k2) 14.95±2.95 kcal/mol
ln(B/NT k2+ C/NT k3) 11.67±1.45 kcal/mol
F61A-peptide-(132-144)
ln(B/NT k2) 14.95±2.20 kcal/mol
ln(B/NT k2+ C/NT k3) 14.18±2.38 kcal/mol
F61A-peptide-(132-144)
ln(B/NT k2) 23.55±4.38 kcal/mol
ln(B/NT k2+ C/NT k3) 19.93±3.36 kcal/mol
F61A-peptide-(155-163)
ln(B/NT k2) 14.49±1.10 kcal/mol
ln(B/NT k2+ C/NT k3) 13.39±0.76 kcal/mol
F61A-peptide-(155-163)
ln(B/NT k2) 15.91±4.96 kcal/mol
ln(B/NT k2+ C/NT k3) 12.35±5.41 kcal/mol
F61A-peptide-(155-163)
ln(B/NT k2) 18.24±7.31 kcal/mol
ln(B/NT k2+ C/NT k3) 16.75±5.41 kcal/mol
F61A-peptide-(167-179)
ln(B/NT k2) 9.86±0.61 kcal/mol
ln(B/NT k2+ C/NT k3) 9.45±0.49 kcal/mol
F61A-peptide-(167-179)
ln(B/NT k2) 13.23±0.97 kcal/mol
ln(B/NT k2+ C/NT k3) 13.22±0.97 kcal/mol
F61A-peptide-(167-179)
ln(B/NT k2) 14.69±0.55 kcal/mol
ln(B/NT k2+ C/NT k3) 14.11±0.43 kcal/mol
ln(kHDX)
1000/T (K-1)

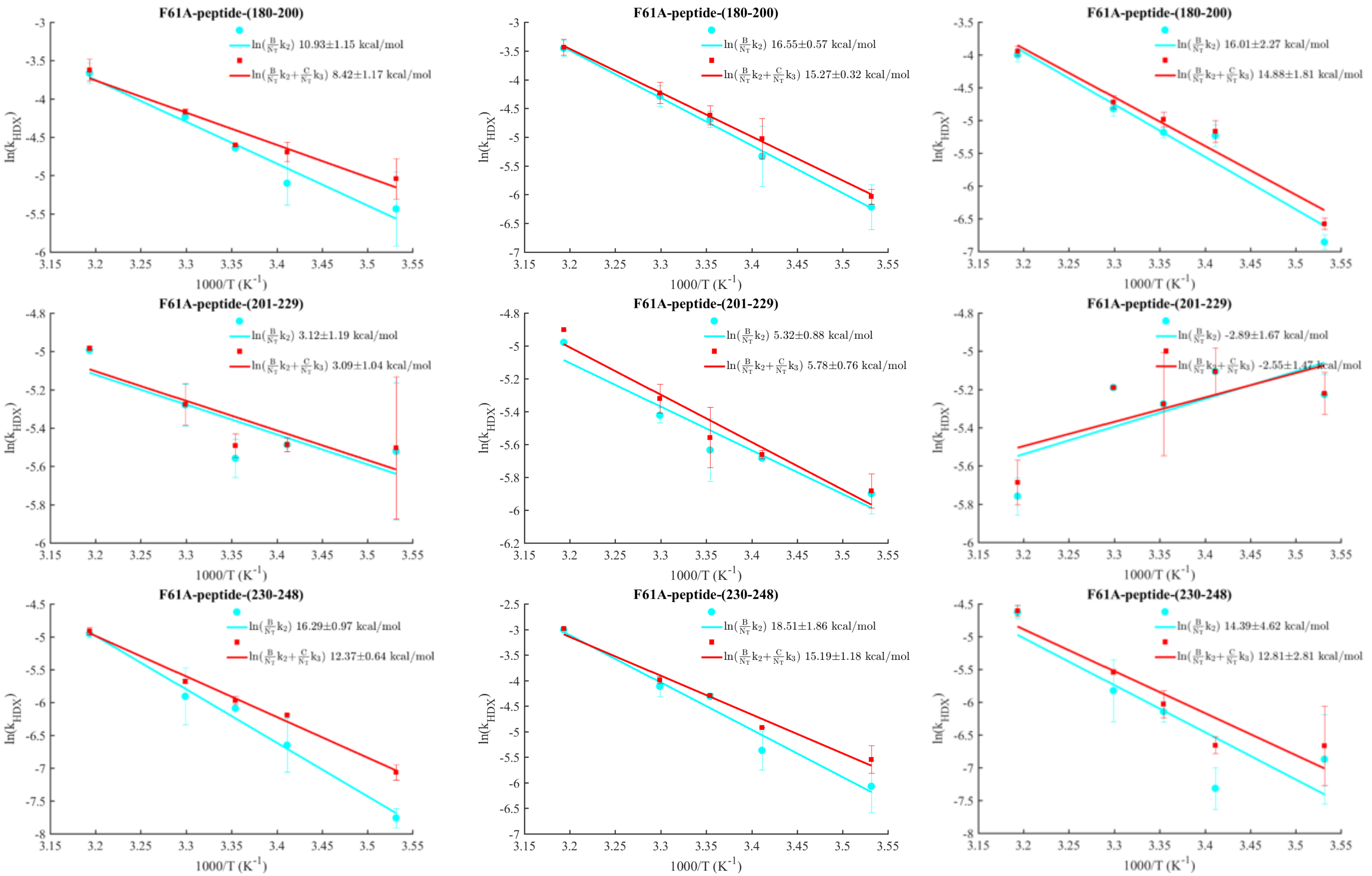
F61A-peptide-(180-200)
ln(B/N_T k2) 10.93±1.15 kcal/mol
ln(B/N_T k2+C/N_T k3) 8.42±1.17 kcal/mol
F61A-peptide-(180-200)
ln(B/N_T k2) 16.55±0.57 kcal/mol
ln(B/N_T k2+C/N_T k3) 15.27±0.32 kcal/mol
F61A-peptide-(180-200)
ln(B/N_T k2) 16.01±2.27 kcal/mol
ln(B/N_T k2+C/N_T k3) 14.88±1.81 kcal/mol
F61A-peptide-(201-229)
ln(B/N_T k2) 3.12±1.19 kcal/mol
ln(B/N_T k2+C/N_T k3) 3.09±1.04 kcal/mol
F61A-peptide-(201-229)
ln(B/N_T k2) 5.32±0.88 kcal/mol
ln(B/N_T k2+C/N_T k3) 5.78±0.76 kcal/mol
F61A-peptide-(201-229)
ln(B/N_T k2) -2.89±1.67 kcal/mol
ln(B/N_T k2+C/N_T k3) -2.55±1.47 kcal/mol
F61A-peptide-(230-248)
ln(B/N_T k2) 16.29±0.97 kcal/mol
ln(B/N_T k2+C/N_T k3) 12.37±0.64 kcal/mol
F61A-peptide-(230-248)
ln(B/N_T k2) 18.51±1.86 kcal/mol
ln(B/N_T k2+C/N_T k3) 15.19±1.18 kcal/mol
F61A-peptide-(230-248)
ln(B/N_T k2) 14.39±4.62 kcal/mol
ln(B/N_T k2+C/N_T k3) 12.81±2.81 kcal/mol
ln(k_HDX)
1000/T (K^-1)

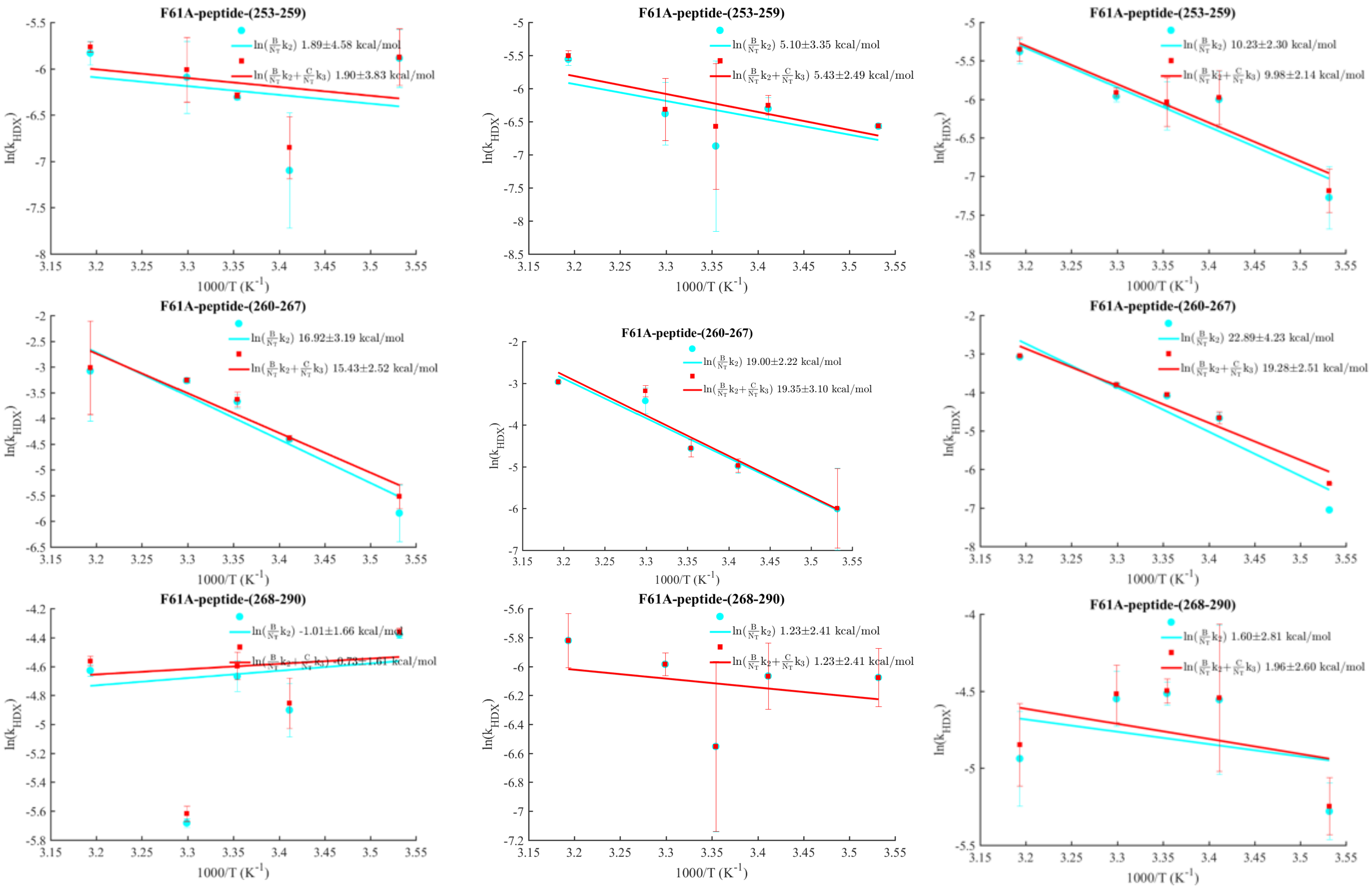
F61A-peptide-(253-259)
ln(B/N_T k2) 1.89±4.58 kcal/mol
ln(B/N_T k2+C/N_T k3) 1.90±3.83 kcal/mol
F61A-peptide-(253-259)
ln(B/N_T k2) 5.10±3.35 kcal/mol
ln(B/N_T k2+C/N_T k3) 5.43±2.49 kcal/mol
F61A-peptide-(253-259)
ln(B/N_T k2) 10.23±2.30 kcal/mol
ln(B/N_T k2+C/N_T k3) 9.98±2.14 kcal/mol
F61A-peptide-(260-267)
ln(B/N_T k2) 16.92±3.19 kcal/mol
ln(B/N_T k2+C/N_T k3) 15.43±2.52 kcal/mol
F61A-peptide-(260-267)
ln(B/N_T k2) 19.00±2.22 kcal/mol
ln(B/N_T k2+C/N_T k3) 19.35±3.10 kcal/mol
F61A-peptide-(260-267)
ln(B/N_T k2) 22.89±4.23 kcal/mol
ln(B/N_T k2+C/N_T k3) 19.28±2.51 kcal/mol
F61A-peptide-(268-290)
ln(B/N_T k2) -1.01±1.66 kcal/mol
F61A-peptide-(268-290)
ln(B/N_T k2) 1.23±2.41 kcal/mol
ln(B/N_T k2+C/N_T k3) 1.23±2.41 kcal/mol
F61A-peptide-(268-290)
ln(B/N_T k2) 1.60±2.81 kcal/mol
ln(B/N_T k2+C/N_T k3) 1.96±2.60 kcal/mol
ln(k_HDX)
1000/T (K^-1)

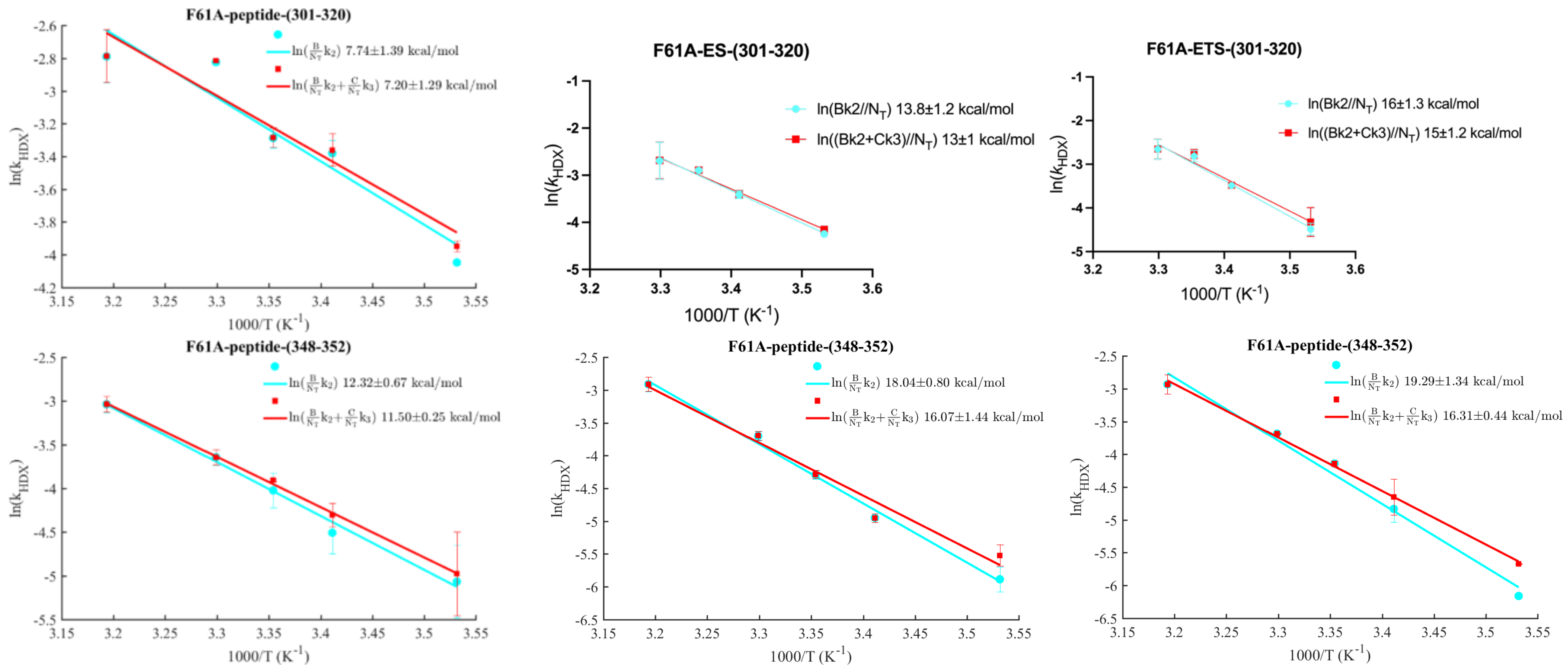

Figure S8. Arrhenius-like plots to obtain HDX activation energy for substate free (left), DAA bound (middle), and pentostatin (right) bound WT mADA and F61A mADA. Plots in cyan are generated using $B/N_T{*}k_2$ as the weighted rate constant, and red $B/N_T{*}k_2{+}C/N_T{*}k_3$. Weighted rate constants at each temperature for each peptide were from two biological replicates.

Tables S4-S9. Parameters resulting from the three-exponential fits for wild type mADA, F61A mADA at three different states (ligand free, substrate analog bound, and tight inhibitor bound). Exponential equation $y = N_T - A \cdot e^{-k_1 \cdot t} - B \cdot e^{-k_2 \cdot t} - C \cdot e^{-k_3 \cdot t} - N_{NE}$ was used. N_T stands for the total number of exchangeable amides within each peptide analyzed (see Table S1), and A, B, and C correspond to the numbers of amides exchanging with rate constants in the fast, intermediate and slow regimes represented by k_1, k_2, and k_3, respectively. N_{NE} represents the number of amides with no observable exchange within the experimental time period. N in Table S4-S9 equals the sum of fitted parameters of A, B, and C.

Table S4. Fitted parameters for wild type mADA in ligand free state (10).

'Sequence'	'Temperature'	'N'	'A'	'B'	'C'	'k1'	'k2'	'k3'
'15--28'	10	8.24123463	1.56971736	0.41873607	6.25278121	2.29659471	0.00152393	6.28E-05
'15--28'	20	3.85308175	1.6111917	1.38981219	0.85207786	5.3631155	0.00187151	0.00013927
'15--28'	25	3.56463676	1.67916835	0.76937579	1.11609262	10.3399481	0.00405915	0.00051008
'15--28'	30	7.86255934	1.66343528	1.04709735	5.15202671	15.0633668	0.00605639	5.99E-05
'15--28'	40	3.20006751	1.66930453	1.25497528	0.2757877	19.9652732	0.01007342	0.00082445
'29--45'	10	13.94144	6.29815512	2.05687421	5.58641066	8.56240835	0.03728585	0.0008165
'29--45'	20	13.9857979	6.9078455	2.61250795	4.46544443	11.6420097	0.03896624	0.00052069
'29--45'	25	13.9719825	7.10899835	2.57215165	4.29083247	12.7894758	0.0351313	0.00085687
'29--45'	30	11.4721838	7.30072784	3.31681341	0.85464256	14.548712	0.03296111	0.00061517
'29--45'	40	10.7323242	7.63777822	3.05854907	0.03599688	14.9027549	0.06401214	0.00023228
'46--62'	10	9.17056434	4.40427419	1.7806747	2.98561545	11.4599057	0.09018035	0.00702219
'46--62'	20	9.43278002	4.85998947	2.40365106	2.16913949	12.6723732	0.0995721	0.0116151
'46--62'	25	9.85133559	5.13000788	2.53700882	2.18431889	12.4815145	0.08147629	0.00962919
'46--62'	30	10.4829601	5.27987996	3.27176487	1.93131525	13.2759583	0.0900853	0.00665399
'46--62'	40	12.4168774	5.62202653	3.72217094	3.07267995	14.1490735	0.14725701	0.00236151
'63--74'	10	3.86639268	0.85683002	2.54624101	0.46332166	10.7229213	0.00447024	1.56E-05

'63--74'	20	8.94249242	0.83772467	1.32231305	6.78245469	21.2719529	0.0263753	0.00070932
'63--74'	25	8.9577872	0.97236908	2.14926023	5.83615789	24.5508684	0.0201233	0.00024858
'63--74'	30	8.99645574	0.88397628	2.06858389	6.04389556	10.9376646	0.04661351	0.00047563
'63--74'	40	4.30782318	1.05872111	2.12339758	1.12570449	3.29489797	0.0543435	0.00421393
'86--97'	10	9.99661178	2.45363651	1.51647754	6.02649772	14.7508998	0.03655851	0.00063009
'86--97'	20	9.98560567	2.64528443	1.83491045	5.50541079	11.1361655	0.04469599	0.00082785
'86--97'	25	6.33054905	3.0326322	2.13961213	1.15830473	9.61488924	0.02541398	0.00093173
'86--97'	30	9.83304185	2.84453394	2.55905983	4.42944807	13.7010685	0.04105013	0.00023632
'86--97'	40	9.98452709	3.03457341	2.5406598	4.40929388	12.5776275	0.05306056	0.00016198
'109--131'	10	17.9366748	7.4337734	1.88584975	8.61705159	10.8902152	0.04796135	0.00028967
'109--131'	20	10.4222234	8.2157178	2.09519919	0.11130641	12.8515356	0.04059868	9.45E-05
'109--131'	25	12.6218883	8.74875037	1.93202985	1.94110808	13.5449352	0.02894507	0.00033959
'109--131'	30	10.9792455	8.62917028	1.98090834	0.36916688	14.346611	0.03053209	0.00126769
'109--131'	40	17.996408	9.08525864	2.09835264	6.81279672	16.6169757	0.04201383	0.00026064
'132--144'	10	10.9562388	1.19921635	0.52793229	9.22909015	10.0797657	0.0223919	0.0005295
'132--144'	20	10.9315428	1.23180127	0.97264286	8.72709862	55.977874	0.03286017	0.00071335
'132--144'	25	10.8946773	1.44868815	1.82979804	7.61619115	17.7846979	0.02565581	0.00022696
'132--144'	30	3.98533487	1.34918927	1.87585934	0.76028627	15.8859722	0.04629313	0.00690514
'132--144'	40	10.9949152	1.35414553	2.3160595	7.32471013	10.4627908	0.05746872	0.00042013
'155--163'	10	3.00229332	1.89540703	0.43583695	0.67104934	1.7010792	0.00739029	0.00166302
'155--163'	20	5.99056632	1.94348355	0.72053099	3.32655179	3.94880437	0.01466799	0.00072894
'155--163'	25	5.88937233	1.84447585	1.38380684	2.66108964	8.48535587	0.0132432	0.00031456
'155--163'	30	5.83029632	1.9578919	1.58679382	2.2856106	9.94614169	0.01897754	0.00016527
'155--163'	40	5.99737377	1.98350031	1.44546428	2.56840918	15.0807742	0.05079417	0.00062812
'167--179'	10	5.96337544	2.8804544	2.14197672	0.94094432	1.84014433	0.00656357	0.00077325
'167--179'	20	5.6905594	2.89930437	2.5037014	0.28755363	4.44143675	0.01565552	0.00052551

'167--179'	25	6.83480036	3.00244142	2.60563327	1.22672567	8.66371295	0.02019738	0.00045956
'167--179'	30	6.36686441	3.07128538	2.93282152	0.36275752	9.6700088	0.02214383	2.50E-05
'167--179'	40	6.58477297	3.26522649	2.75344663	0.56609985	11.3066975	0.03847974	0.00014856
'180--200'	10	9.06482438	4.60436531	0.89385808	3.56660098	15.2568067	0.08375767	0.00759871
'180--200'	20	9.25614227	5.07813887	1.92497764	2.25302575	14.9222795	0.08184228	0.01758838
'180--200'	25	9.88293888	5.38350415	2.56365041	1.93578432	17.9207106	0.07828388	0.01278217
'180--200'	30	10.5181232	5.29171973	3.30447953	1.92192393	19.0780714	0.10676901	0.00803811
'180--200'	40	13.5787846	5.91320301	3.67547622	3.99010534	17.1228393	0.19228901	0.00234011
'201--229'	10	16.7213923	10.0807358	5.86460775	0.77604872	2.94610041	0.01433753	0.00014764
'201--229'	20	17.8343282	10.1866006	6.58974461	1.057983	9.87103947	0.03075139	1.32E-09
'201--229'	25	18.1646923	11.007385	6.48853807	0.66876922	11.0642058	0.03283142	1.59E-08
'201--229'	30	20.0979776	11.1424752	6.91614623	2.03935624	11.2390694	0.0356518	2.56E-08
'201--229'	40	25.9996998	12.1303267	6.31257195	7.55680117	9.12073998	0.04353984	0.00034202
'230--248'	10	9.62977709	3.93301058	2.8507733	2.84599322	12.3396453	0.00181785	0.0018175
'230--248'	20	7.52945926	3.98576305	3.26926199	0.27443422	27.5905134	0.00924425	9.96E-06
'230--248'	25	14.5607009	4.12252943	3.56019971	6.87797172	27.7147322	0.01032894	0.0001456
'230--248'	30	8.68826403	4.05186672	3.7822817	0.85411561	54.6870886	0.01720726	0.00247862
'230--248'	40	9.47203647	4.05575121	2.69846071	2.71782455	20.356447	0.05095055	0.00532149
'253--259'	10	1.20016221	0.66822574	0.42555032	0.10638615	19.9910471	0.02288069	0.00155108
'253--259'	20	2.89779376	0.7711771	0.52509821	1.60151845	18.2059401	0.0233324	0.00020173
'253--259'	25	4.99152883	0.81260864	0.51523161	3.66368858	10.9630184	0.01799753	0.00025101
'253--259'	30	4.99445683	0.88706063	0.73075127	3.37664492	10.4922706	0.01785816	0.00042931
'253--259'	40	2.85059479	1.00029881	1.34181663	0.50847935	2.88397775	0.01078196	3.02E-05
'260--267'	10	4.98086844	0.26756831	0.43004678	4.28325335	11.7213056	0.01850483	0.00059346
'260--267'	20	4.94760416	0.35161314	0.96983126	3.62615975	14.9942868	0.04070558	0.00043301
'260--267'	25	2.26836215	0.48762434	1.09300815	0.68772966	19.9655958	0.0468705	0.00096072

'260--267'	30	4.96808926	0.37874715	1.23673675	3.35260536	18.0111429	0.10839144	0.00039448
'260--267'	40	2.43688706	0.60630753	1.00565736	0.82492216	9.38407132	0.27351065	0.00813576
'268--290'	10	7.49632209	3.39131695	1.69307408	2.41193106	9.37668047	0.08088162	0.00529738
'268--290'	20	10.9577069	4.03640737	2.66071519	4.26058435	10.9408214	0.08073719	0.0016334
'268--290'	25	19.9725541	4.64770977	2.47319449	12.8516498	10.1042548	0.0649712	0.00057877
'268--290'	30	11.6487495	4.49184779	2.74954396	4.40735772	14.6132538	0.08411813	0.00352857
'268--290'	40	17.1951239	4.81369474	3.2241362	9.157293	12.5222468	0.10035113	0.00203146
'301--320'	10	8.89460179	4.13901321	2.80487529	1.95071328	16.9591361	0.19558694	0.01711442
'301--320'	20	8.94411316	4.78811366	3.46071542	0.69528409	14.9849029	0.20494162	0.02226948
'301--320'	25	17.7785727	4.98674994	3.4692534	9.32256933	18.1870209	0.2709229	0.00039728
'301--320'	30	9.60392383	5.42111317	2.83776364	1.34504702	15.4107718	0.3478388	0.01174489
'301--320'	40	17.6021106	6.1469193	2.81067461	8.64451673	13.1654458	0.41639506	0.00057449
'348--352'	10	2.26564432	0.79083086	0.31880788	1.15600558	11.406731	0.04336791	0.01151732
'348--352'	20	2.20865989	0.89588937	1.24152363	0.0712469	8.99888816	0.03768355	0.00713864
'348--352'	25	2.19160427	0.92963678	1.24354171	0.01842577	11.4640322	0.04359427	0.00208542
'348--352'	30	2.24471777	0.94516273	1.19224287	0.10731218	19.9682948	0.0775605	0.00584772
'348--352'	40	2.18269556	0.97784476	1.18943484	0.01541595	19.9062367	0.15713117	0.00224554

Table S5. Fitted parameters for wild type mADA in substrate analog bound state.

Sequence	Temperature	N	A	B	C	k1	k2	k3
15--28	10	10.92389	1.556979	9.308603	0.058306	1.919776	0.000158	3.17E-06
15--28	20	10.9721	1.705814	9.235949	0.030334	2.261819	0.000202	1.29E-05
15--28	25	5.358018	1.703813	1.909479	1.744727	3.783369	0.000944	0.000698
15--28	30	4.816301	1.694224	2.021319	1.100758	9.438254	0.002196	0.000347
15--28	40	3.599547	1.799896	1.619219	0.180432	19.9777	0.00812	0.000236
29--45	10	13.98893	5.963292	2.559855	5.465785	3.382506	0.027074	0.000115
29--45	20	10.2528	6.500419	3.081378	0.671005	8.81852	0.026446	0.000447

29--45	25	13.9987	7.065791	3.064027	3.868878	8.430852	0.027977	0.000434
29--45	30	12.21326	7.016791	3.19428	2.002192	11.72715	0.037911	4.15E-09
29--45	40	10.50581	7.783431	2.71286	0.009523	12.35363	0.050791	0.000144
46--62	10	12.98133	3.413197	1.540621	8.02751	4.296403	0.035537	0.000365
46--62	20	9.119772	3.647674	2.189316	3.282782	9.525876	0.03249	0.00256
46--62	25	11.28777	3.966302	2.357766	4.963704	8.477199	0.037624	0.002717
46--62	30	12.98558	4.059749	3.280732	5.645099	9.847212	0.037715	0.001785
46--62	40	11.60454	4.660843	4.477731	2.465963	11.20544	0.061626	0.002669
63--74	10	3.099961	0.819209	1.818112	0.46264	77.27422	0.001963	8.31E-06
63--74	20	6.261267	0.864916	0.569253	4.827098	16.53181	0.013336	0.000648
63--74	25	2.712976	0.805538	1.765289	0.142149	17.3064	0.008957	0.000392
63--74	30	7.066363	0.91063	1.424907	4.730826	68.69398	0.014818	0.000155
63--74	40	8.901108	0.951734	1.673658	6.275716	69.67671	0.028415	0.00024
86--97	10	4.39565	2.435673	1.52751	0.432468	20.24756	0.020196	0.00035
86--97	20	9.979988	2.591127	1.637237	5.751623	16.77365	0.038606	0.000518
86--97	25	9.97157	2.779764	1.52174	5.670066	10.0539	0.045243	0.000863
86--97	30	9.97853	2.860162	1.988843	5.129525	15.14966	0.038039	0.000606
86--97	40	9.78451	3.355422	2.404493	4.024594	14.11272	0.045993	0.000455
109--131	10	15.69881	8.580127	6.743273	0.375409	2.497178	0.001188	3.43E-07
109--131	20	15.05915	8.865913	5.851551	0.341685	5.625801	0.001475	2.55E-07
109--131	25	16.4414	9.21639	7.19231	0.032699	7.131377	0.00135	6.12E-07
109--131	30	15.28894	9.115134	5.715559	0.458242	10.08999	0.001634	1.67E-07
109--131	40	16.23087	9.613413	6.165586	0.45187	12.63098	0.001612	3.72E-08
132--144	10	10.78342	1.122236	4.830752	4.830435	51.45632	0.000477	0.000477
132--144	20	10.18683	1.042777	0.777986	8.366063	67.61996	0.014378	0.000562
132--144	25	3.062953	1.028697	1.594244	0.440012	28.95329	0.016557	0.004233
132--144	30	3.165231	1.102394	0.4662	1.596637	18.58089	0.06564	0.011413
132--144	40	10.997	1.227034	1.610579	8.15939	26.94472	0.085959	0.000628
155--163	10	3.109201	2.379831	0.193729	0.535642	1.908318	2.65E-05	8.73E-06
155--163	20	2.579698	2.473482	0.01942	0.086797	19.38334	0.003704	0.00054
155--163	25	5.601505	2.766688	1.968452	0.866366	5.823779	6.2E-05	4.1E-05

155--163	30	5.999857	2.94459	3.055123	0.000144	7.621713	9E-05	6.19E-07
155--163	40	5.999992	3.489024	2.51096	8.01E-06	8.32667	0.000243	4.38E-07
167--179	10	4.585641	2.682295	1.334361	0.568984	2.09572	0.009929	1.39E-05
167--179	20	6.165147	2.895705	2.298731	0.970711	2.450402	0.008811	6.05E-05
167--179	25	5.877	3.0392	2.661167	0.176633	3.300111	0.011015	6.69E-05
167--179	30	5.673766	2.834261	2.700981	0.138524	19.68876	0.020096	0.000125
167--179	40	9.523892	3.418044	2.396829	3.709019	7.49873	0.031018	7.63E-10
180--200	10	7.232573	4.629624	1.305215	1.297734	11.44346	0.007553	0.00755
180--200	20	8.846344	4.471099	1.180738	3.194507	12.58041	0.075426	0.00614
180--200	25	9.376299	4.637506	1.522255	3.216539	13.23624	0.077563	0.007913
180--200	30	17.99378	4.734517	3.138385	10.12088	11.00333	0.045054	0.000524
180--200	40	11.71087	5.122879	3.293313	3.294679	10.46576	0.108044	0.004348
201--229	10	25.72999	10.49246	5.019445	10.21809	10.70159	0.021554	8.9E-05
201--229	20	25.99655	9.993609	5.248221	10.75472	13.22797	0.020078	0.000171
201--229	25	25.92996	10.88998	5.175753	9.864227	8.858817	0.02022	0.000419
201--229	30	25.95751	11.00357	5.372077	9.581861	12.0347	0.022724	0.000451
201--229	40	25.99998	12.17245	5.905181	7.922351	13.06373	0.027399	0.001529
230--248	10	10.105	4.248985	0.233768	5.622247	11.19681	0.046951	0.007495
230--248	20	9.952691	4.299221	5.6517	0.00177	28.86802	0.023465	0.00403
230--248	25	10.43889	4.555679	4.328665	1.554548	15.0186	0.046656	0.01758
230--248	30	9.890827	4.387447	5.44428	0.0591	66.91274	0.06552	0.051272
230--248	40	9.990929	4.47207	4.440923	1.077936	85.2739	0.215553	0.059124
253--259	10	1.283678	0.68134	0.553231	0.049106	50.53497	0.013805	0.000745
253--259	20	4.962991	0.769548	0.531057	3.662385	12.27873	0.024572	0.000108
253--259	25	4.974025	0.850694	0.6	3.523331	4.825691	0.020659	0.000174
253--259	30	4.990347	0.850075	0.684924	3.455348	8.424714	0.019129	0.000264
253--259	40	2.883421	0.9586	1.082425	0.842396	8.655089	0.02304	0.000569
260--267	10	2.105155	0.437155	1.057574	0.610426	11.21868	0.029353	0.00527
260--267	20	2.397026	0.361636	1.175762	0.859628	71.57344	0.161788	0.009006
260--267	25	2.594413	0.419927	1.254605	0.919881	18.81191	0.232822	0.010939
260--267	30	2.486948	0.595071	1.106562	0.785315	11.38633	0.340518	0.020906

260--267	40	2.608193	1.329322	0.743704	0.535166	14.95342	0.248028	0.0326
268--290	10	7.983932	4.417272	3.175068	0.391592	5.186608	0.011272	1.9E-08
268--290	20	10.14324	4.632266	3.790586	1.720385	10.06928	0.017575	1.88E-08
268--290	25	11.21489	5.203123	3.852451	2.159313	3.881727	0.014928	3.47E-09
268--290	30	10.99841	4.995416	4.195512	1.807481	7.560122	0.018575	3.09E-09
268--290	40	13.13628	6.118874	6.077526	0.939877	8.135395	0.015205	1.87E-08
301--320	10	9.161364	4.710201	2.762174	1.688989	16.80818	0.056024	0.008929
301--320	20	8.36734	4.424714	2.881473	1.061152	24.75101	0.189778	0.035811
301--320	25	9.7811	5.00246	3.630985	1.147654	16.26064	0.227542	0.00475
301--320	30	9.39125	5.264953	3.137153	0.989144	17.47746	0.356141	0.008666
301--320	40	10.47495	6.791282	1.846796	1.836872	11.35574	0.41536	0.005928
348--352	10	1.73503	0.750888	0.53782	0.446323	2.390562	0.009128	0.008647
348--352	20	1.989592	0.758218	0.58619	0.645183	5.552949	0.037076	0.01048
348--352	25	2.010205	0.838888	1.162601	0.008717	9.677504	0.034152	0.00259
348--352	30	1.963739	0.835335	1.127291	0.001113	17.85196	0.070196	0.001658
348--352	40	1.998875	0.980754	1.00232	0.015801	14.97142	0.171978	0.000702

Table S6. Fitted parameters for wild type mADA in tight inhibitor bound state.

Sequence	Temperature	N	A	B	C	k1	k2	k3
15--28	10	4.085677	1.523936	0.685204	1.876537	1.834312	0.002065	8.62E-05
15--28	20	4.153334	1.658909	1.205265	1.28916	2.907565	0.001284	0.000479
15--28	25	3.579857	1.695168	1.164568	0.720121	5.252791	0.003293	0.000242
15--28	30	4.619921	1.635649	1.958728	1.025544	9.446684	0.002894	0.000336
15--28	40	3.650996	1.806249	1.634455	0.210293	19.94825	0.00966	0.000209
29--45	10	8.954308	6.477345	2.295629	0.181334	2.438296	0.017479	7.47E-05
29--45	20	10.84737	6.69759	3.109239	1.04054	9.641913	0.019567	0.000206
29--45	25	11.25727	7.190765	3.331644	0.734865	8.61215	0.016789	0.000159
29--45	30	10.56386	7.256474	3.215589	0.091795	11.74762	0.028916	9.87E-06
29--45	40	10.87911	7.947193	2.904923	0.026995	12.69456	0.039562	4.97E-05
46--62	10	10.82836	2.762874	1.035069	7.030419	3.456255	0.036709	0.000585

46--62	20	8.458912	3.024906	1.490636	3.94337	9.797001	0.034703	0.002041
46--62	25	9.916777	3.129756	1.897718	4.889303	11.03917	0.049834	0.001691
46--62	30	8.669231	3.034136	2.381459	3.253636	14.38052	0.070347	0.002854
46--62	40	10.78372	3.552854	2.699735	4.531129	11.60474	0.108188	0.002128
63--74	10	1.672335	0.833055	0.419894	0.419386	62.96222	0.005833	0.005833
63--74	20	3.55411	1.026119	0.370644	2.157347	9.416657	0.013143	0.001636
63--74	25	2.263726	0.922916	1.336826	0.003983	9.033617	0.013512	0.000205
63--74	30	2.639669	0.873127	0.37967	1.386872	11.3811	0.047927	0.007326
63--74	40	2.562813	1.059963	1.492243	0.010607	8.118538	0.03913	0.00266
86--97	10	4.150164	2.529099	1.479102	0.141964	19.87081	0.020665	0.001026
86--97	20	9.998878	3.048161	1.49038	5.460337	9.04466	0.02601	0.000663
86--97	25	9.889059	3.103743	2.167383	4.617933	11.63883	0.031679	0.000431
86--97	30	8.660473	3.024312	1.997776	3.638385	10.91343	0.044185	0.001267
86--97	40	6.907071	3.569999	2.571652	0.76542	12.13318	0.05513	0.000402
109--131	10	16.253	8.374236	7.560143	0.318626	2.219463	0.000987	1.11E-07
109--131	20	16.44378	8.481036	7.849525	0.113222	8.27077	0.001378	2.24E-07
109--131	25	16.70644	8.875336	7.627677	0.203425	6.229892	0.00136	1.23E-07
109--131	30	15.90121	8.90239	6.44397	0.554852	9.268557	0.00164	6.16E-05
109--131	40	17.63154	9.436759	8.03976	0.155016	11.52034	0.001218	1.88E-07
132--144	10	2.298832	1.253092	0.523961	0.521779	23.07907	0.007348	0.007347
132--144	20	10.98856	1.568643	0.737953	8.681969	9.476856	0.015432	0.00046
132--144	25	3.510447	1.471363	1.021598	1.017486	16.09424	0.016545	0.016545
132--144	30	3.908585	1.32583	1.041338	1.541417	19.57463	0.0423	0.010103
132--144	40	4.756535	1.50126	1.510989	1.744286	72.31839	0.166399	0.012211
155--163	10	4.336304	1.856698	1.328341	1.151265	1.240337	0.000643	0.00055
155--163	20	3.428823	1.959603	0.734503	0.734716	2.08079	0.002743	0.002601
155--163	25	5.992041	1.922064	0.551353	3.518624	3.631222	0.011526	0.000889
155--163	30	5.959626	1.904198	0.989737	3.06569	8.282117	0.017557	0.000798
155--163	40	5.997286	1.997636	1.32175	2.6779	16.97176	0.061663	0.000906
167--179	10	10.98694	2.76981	1.034904	7.182223	2.412037	0.017003	0.000108
167--179	20	6.620671	2.954785	2.679846	0.98604	6.552868	0.010054	3.49E-05

167--179	25	10.46819	3.126013	2.774447	4.567735	6.531727	0.014361	4.35E-05
167--179	30	6.512296	3.05899	2.867036	0.58627	11.81993	0.0291	0.000103
167--179	40	6.771594	3.524221	2.505536	0.741837	11.04831	0.048852	1.04E-08
180--200	10	6.043278	4.466707	1.498342	0.078229	22.87843	0.009431	2.45E-06
180--200	20	17.95145	4.470898	1.04157	12.43898	19.78786	0.078664	0.000574
180--200	25	8.368791	4.68648	1.082424	2.599887	14.46188	0.049547	0.005914
180--200	30	8.914731	4.50277	1.429202	2.982759	19.37733	0.082887	0.006243
180--200	40	10.00259	4.95164	2.334346	2.716606	18.59582	0.121798	0.00732
201--229	10	18.0281	9.801009	4.56964	3.657447	8.359818	0.025166	1.74E-09
201--229	20	15.60953	10.80191	4.651133	0.156491	10.2386	0.023952	9.2E-05
201--229	25	19.77116	11.71346	4.166278	3.891432	8.01142	0.033216	3.64E-09
201--229	30	25.99147	11.69312	3.811149	10.4872	8.196929	0.027922	0.000182
201--229	40	25.99929	13.25187	4.605666	8.141756	9.989781	0.023502	0.000614
230--248	10	7.154398	4.025796	1.260684	1.867918	8.227735	0.00635	2.92E-05
230--248	20	10.79235	4.269209	5.586113	0.937029	15.76479	0.002576	0.000106
230--248	25	9.197609	4.256321	3.868845	1.072444	12.78382	0.007518	0.000599
230--248	30	8.40318	4.061542	3.817808	0.523829	19.83348	0.014426	0.000465
230--248	40	16.87622	4.378923	3.699549	8.797746	19.3347	0.041598	0.000138
253--259	10	1.121886	0.704913	0.375273	0.041699	20.01313	0.014335	0.000633
253--259	20	1.535582	0.844117	0.417327	0.274138	19.93319	0.014671	0.001411
253--259	25	1.952872	0.854449	0.559059	0.539364	19.94655	0.018717	0.000797
253--259	30	3.661984	0.89781	1.248152	1.516023	2.425147	0.004299	8.6E-05
253--259	40	4.967139	1.097963	0.928736	2.94044	2.80723	0.018821	0.000154
260--267	10	1.596996	0.380557	0.610115	0.606323	16.44078	0.003922	0.003922
260--267	20	1.543488	0.506523	1.028824	0.00814	5.254394	0.017956	0.000104
260--267	25	1.602985	0.458156	1.1447	0.000129	13.30875	0.034079	0.001254
260--267	30	4.811666	0.380394	1.088845	3.342427	25.13614	0.077317	0.000218
260--267	40	2.531512	0.500525	1.121579	0.909408	56.29572	0.272807	0.00209
268--290	10	6.16094	4.012525	2.018581	0.129834	13.04784	0.033892	0.001369
268--290	20	19.96482	4.402436	1.706077	13.8563	8.632872	0.056622	0.000238
268--290	25	18.48391	4.752619	2.194152	11.53714	6.276784	0.051371	0.000212

268--290	30	19.97076	4.317142	2.140115	13.5135	15.86928	0.106855	0.000467
268--290	40	14.87033	5.568345	1.911475	7.390507	11.51373	0.121719	0.001738
301--320	10	17.99596	4.41244	3.331697	10.25182	17.04987	0.05034	0.000347
301--320	20	8.805375	4.510794	2.955518	1.339064	23.46081	0.228354	0.020018
301--320	25	17.76067	5.021676	3.403972	9.335022	15.31906	0.223664	0.000383
301--320	30	17.99872	4.928905	3.444715	9.625099	18.81654	0.39662	0.00042
301--320	40	17.72937	6.542921	2.033281	9.153172	12.28584	0.476728	0.000656
348--352	10	1.90118	0.816477	0.533154	0.551549	1.676659	0.008734	0.008222
348--352	20	2.18612	0.885041	0.593658	0.707421	5.004886	0.02961	0.008637
348--352	25	2.18944	0.843094	0.740654	0.605692	8.497841	0.066121	0.010509
348--352	30	2.117401	0.869794	1.24484	0.002767	11.16182	0.06647	0.00163
348--352	40	2.172914	1.03957	1.055264	0.078079	13.06287	0.151348	0.000346

Table S7. Fitted parameters for F61A mADA in ligand free state.

'Sequence'	'Temperature'	'N'	'A'	'B'	'C'	'k1'	'k2'	'k3'
**'15--28'**	10	2.104604	1.388347	0.632353	0.083904	19.94049	0.015404	0.000363
**'15--28'**	20	3.197393	1.612959	0.83313	0.751304	4.301597	0.003863	0.000978
**'15--28'**	25	3.494367	1.551815	1.500606	0.441946	11.40985	0.004379	0.000302
**'15--28'**	30	3.991118	1.636868	1.051723	1.302527	11.77667	0.007035	0.000279
**'15--28'**	40	3.935013	1.781071	1.297418	0.856525	10.47583	0.006443	0.000686
**'29--45'**	10	13.95493	6.381848	2.696873	4.876208	7.46825	0.029045	0.000438
**'29--45'**	20	11.23478	6.926025	2.976729	1.332022	10.21937	0.025089	0.000397
**'29--45'**	25	13.89722	6.783902	2.396258	4.717064	13.17973	0.038885	0.000863
**'29--45'**	30	13.98592	7.155348	2.942812	3.887757	12.49072	0.033895	0.000223
**'29--45'**	40	10.58427	7.463433	3.099979	0.020859	14.42737	0.05715	0.000521
**'46--62'**	10	12.94179	4.476784	2.02947	6.43554	2.58024	0.102715	0.001447
**'46--62'**	20	10.14274	4.495614	2.257791	3.389332	8.114955	0.134275	0.006318
**'46--62'**	25	12.98526	4.909201	2.522967	5.553095	8.437389	0.082292	0.002199
**'46--62'**	30	10.05744	5.2169	2.179189	2.661349	7.32566	0.100339	0.012238

**'46--62'**	40	10.52194	5.713267	3.545991	1.262682	8.731561	0.082208	0.00795
**'63--74'**	10	3.588597	0.897196	1.890062	0.801339	14.34398	0.021094	0.00065
**'63--74'**	20	4.968031	1.009014	1.418986	2.540031	8.540741	0.039323	0.001261
**'63--74'**	25	4.338604	0.972845	1.382271	1.983488	16.64669	0.05142	0.002464
**'63--74'**	30	8.718654	1.066633	1.445471	6.20655	24.64463	0.066468	0.000765
**'63--74'**	40	8.944931	1.495526	1.481522	5.967884	4.348001	0.048248	0.000488
**'86--97'**	10	9.997626	2.541614	1.213358	6.242654	10.89984	0.037719	0.000567
**'86--97'**	20	9.983126	2.741817	1.418353	5.822957	10.02297	0.026318	0.000737
**'86--97'**	25	9.980517	2.457802	1.596209	5.926506	15.76318	0.035271	0.000632
**'86--97'**	30	9.99966	2.790434	1.801356	5.407869	15.86459	0.040709	0.000738
**'86--97'**	40	9.997893	3.058751	1.989494	4.949648	8.234523	0.04012	0.000692
**'109--131'**	10	17.99668	7.987192	2.223552	7.785938	11.90625	0.040022	0.000272
**'109--131'**	20	17.98741	8.37279	1.971347	7.643274	11.44636	0.035641	0.000209
**'109--131'**	25	11.31942	8.444061	1.975857	0.899498	14.25383	0.036321	7.59E-05
**'109--131'**	30	11.97686	8.851641	1.849335	1.275882	14.5678	0.031263	4.00E-08
**'109--131'**	40	10.94614	8.919697	1.963461	0.062984	15.27727	0.043386	1.72E-05
**'132--144'**	10	4.676788	1.135415	0.300627	3.240746	9.704417	0.029742	0.003127
**'132--144'**	20	10.93406	1.355526	1.073899	8.504638	11.10383	0.019167	0.00073
**'132--144'**	25	4.024397	1.259067	1.384525	1.380805	79.85943	0.013597	0.013597
**'132--144'**	30	10.86051	1.330367	1.968103	7.562041	62.31077	0.033619	0.000366
**'132--144'**	40	10.99412	1.456923	1.907497	7.629703	11.06757	0.048995	0.000537
**'155--163'**	10	2.734337	1.894962	0.257086	0.582288	1.604424	0.017817	0.001296
**'155--163'**	20	2.936948	1.938728	0.990129	0.008091	3.174974	0.009539	0.000284
**'155--163'**	25	5.961974	1.762211	0.808702	3.391061	6.410805	0.013793	0.000799
**'155--163'**	30	3.370505	1.864011	0.980188	0.526306	10.01237	0.01391	0.013091
**'155--163'**	40	5.928909	2.015476	1.48723	2.426203	10.61619	0.03161	0.00041
**'167--179'**	10	10.77774	2.988726	1.691122	6.097892	3.249431	0.01228	0.000206
**'167--179'**	20	5.669348	3.06728	2.521071	0.080997	8.480925	0.017215	0.000433
**'167--179'**	25	7.084844	2.901126	2.655447	1.528271	11.18802	0.018502	0.000545
**'167--179'**	30	6.377031	3.225012	2.745067	0.406952	11.36743	0.023668	4.74E-05
**'167--179'**	40	7.389551	3.416725	2.73788	1.234946	10.98982	0.04113	2.50E-08

**'180--200'**	10	9.180881	4.673536	0.940618	3.566726	38.80122	0.088177	0.009699
**'180--200'**	20	9.15857	4.974231	1.251771	2.932567	12.91786	0.088786	0.018568
**'180--200'**	25	17.98258	5.051526	3.198557	9.7325	19.60371	0.053889	0.000601
**'180--200'**	30	10.50078	5.37566	2.933397	2.191723	16.66022	0.089348	0.007741
**'180--200'**	40	10.78291	5.75245	3.25747	1.772989	13.62456	0.140345	0.011505
**'201--229'**	10	25.9983	9.536737	4.024437	12.43712	3.439819	0.023018	0.000183
**'201--229'**	20	19.01752	9.924518	5.105103	3.987901	7.213098	0.021153	5.92E-09
**'201--229'**	25	25.97193	9.844646	5.200848	10.92643	11.45952	0.019387	0.000616
**'201--229'**	30	25.96384	10.34687	5.881666	9.735304	10.75207	0.022523	5.61E-05
**'201--229'**	40	25.99012	11.39877	5.78185	8.809506	9.016858	0.030497	0.000153
**'230--248'**	10	16.98443	3.931343	0.414622	12.63846	16.15113	0.01631	0.00062
**'230--248'**	20	7.010958	4.070093	1.476124	1.464741	15.25916	0.011799	0.011798
**'230--248'**	25	16.97159	3.847963	2.258399	10.86522	77.22046	0.016858	0.000532
**'230--248'**	30	7.618501	4.057727	3.557413	0.003361	56.35768	0.016405	0.00017
**'230--248'**	40	8.203327	4.138975	3.392266	0.672086	21.95558	0.034975	0.007933
**'253--259'**	10	1.230907	0.591019	0.570112	0.069777	19.99956	0.025304	0.002031
**'253--259'**	20	4.987405	0.865273	0.477282	3.64485	2.174511	0.010544	0.000265
**'253--259'**	25	1.715016	0.601054	0.891275	0.222686	50.47242	0.010289	0.00078
**'253--259'**	30	4.990361	0.949038	0.49018	3.551144	3.263069	0.017051	0.000255
**'253--259'**	40	2.765737	0.989646	1.009795	0.766295	7.773119	0.014524	0.000698
**'260--267'**	10	2.281395	0.295859	0.65355	1.331986	64.43002	0.027471	0.002517
**'260--267'**	20	4.997779	0.389969	1.098467	3.509343	11.14922	0.055389	0.000489
**'260--267'**	25	1.895173	0.382106	0.970521	0.542545	70.51656	0.127955	0.010005
**'260--267'**	30	4.994166	0.438616	1.124587	3.430962	16.88531	0.169954	0.000806
**'260--267'**	40	2.612341	0.703146	0.952231	0.956964	6.60329	0.401401	0.010127
**'268--290'**	10	19.98936	3.20188	2.437972	14.34951	29.03236	0.103348	0.000341
**'268--290'**	20	16.6801	4.341242	1.968087	10.37077	12.11547	0.077264	0.000683
**'268--290'**	25	8.121149	3.669722	2.026721	2.424707	13.85346	0.091396	0.005726
**'268--290'**	30	19.97435	5.437536	1.491298	13.04552	3.298046	0.044486	0.000348
**'268--290'**	40	19.50655	5.39797	2.222682	11.8859	6.299242	0.089918	0.001058
**'301--320'**	10	9.141826	4.526354	2.756069	1.859402	28.90836	0.11511	0.01844

**'301--320'**	20	9.251962	4.879595	3.580618	0.79175	23.93735	0.163218	0.011087
**'301--320'**	25	17.99786	4.973871	3.246193	9.777794	17.91432	0.207508	0.000301
**'301--320'**	30	9.478263	5.262435	3.349824	0.866004	17.45138	0.316784	0.010639
**'301--320'**	40	9.625705	6.02867	3.004457	0.592577	12.66034	0.369426	0.008881
**'348--352'**	10	2.980665	0.915575	0.876617	1.188473	2.385025	0.016508	0.001394
**'348--352'**	20	2.262257	0.889539	0.435761	0.936956	20.02445	0.058132	0.022889
**'348--352'**	25	2.99874	0.849298	1.159268	0.990174	14.49275	0.052276	0.000634
**'348--352'**	30	2.118347	0.975318	1.132476	0.010553	11.76815	0.068655	0.001202
**'348--352'**	40	2.251269	1.012866	1.237882	0.000521	14.47525	0.115963	0.001739

Table S8. Fitted parameters for F61A mADA in substrate analog bound state.

Sequence	Temperature	N	A	B	C	k1	k2	k3
**15--28**	10	4.732544	1.511036	0.267674	2.953833	1.343028	0.004877	2.42E-07
**15--28**	20	8.010415	1.598766	4.005519	2.40613	2.345374	0.000437	1.5E-05
**15--28**	25	4.106593	1.62719	1.257034	1.222369	5.707137	0.002762	6.2E-06
**15--28**	30	4.514703	1.75465	1.521442	1.238611	8.54283	0.002444	0.001718
**15--28**	40	3.246225	1.723544	1.326532	0.196149	19.92807	0.012008	0.000628
**29--45**	10	13.99806	5.895049	1.900476	6.202534	2.597834	0.02185	0.00027
**29--45**	20	9.77997	6.522191	2.630318	0.627461	5.389089	0.019496	0.000373
**29--45**	25	13.99925	6.699756	2.323625	4.975866	10.25675	0.030418	0.000806
**29--45**	30	13.9815	7.360538	3.347043	3.273922	11.09654	0.028174	0.000208
**29--45**	40	10.41113	7.490885	2.705092	0.215155	11.80897	0.049054	6.55E-08
**46--62**	10	5.439491	1.787235	1.76089	1.891366	8.632209	0.077206	0.003012
**46--62**	20	7.934785	2.709048	1.575933	3.649804	3.412708	0.066755	0.00195
**46--62**	25	12.94787	2.801934	1.858171	8.287768	8.515335	0.079895	0.001028
**46--62**	30	10.90008	3.178661	2.371104	5.350318	9.168168	0.080373	0.002519
**46--62**	40	10.15128	3.527888	2.579611	4.043785	9.533313	0.102519	0.004797

**63--74**	10	0.947694	0.827766	0.108687	0.011241	20.091	0.017073	0.001214
**63--74**	20	4.481948	0.876074	0.292023	3.313851	24.41652	0.003858	0.000296
**63--74**	25	3.811592	0.907945	1.056411	1.847236	24.68005	0.003217	3.06E-05
**63--74**	30	8.984452	1.051188	0.36257	7.570693	17.96552	0.013395	0.000258
**63--74**	40	8.194757	1.153609	1.763145	5.278004	22.63216	0.004706	0.000187
**86--97**	10	4.305153	2.476173	1.474649	0.35433	10.38548	0.0225	1.62E-05
**86--97**	20	9.999017	2.690366	1.157415	6.151235	12.43723	0.028764	0.000529
**86--97**	25	9.99679	2.654435	1.483546	5.858809	14.29861	0.037757	0.000698
**86--97**	30	9.995978	3.044003	1.775375	5.176599	13.76899	0.031963	0.001042
**86--97**	40	6.157733	3.139567	2.391851	0.626315	12.5346	0.043539	0.002413
**109--131**	10	16.7533	8.11474	8.184542	0.454022	2.507578	0.000825	1.03E-07
**109--131**	20	16.80503	8.520608	8.04589	0.238531	5.536817	0.001166	1.31E-07
**109--131**	25	16.0703	8.654138	6.992718	0.423443	10.28182	0.001561	4.66E-08
**109--131**	30	16.95737	9.469242	7.465553	0.022572	9.582571	0.001451	9.08E-07
**109--131**	40	15.50426	9.185299	5.997288	0.321677	10.69627	0.001575	5.22E-08
**132--144**	10	10.93506	0.924473	0.274051	9.736535	18.25576	0.040296	0.000131
**132--144**	20	5.780794	0.957473	0.315405	4.507916	75.23661	0.068715	0.00142
**132--144**	25	2.866571	1.063867	1.798678	0.004026	17.61084	0.013527	0.000223
**132--144**	30	10.98533	1.139043	1.525438	8.320846	50.25869	0.031937	0.000343
**132--144**	40	10.95988	1.164578	1.511713	8.283592	28.26473	0.095002	0.000622
**155--163**	10	3.024006	2.175314	0.212557	0.636135	1.79893	6.58E-05	2.69E-05
**155--163**	20	3.80506	2.420014	0.696586	0.68846	2.834109	0.000166	0.000158
**155--163**	25	5.557425	2.747706	1.62409	1.185629	6.816725	2.39E-05	2.04E-05
**155--163**	30	5.999979	3.12161	2.878347	2.12E-05	7.560022	0.000102	2.76E-07
**155--163**	40	5.999998	3.419123	2.580872	2.44E-06	9.20697	0.0002	1.94E-07
**167--179**	10	6.08545	2.694607	1.381682	2.009161	1.740298	0.007983	6.62E-09

**167--179**	20	5.656087	2.816275	2.589894	0.249919	2.849139	0.006882	5.69E-05
**167--179**	25	5.881765	2.880702	2.721746	0.279317	3.877813	0.010486	3.21E-05
**167--179**	30	6.616491	3.134112	2.994229	0.48815	5.875413	0.014738	1.88E-08
**167--179**	40	6.230132	4.137483	1.718786	0.373862	4.044277	0.071269	6.08E-08
**180--200**	10	7.057241	4.335672	2.631133	0.090436	16.39838	0.015005	4.23E-05
**180--200**	20	8.577388	4.571847	1.172768	2.832773	9.833394	0.087538	0.0098
**180--200**	25	9.387766	4.580616	2.45058	2.356569	11.22325	0.069214	0.005193
**180--200**	30	10.31529	4.994286	2.836992	2.484014	10.14947	0.100631	0.007365
**180--200**	40	17.85198	5.074261	3.45523	9.322492	12.48269	0.166997	0.001145
**201--229**	10	25.93023	9.209731	4.250883	12.46962	12.14244	0.016718	9.31E-05
**201--229**	20	25.98844	10.1497	5.459114	10.37962	11.40206	0.016177	0.000103
**201--229**	25	25.92396	10.83899	4.334689	10.75028	11.75195	0.0217	0.000699
**201--229**	30	25.98116	11.81909	4.974198	9.187873	12.02178	0.023124	0.001389
**201--229**	40	25.94944	11.98156	5.924496	8.043384	12.14388	0.030236	0.001773
**230--248**	10	9.875293	3.889877	0.46103	5.524387	12.88147	0.046931	0.007432
**230--248**	20	9.925692	4.307937	5.612183	0.005572	11.07364	0.022078	0.000761
**230--248**	25	16.86255	4.310975	4.738266	7.81331	67.53587	0.048304	0.000593
**230--248**	30	10.77329	4.767437	4.372373	1.633479	85.15839	0.063838	0.017436
**230--248**	40	15.05578	4.547935	4.928122	5.579718	20.03251	0.169658	0.002324
**253--259**	10	2.026296	0.635514	0.391427	0.999354	10.24305	0.017943	6.59E-09
**253--259**	20	1.685442	0.708291	0.528347	0.448804	19.9472	0.017489	0.001056
**253--259**	25	4.992886	0.848061	0.453773	3.691052	4.861345	0.015143	0.000297
**253--259**	30	2.37198	1.005906	0.990131	0.375942	4.041666	0.00923	0.00082
**253--259**	40	4.995762	1.068226	0.84611	3.081426	3.036562	0.022995	0.000363
**260--267**	10	2.639219	0.415622	1.06256	1.161037	21.99913	0.152689	0.004611
**260--267**	20	2.507733	0.763083	0.799177	0.945473	8.158804	0.312314	0.020362

**260--267**	25	3.172083	1.38684	0.983479	0.801765	2.165822	0.056758	0.000729
**260--267**	30	2.635723	1.303895	0.722908	0.60892	7.320164	0.208635	0.047741
**260--267**	40	4.988425	1.501243	0.930828	2.556353	22.39297	0.24318	0.000279
**268--290**	10	6.364557	3.623704	2.611931	0.128923	19.59947	0.017895	2.9E-05
**268--290**	20	8.791773	4.315697	3.336134	1.139942	8.517422	0.015687	3.72E-09
**268--290**	25	8.931228	5.269421	3.151245	0.510563	2.845841	0.009855	5.72E-07
**268--290**	30	11.13335	6.190406	4.675415	0.267533	2.96385	0.009828	3.81E-05
**268--290**	40	11.87958	6.079462	4.941817	0.858303	3.756299	0.0114	2.33E-08
**301--320**	10	8.537665	4.393288	2.46985	1.674526	16.24382	0.103636	0.012105
**301--320**	20	17.98244	4.695871	3.475455	9.811113	19.83277	0.1667	0.000239
**301--320**	25	17.74012	4.99856	3.358084	9.383481	20.52513	0.297909	0.000314
**301--320**	30	17.75037	5.343492	3.179079	9.227798	16.89514	0.393869	0.000561
**301--320**	40	10.75218	6.752504	1.86082	2.138852	11.46155	0.404014	0.005214
**348--352**	10	2.999399	0.720388	0.177414	2.101598	1.417098	0.047472	0.001748
**348--352**	20	2.048628	0.807536	1.23748	0.003613	5.658936	0.017191	0.000719
**348--352**	25	2.103166	0.834699	1.127519	0.140948	9.121901	0.036336	0.001847
**348--352**	30	2.980614	0.814087	1.103019	1.063509	15.51347	0.067149	0.000588
**348--352**	40	2.997961	0.968983	0.981211	1.047768	12.55768	0.162225	0.000329

Table S9. Fitted parameters for F61A mADA in tight inhibitor bound state.

Sequence	Temperature	N	A	B	C	k1	k2	k3
15--28	10	1.836335	1.09676	0.705834	0.03374	19.80967	0.052008	0.001449
15--28	20	4.603955	1.621685	0.345928	2.636342	2.434967	0.010063	0.000231
15--28	25	10.70137	1.59186	0.758275	8.351239	4.561499	0.005458	9.84E-05
15--28	30	3.316649	1.606166	1.360761	0.349721	11.49331	0.005031	0.000418

15--28	40	4.939385	1.675543	1.337756	1.926085	19.86491	0.014815	0.000572
29--45	10	11.42951	6.397932	2.15375	2.877827	2.344559	0.019333	1.76E-05
29--45	20	10.36367	6.758101	2.333304	1.272264	7.914194	0.023403	0.001367
29--45	25	10.17306	6.805223	3.100393	0.267448	11.10499	0.023656	1.13E-08
29--45	30	10.52388	7.106636	3.245495	0.171748	13.40045	0.024239	2.77E-05
29--45	40	12.51664	7.942252	2.706453	1.867937	12.57588	0.040478	1.22E-09
46--62	10	12.94748	1.697984	1.849744	9.399747	10.05845	0.091099	0.000563
46--62	20	12.97719	2.51324	1.79847	8.66548	4.431921	0.073117	0.00077
46--62	25	7.163614	2.711248	1.988192	2.464174	6.073845	0.060406	0.004406
46--62	30	7.394137	2.924793	2.160738	2.308605	10.33765	0.072566	0.006112
46--62	40	12.93801	3.474383	2.908375	6.555253	10.1229	0.099235	0.001223
63--74	10	4.283126	0.845217	1.213289	2.224621	57.35242	0.000432	0.000265
63--74	20	8.94266	0.839034	0.241087	7.862539	13.68434	0.036589	0.000185
63--74	25	8.988277	0.815441	0.122917	8.04992	21.30715	0.027248	0.000377
63--74	30	3.252235	0.916667	0.402997	1.932571	50.97265	0.011981	0.001863
63--74	40	2.590518	0.893924	0.517226	1.179368	13.64626	0.027206	0.007106
86--97	10	4.244516	2.49922	1.545992	0.199303	19.70872	0.027702	0.000678
86--97	20	9.999712	2.65431	1.648971	5.696431	15.79851	0.051553	0.000661
86--97	25	9.958517	2.824726	2.146426	4.987365	20.047	0.028507	0.000536
86--97	30	9.990231	2.971236	1.975066	5.043929	16.74768	0.039062	0.000853
86--97	40	9.995525	3.457938	2.551573	3.986013	10.14572	0.043934	0.000187
109--131	10	17.96738	8.414732	9.522133	0.030511	2.29409	0.000818	2.19E-05
109--131	20	17.95348	8.697742	9.235791	0.019945	5.913596	0.000871	1.45E-06
109--131	25	16.31963	8.832187	6.911995	0.57545	4.329247	0.001232	2.22E-07
109--131	30	15.17352	8.724893	5.926828	0.521794	11.37358	0.001932	5.24E-05
109--131	40	16.73366	9.542364	6.694405	0.496886	10.69707	0.001419	7.41E-05
132--144	10	4.737197	1.251849	1.763973	1.721375	79.85741	0.001265	0.001265
132--144	20	10.88588	1.209049	1.127912	8.548919	14.45461	0.021344	0.00045
132--144	25	10.96312	1.206953	1.76138	7.994784	49.3655	0.019305	0.000302

132--144	30	5.056325	1.364079	1.795617	1.896629	50.93563	0.026022	0.001447
132--144	40	5.819601	1.288117	1.895701	2.635782	19.33942	0.091186	0.003157
155--163	10	2.765466	1.475573	1.111673	0.17822	19.84545	0.006684	8.6E-05
155--163	20	3.752716	1.988768	0.880788	0.88316	2.120373	0.001996	0.00198
155--163	25	5.960793	1.883444	0.843684	3.233665	3.511635	0.011076	0.000439
155--163	30	3.431787	1.869081	1.391834	0.170872	9.395683	0.012292	0.008095
155--163	40	5.999216	1.972836	1.422193	2.604187	16.09705	0.045582	0.000838
167--179	10	10.39316	2.832308	1.232397	6.328452	2.163156	0.011048	0.000117
167--179	20	10.98407	2.864795	1.581453	6.537818	6.840719	0.019564	0.000598
167--179	25	6.095228	2.888505	2.700274	0.50645	8.2227	0.019756	0.000253
167--179	30	5.913644	2.93168	2.81408	0.167884	19.77191	0.027032	0.000443
167--179	40	6.018938	3.298192	2.693639	0.027107	19.79513	0.055205	0.000288
180--200	10	17.94652	4.511158	0.795233	12.64013	75.78631	0.024095	0.000486
180--200	20	14.98857	4.43343	1.677215	8.877927	25.76596	0.055906	0.000792
180--200	25	8.324371	4.436602	1.579544	2.308225	82.51371	0.06351	0.009475
180--200	30	9.085839	4.620103	2.098247	2.367488	80.44755	0.06849	0.006619
180--200	40	10.25304	5.054413	2.528502	2.670128	15.99715	0.130689	0.007308
201--229	10	25.92761	9.066096	4.774264	12.08725	12.30589	0.029292	6.31E-05
201--229	20	14.8904	10.24237	4.535332	0.112698	9.910504	0.034823	0.000139
201--229	25	15.19525	10.64987	4.489889	0.055493	10.02388	0.036655	0.000142
201--229	30	15.8085	11.06333	4.510546	0.234629	12.09478	0.032111	8.46E-05
201--229	40	25.98823	12.76853	4.201126	9.018572	9.559561	0.019245	0.000799
230--248	10	16.94783	3.889092	0.307985	12.75075	11.92394	0.05918	0.000307
230--248	20	16.99961	4.135644	0.288997	12.57497	14.3286	0.039709	0.000842
230--248	25	16.89661	3.885243	2.241949	10.76942	71.75227	0.016165	0.00048
230--248	30	7.885576	4.010341	3.794272	0.080963	68.58898	0.01675	0.006931
230--248	40	16.97062	4.236807	3.357632	9.376184	17.95937	0.049761	0.000389
253--259	10	1.463666	0.718512	0.436405	0.308748	16.30597	0.00883	6.51E-07
253--259	20	4.950511	0.672122	0.461906	3.816483	8.784217	0.025547	7.84E-05

253--259	25	4.990496	0.663334	0.546587	3.780575	17.27616	0.026329	0.000192
253--259	30	2.050749	0.745811	0.756104	0.548834	19.98276	0.016912	0.001542
253--259	40	4.988881	0.898061	1.005338	3.085482	6.929945	0.022545	0.000221
260--267	10	1.575045	0.430496	0.573846	0.570703	70.49947	0.007547	0.007547
260--267	20	2.206542	0.396128	1.067515	0.7429	20.04388	0.043677	0.000758
260--267	25	2.510918	0.430167	0.996346	1.084404	20.00542	0.083245	0.00111
260--267	30	4.944165	0.479435	0.947445	3.517284	18.76734	0.116628	0.000442
260--267	40	2.30514	0.69959	0.749222	0.856329	72.84966	0.309758	0.007225
268--290	10	19.99211	3.655982	1.746713	14.58941	72.805	0.058566	0.000247
268--290	20	19.9665	3.810105	2.293227	13.86316	12.004	0.096005	0.000216
268--290	25	19.9597	3.705692	2.447821	13.80618	30.31529	0.090321	0.000275
268--290	30	8.704781	4.11835	2.362205	2.224226	13.67139	0.089437	0.003322
268--290	40	19.99757	5.268804	1.985751	12.74302	9.015893	0.076217	0.000999
301--320	10	17.99639	4.451681	3.119737	10.42497	21.28064	0.059051	0.000497
301--320	20	17.92077	4.733364	3.518934	9.668473	16.52785	0.151296	0.000253
301--320	25	8.701668	4.52363	2.869511	1.308528	87.36345	0.341944	0.055864
301--320	30	9.663	5.142267	3.368504	1.152229	19.40379	0.319092	0.004123
301--320	40	10.69824	6.400512	2.361834	1.935897	12.22213	0.576767	0.003389
348--352	10	2.965099	0.792507	0.222498	1.950095	1.572377	0.028166	0.00207
348--352	20	2.148629	0.811562	0.53091	0.806157	4.027231	0.040419	0.011876
348--352	25	2.128604	0.838187	1.289856	0.000562	8.756896	0.036995	0.000594
348--352	30	2.166369	0.90112	1.200126	0.065123	14.38995	0.062662	0.002418
348--352	40	2.130706	1.011136	1.116751	0.002819	19.95957	0.142915	0.002677

Table S10. Apparent activation energies of HDX for 17 peptides in substate free (apo), DAA bound (ES), and pentostatin bound (ETS) WT mADA. (The other peptides do not exhibit statistically significant rate constants due to rapid exchange.) All activation energies were calculated using $B/N_T*k_2+C/N_T*k_3$ as weighted average rate constant from two independent HDX experiments.

Peptide	Ea(apo)	Ea(ES)	Ea(ETS)	ΔEa(ES-apo)	ΔEa(ETS-apo)	ΔEa(ETS-ES)
15-28	15(0.6)	13.42(2.1)	14.21(1.9)	-1.58(2.2)	-0.79(2.0)	0.79(2.8)
29-45	4.7(1.4)	4.19(0.8)	6.32(1.2)	-0.51(1.6)	1.62(1.8)	2.13(1.4)
46-62	6.2(1.5)	9.35(1.3)	12.21(1.3)	**3.15(2.0)**	**6.01(2.0)**	**2.86(1.8)**
63-74	14.1(2.3)	15.15(1.2)	17.02(1.6)	1.05(2.6)	2.92(2.8)	1.87(2.0)
86-97	4.8(2.2)	7.22(0.3)	9.56(0.8)	**2.42(2.2)**	**4.76(2.3)**	**2.34(0.9)**
109-131	-1(2)	1.35(0.3)	1.61(0.9)	**2.35(2.0)**	**2.61(2.2)**	0.26(1.0)
132-144	12.4(1.9)	20.64(0.4)	21.28(1.7)	**8.24(1.9)**	**8.88(2.6)**	0.64(1.8)
155-163	16.7(0.3)	22.77(2.9)	21.29(4.2)	**6.07(2.9)**	**4.59(4.2)**	-1.48(5.1)
167-179	11.5(1.2)	10.47(0.9)	12.44(1.8)	-1.03(1.5)	0.94(2.2)	1.97(2.0)
180-200	11.5(0.9)	16.23(3)	16.65(2.9)	**4.73(3.1)**	**5.15(3.0)**	0.42(4.1)
201-229	9.3(1.2)	2.84(1)	0(0.9)	**-6.46(1.6)**	**-9.3(1.5)**	**-2.84(1.3)**
230-248	15.7(0.8)	17.67(0.5)	18.15(1.7)	**1.97(1.0)**	**2.45(1.9)**	0.48(1.8)
253-259	2.4(1.1)	7.17(1)	6.99(2.3)	**4.77(1.5)**	**4.59(2.6)**	-0.18(2.5)
260-267	18.5(2.3)	21(2.3)	24.11(1.2)	2.5(3.3)	5.61(2.6)	3.11(2.6)
268-290	4.4(1.4)	5.83(0.1)	7.42(0.4)	**1.43(1.4)**	**3.02(1.4)**	**1.59(0.4)**
301-320	4.3(0.6)	15.5(2.2)	16(2)	**11.2(2.3)**	**11.7(2.1)**	0.5(3.0)
348-352	11.1(1.3)	17.73(0.8)	16.87(1.2)	**6.63(1.5)**	**5.77(1.7)**	-0.86(1.4)

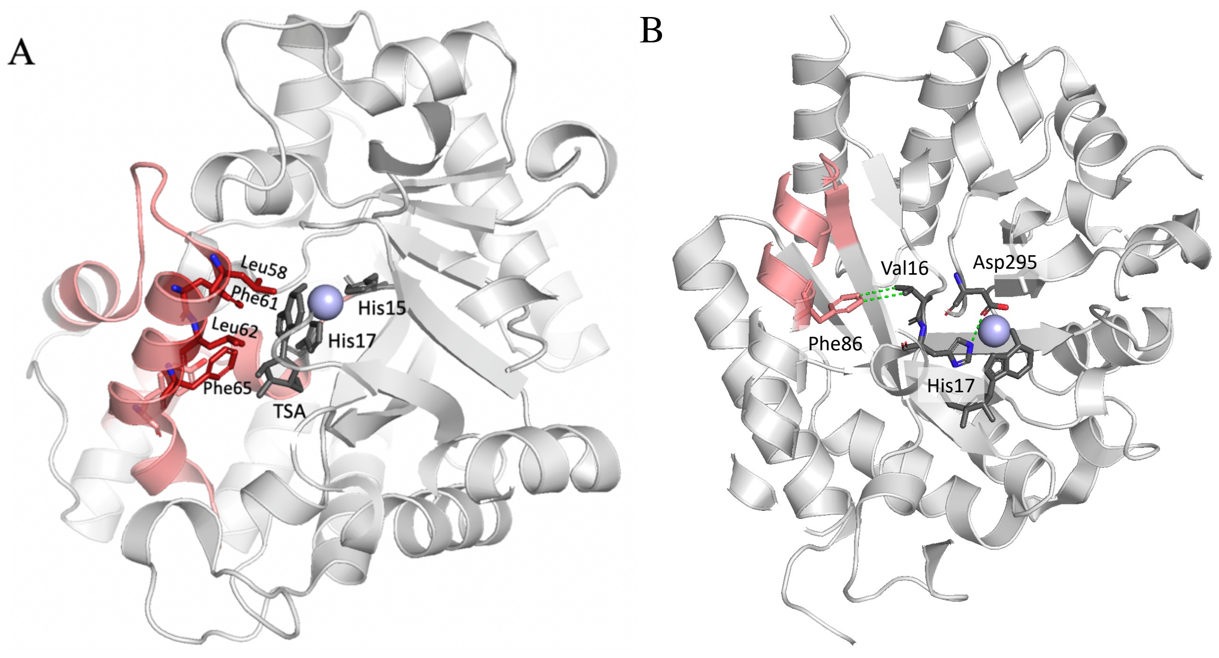

Figure S9 (A) A highly hydrophobic region of the mADA structure that is comprised of Leu58, Phe61, Leu62 and Phe65 within peptide **46-62**. (B) Phe86 from peptide 86-97 interacts with Val16, adjacent to one of the Zn ligands His17.

Table S11. Apparent activation energies of HDX for 17 peptides in substate free (apo), DAA bound (ES), and pentostatin bound (ETS) F61A mADA. (The other peptides do not exhibit statistically significant rate constants due to rapid exchange.) All activation energies were calculated using $B/N_T*k_2+C/N_T*k_3$ as weighted average rate constant from two independent HDX experiments.

Peptide	Ea(apo)	Ea(ES)	Ea(ETS)	ΔEa(ES-apo)	ΔEa(ETS-apo)	ΔEa(ETS-ES)
15-28	7.4(1.3)	11.06(4.3)	14.51(2.49)	3.66(4.5)	7.11(2.8)	3.45(4.95)
29-45	4.7(1.4)	6(1.3)	5.97(0.42)	1.3(1.9)	1.27(1.5)	-0.03(1.34)
46-62	1.4(1.5)	4.67(1.8)	3.16(2.63)	3.27(2.3)	1.76(3)	-1.51(3.18)
63-74	8(0.8)	11.42(1.8)	14.54(5.42)	3.42(1.9)	6.54(5.5)	3.12(5.7)
86-97	3.4(1.6)	6.96(1.4)	5.06(1.7)	3.56(2.1)	1.66(2.3)	-1.9(2.17)
109-131	-0.8(1.6)	4(2)	2.3(1.6)	4.8(2.6)	3.1(2.3)	-1.7(2.56)
132-144	11.7(1.5)	14.18(2.4)	19.93(3.36)	2.48(2.8)	8.23(3.7)	5.75(4.12)
155-163	13.4(0.8)	12.35(5.4)	16.75(5.41)	-1.05(5.5)	3.35(5.5)	4.4(7.65)
167-179	9.5(0.5)	13.22(1)	14.11(0.43)	3.72(1.1)	4.61(0.7)	0.89(1.06)
180-200	8.4(1.2)	15.27(0.3)	14.88(1.81)	6.87(1.2)	6.48(2.2)	-0.39(1.84)
201-229	3.1(1)	5.78(0.8)	-2.55(1.47)	2.68(1.3)	-5.65(1.8)	-8.33(1.65)
230-248	12.4(0.6)	15.19(1.2)	12.81(2.81)	2.79(1.3)	0.41(2.9)	-2.38(3.050
253-259	1.9(3.8)	5.43(2.5)	9.98(2.14)	3.53(4.5)	8.08(4.4)	4.55(3.28)
260-267	15.4(2.5)	19.4(3.1)	19.28(2.51)	4(4)	3.88(3.5)	-0.12(3.990
268-290	-0.7(1.6)	1.23(2.4)	1.96(2.6)	1.93(2.9)	2.66(3.1)	0.73(3.55)
301-320	7.2(1.3)	13(1.1)	15(1.2)	5.8(1.7)	7.8(1.8)	2(1.6)
348-352	11.5(0.3)	16.07(1.4)	16.31(0.44)	4.57(1.5)	4.81(0.5)	0.24(1.5)

Table S12. Differences in apparent activation energies of HDX for 17 peptides between F61A and WT mADA at apo state and pentostatin bound state. All activation energies were calculated using $B/N_T*k_2+C/N_T*k_3$ as weighted average rate constant from two independent HDX experiments.

	ΔEa(F61A-WT) Apo state	ΔEa(F61A-WT) ETS state	ΔEa(F61A-WT) ES state
15-28	-7.6(1.4)	0.3(3.1)	-2.36(4.8)
29-45	0(2.0)	-0.35(1.3)	1.81(1.5)
46-62	-4.8(2.1)	-9.05(2.9)	-4.68(2.2)
63-74	-6.1(2.4)	-2.48(5.7)	-3.73(2.1)
86-97	-1.4(2.7)	-4.5(1.9)	-0.26(1.4)
109-131	0.2(0.69)	0.69(1.9)	2.65(2.0)
132-144	-0.7(2.4)	-1.35(3.8)	-6.46(2.4)
155-163	-3.3(0.9)	-4.54(6.8)	-10.42(6.1)
167-179	-2(1.3)	1.67(1.8)	2.75(1.3)
180-200	-3.1(1.5)	-1.77(3.4)	-0.96(3.0)
201-229	-6.2(1.6)	-2.55(1.7)	2.94(1.3)
230-248	-3.3(1.0)	-5.34(3.3)	-2.48(1.3)
253-259	-0.5(4.0)	2.99(3.2)	-1.74(2.7)
260-267	-3.1(3.4)	-4.83(2.8)	-1.6(3.9)
268-290	-5.1(2.1)	-5.46(2.6)	-4.6(2.4)
301-320	2.9(1.4)	-1(2.3)	-2.5(2.5)
348-352	0.4(1.3)	-0.56(1.2)	-1.66(2.6)

Dataset S1 (separate file). HDX Data Table.

Dataset S2 (separate file). HDX Summary Table.

SI References

10. S. Gao *et al.*, Hydrogen–Deuterium Exchange within Adenosine Deaminase, a TIM Barrel Hydrolase, Identifies Networks for Thermal Activation of Catalysis. *J. Am. Chem. Soc.* **142**, 19936-19949 (2020).
11. E. J. Thompson, A. Paul, A. T. Iavarone, J. P. Klinman, Identification of Thermal Conduits That Link the Protein–Water Interface to the Active Site Loop and Catalytic Base in Enolase. *J. Am. Chem. Soc.* **143**, 785-797 (2021).
